# Transcriptomic Architecture of Metabolic Dysfunction-Associated Steatotic Liver Disease (MASLD) Risk in Mexican Americans

**DOI:** 10.3390/cells15171592

**Published:** 2026-09-01

**Authors:** Satish Kumar, Miriam Aceves, Lorena Guerra, Jose Granados, Earl Novilla, Felicia Juarez, Tolulope Oluwadairo, Ana C. Leandro, Marcelo Leandro, Juan Peralta, Sarah Williams-Blangero, John Blangero, Joanne E. Curran

**Affiliations:** 1 Department of Human Genetics and South Texas Diabetes and Obesity Institute, University of Texas Rio Grande Valley, College of Sciences, McAllen, TX 78504, USA; 2 Department of Human Genetics and South Texas Diabetes and Obesity Institute, University of Texas Rio Grande Valley, College of Sciences, Brownsville, TX 78520, USA

**Keywords:** MASLD, hepatic steatosis, hepatocyte, transcriptomics, human iPSC

## Abstract

**Highlights:**

**What are the main findings?**

- Hepatic steatosis (neutral lipid) measures in iPSC-derived hepatocyte cultures showed statistically significant additive genetic heritability at baseline (*h*^2^ = 0.44, *p* ≤ 0.05) and post-lipid challenge (*h*^2^ = 0.42, *p* ≤ 0.05).
- Transcriptome-wide association analysis identified 1070 genes at baseline and 1229 genes post-lipid challenge whose expression was significantly (|β|≥0.24, Bonferroni p≤0.001) associated with hepatic steatosis measures.

**What are the implications of the main findings?**

- Functional annotation and pathway enrichment analysis of these genes suggest fatty acid/cholesterol uptake, de novo lipogenesis, and high-turnover cellular stress responses driving the steatosis risk, whereas endosomal and autophagic clearance, cellular cytoskeleton, and hepatocytes’ epithelial integrity play a protective role.
- Our work demonstrated an epidemiological-scale use of an iPSC-derived hepatocyte model for mapping the transcriptomic determinants of MASLD-associated hepatic steatosis risk.

**Abstract:**

Hispanics of Mexican American descent in South Texas show a very high prevalence of MASLD, with some studies reporting rates as high as 50% in adults. However, assessment of genetic risk factors underlying this prevalence is complicated by a high co-occurrence of other metabolic disorders and variable endogenous and exogenous environmental risk factors. To map the transcriptomic architecture of MASLD hepatic steatosis risk, we conducted an epidemiological-scale investigation using human induced pluripotent stem cell (iPSC)-derived hepatocyte cultures from 193 participants in our longitudinal South Texas Family Study (STFS). iPSC-based models offer greater power to map genetic risk factors by experimentally controlling for confounding organismal and environmental factors. We combined transcriptome-wide gene expression analysis with high-content cellular measurements of neutral lipids to define a core hepatic steatosis MASLD phenotype at baseline (vehicle-treated) and following a lipid challenge. The additive genetic heritability of hepatic steatosis measures was 0.44 (*p*-value = 0.03) at baseline and 0.42 (*p*-value = 0.03) at post-lipid challenge. Multivariable linear regression comparing each gene’s expression against hepatic steatosis measures identified 1070 genes at baseline and 1229 genes post-lipid challenge, whose expression showed a transcriptome-wide statistically significant association (standardized |*β*| ≥ 0.24; Bonferroni-corrected *p*-value ≤ 0.001) with baseline and post-lipid challenge hepatic steatosis measures, respectively. Functional annotation and pathway enrichment analyses of these genes implicated a broad range of hepatocellular functions, mapping an overall transcriptomic architecture of MASLD-associated steatosis risk in Mexican Americans. The genes whose expression was positively correlated with hepatic steatosis measures suggest a direct role of variation in fatty acid (FA) and cholesterol uptake, de novo lipogenesis (DNL), and carbohydrate shunts in hepatic steatosis risk, as well as a cellular stress-associated and high-turnover metabolic state marked by elevated FA-oxidation and ketogenesis. In contrast, the genes whose expression was inversely correlated with hepatic steatosis measures suggest a significant role of the cellular cytoskeleton, hepatocyte epithelial integrity, and endosomal and autophagic clearance machinery in steatosis risk.

## 1. Introduction

Hepatic steatosis is the defining, often asymptomatic feature of steatotic liver disease (SLD; formerly referred to as fatty liver disease or FLD), and is characterized by an excess accumulation of lipids (typically >5%) within hepatocytes. Hepatic steatosis in the presence of one or more cardiometabolic risk factors, such as obesity, insulin resistance/type 2 diabetes, high blood pressure, hypertriglyceridemia, or dyslipidemia, is classified as metabolic dysfunction-associated steatotic liver disease (MASLD), formerly known as non-alcoholic fatty liver disease (NAFLD). MASLD is the most common form of SLD, affecting over 30% of adults globally [1,2]. The condition can potentially lead to a more profound disease state of metabolic dysfunction-associated steatohepatitis (MASH; formerly referred to as non-alcoholic steatohepatitis or NASH), in which there is liver inflammation/damage that may be reflected in hepatic fibrosis (including severe cirrhosis at the extreme). MASH can lead to liver failure or hepatocellular carcinoma (HCC). The rate of HCC has increased dramatically across the globe in the past 20 years, including in the United States, where the increase is particularly apparent in Hispanic populations [3]. Hispanics are disproportionately impacted by MASLD and have the highest observed prevalence in the world; 45% of those with MASLD are estimated to have MASH [4]. Previous studies have reported prevalences of MASLD as high as 50% in adult Hispanics of Mexican American descent in South Texas [5].

A complex mix of genetic and environmental factors contributes to an individual’s risk of developing hepatic steatosis. The tightly linked biological factors include metabolic syndrome, obesity, type 2 diabetes, and high cholesterol. However, demographic characteristics (e.g., sex, age, and ethnicity), behavioral and lifestyle-related variables (e.g., dietary behavior and physical activity), and both endogenous (e.g., infectious agents such as hepatitis viruses, microbiome variability) and exogenous environmental factors (e.g., exposure to pollutants, contaminants, and toxins) also play a role, complicating the assessment of genetic risk and the identification of associated genes.

Hepatocytes constitute about 80% of both the cell population and total volume of the human liver. To support the liver’s massive metabolic load, hepatocytes have evolved highly specialized membrane transport that enables a complex vesicle-based protein and lipid uptake, sorting, storage, and release mechanisms that are superimposed upon an organized cytoskeletal scaffold and directly orchestrate hepatic lipid homeostasis. However, the precise genetic architecture of this cellular machinery and its overall contribution to SLD risk remain largely undefined [6]. The main sources of hepatocyte lipids include: (1) receptor-mediated uptake of albumin-bound fatty acids (FAs) released into circulation from lipolysis, (2) lipoproteins and chylomicron remnants internalized via low-density lipoprotein receptor (LDL-R), (3) high-density lipoprotein (HDL)-derived cholesteryl esters (CEs) uptake via class B scavenger receptor, and (4) triacylglycerols (TAGs)/triglycerides synthesized by de novo lipogenesis in hepatocytes [7]. Hepatocytes re-package a large part of this lipid, primarily TAG and CEs, into very low-density lipoprotein (VLDL) particles and secrete it into the general circulation to deliver lipid to peripheral tissues. The pathways of VLDL assembly and trafficking through the endoplasmic reticulum (ER), cytosol, and trans-Golgi network (TGN), and eventual secretion into the bloodstream plausibly play a role in hepatic steatosis but remain to be fully understood. The transmembrane 6 superfamily member 2 (*TM6SF2*) E167K variant (rs58542926) that impairs the assembly and secretion of VLDL was found to be associated with increased MASLD and MASH risk [8]. The utilization of lipids in hepatocytes includes fatty acid oxidation, mainly in the mitochondria [9]. The key gatekeeping enzymes, carnitine palmitoyltransferase (CPT) and glycerol-3-phosphate acyltransferase (GPAT), likely commit the acyl-CoAs into the β-oxidation or glycerolipid synthesis pathways, respectively. However, the mechanisms that regulate this partitioning in hepatocytes are yet to be identified. Stimulated in part by the insulin pathway, hepatocytes package a significant amount of TAGs in cytosolic lipid droplets (LDs). While LD accumulation is the prerequisite step for hepatic steatosis, the protein and lipid composition of LDs appears to be the critical factor in disease progression [10]. The impairment in LD breakdown has emerged as a significant contributing factor for MASLD risk. Well-known MASLD risk-associated variants in patatin-like domain 3, 1-acylglycerol-3-phosphate O-acyltransferase (*PNPLA3*), hydroxysteroid 17-beta dehydrogenase 13 (*HSD17B13*), and perilipin 2 (*PLIN2*) seem to affect LD processing in hepatocytes [11,12,13].

To map the transcriptomic architecture of human hepatic steatosis risk in Mexican Americans and bridge the crucial knowledge gaps in the understanding of hepatocellular lipid homeostasis, we performed an epidemiological-scale investigation using human induced pluripotent stem cell (iPSC)-derived hepatocyte cultures generated for 193 Mexican American participants in our longitudinal South Texas Family Study (STFS). We quantitatively measured these well-characterized hepatocyte cultures for hepatic steatosis (neutral lipid accumulation) and transcriptome-wide gene expression, both at baseline and post-lipid (fatty acid) challenge to identify genes whose expression showed significant predictive value for hepatic steatosis. Because donor-derived epigenetic memory is largely erased during reprogramming, particularly in blood cell-derived iPSCs, the iPSC-based model offers a powerful advantage for mapping genetic determinants of phenotypic variation by effectively controlling for confounding organismal and environmental factors [14].

## 2. Materials and Methods

The study utilized existing cryopreserved iPSC lines that were generated from lymphoblastoid cell lines (LCLs) of the Mexican American participants in our longitudinal STFS cohort. The iPSC reprogramming and validation methodologies used were described in our previous publications [15,16,17]. The STFS began in 1991 (originally called the San Antonio Family Heart Study [18]) and now includes 2621 adult individuals. Samples from 193 STFS participants were randomly chosen and analyzed in this study. The mean age and the age standard deviation of the 193 subjects at the time of sample collection were 43.2 ± 12.7 years; 120 were females, and 73 were males. Since its inception, the STFS has recruited and maintained slightly more female than male participants, and the sex distribution of the 193 participants reflects this recruitment scenario. Genetic relatedness (up to the 10th degree) between all pairs of individuals was empirically estimated by the methodology described in [19] using existing whole-genome sequence (WGS) data.

The differentiation of validated iPSC lines into functional hepatocyte cultures was achieved using a three-stage differentiation protocol that we published previously [20]. The generated hepatocyte cultures from each individual were characterized by immunocytochemistry (ICC) analysis of the hepatocyte markers, alpha-fetoprotein (AFP), albumin (ALB), E-cadherin (CDH1), and hepatocyte nuclear factor-4 alpha (HNF4A), and then lipid-challenged to model MASLD-like conditions. Genome-wide transcriptomic and functional profiles of the generated hepatocytes were also confirmed using bulk mRNA sequencing (mRNAseq) data generated from baseline (control) hepatocyte cultures.

### 2.1. Lipid Challenge

To model MASLD-like conditions, the generated hepatocyte cultures were lipid-challenged using a mix of saturated and unsaturated fatty acids. One half of the culture on day 20 of differentiation was vehicle-treated (baseline), using 0.4% fatty acid-free bovine serum albumin (BSA; GoldBio, St. Louis, MO, USA) in hepatocyte maturation medium and a lipid challenge was performed on the other half using a mix of 200 µM palmitic acid and 25 µM oleic acid (both from MilliporeSigma, St. Louis, MO, USA) conjugated to fatty acid-free BSA in the hepatocyte maturation medium to induce hepatic steatosis stress. The hepatocyte maturation medium contained HCM^TM^ Hepatocyte Culture Medium BulletKit^TM^ (Lonza Bioscience, Walkersville, MD, USA) supplemented with 100 nM Dexamethasone (MilliporeSigma, St. Louis, MO, USA), 20 ng/mL rHuman FGF-4, 10 ng/mL rHuman HGF, and 50 ng/mL rHuman Oncostatin M (PeproTech, Thermo Fisher Scientific, Cranbury, NJ, USA). After a 24 h treatment, both vehicle-treated baseline and lipid-challenged cultures were quantitatively assessed for hepatic steatosis (neutral lipid accumulation in hepatocytes) using high-content screening analysis and for genome-wide gene expression by bulk mRNAseq analysis. Cell viability of both baseline and lipid-challenged hepatocyte cultures after the 24 h treatments was assessed using acridine orange and propidium iodide (AO/PI) staining and the CellDrop automated cell counter (DeNovix Inc., Wilmington, DE, USA).

### 2.2. Quantitative Measurement of Hepatic Steatosis

To quantify the accumulation of neutral lipids in hepatocytes (hepatic steatosis), both baseline and lipid (FA)-challenged hepatocyte cultures were fixed using 4% *w*/*v* paraformaldehyde (PFA; MilliporeSigma, St. Louis, MO, USA) in Dulbecco’s phosphate-buffered saline without Ca^2+^ and Mg^2+^ (DPBS) for 15 min, permeabilized for 12 min with permeabilization buffer containing 0.2% *v*/*v* Triton X-100 in 1% *w*/*v* BSA (both from MilliporeSigma, St. Louis, MO, USA) in DPBS, and then stained with 5 μM PhenoVue 493 neutral lipid stain following the manufacturer’s instructions (Revvity, Waltham, MA, USA). Cell nuclei were counterstained with DAPI (4′, 6-diamidino-2-phenylindole; Invitrogen), and appropriate negative controls were included. The lipid-stained cultures were imaged immediately using a PerkinElmer Operetta (samples of 146 individuals) and a Revvity Operetta CLS (samples of 47 individuals) high-content screening system (Revvity, Waltham, MA, USA). The high-content quantitative assessment of steatosis in hepatocytes was performed by measuring the cytoplasmic average fluorescence intensity of the lipophilic stain per visual field across 8–9 fields in two independent replicates. The cell numbers were assessed by the nuclei count, and cells partially overlapping the image boundaries were removed from the quantification. The steatosis quantifications were performed using the PerkinElmer Operetta Harmony v4.1 and Revvity Operetta CLS Harmony v5.2 software (Revvity, Waltham, MA, USA), respectively. Lipid measures were inverse-normalized, and instrument IDs were included as covariates in the regression model as described in the statistical/association analysis section below.

### 2.3. Immunocytochemistry Analysis

All generated hepatocyte cultures were analyzed for the expression of hepatocyte markers to confirm differentiation using standard ICC techniques and the primary antibodies for AFP (mouse anti-human AFP, sc-8399, Santa Cruz Biotechnology, Inc., Dallas, TX, USA), ALB (mouse anti-human ALB, sc-271605, Santa Cruz Biotechnology, Inc., Dallas, TX, USA), CDH1 (rabbit anti-human CDH1, 3195, Cell Signaling Technology, Danvers, MA, USA), HNF4A (rabbit anti-human HNF4A, 3113, Cell Signaling Technology, Danvers, MA, USA), and secondary antibodies donkey anti-mouse Alexa Fluor™ 488 and donkey anti-rabbit Alexa Fluor™ 594 (R37114 and R37119, respectively; Invitrogen, ThermoFisher Scientific, Waltham, MA, USA). In each ICC analysis, cell nuclei were counterstained with DAPI (Invitrogen), and appropriate negative controls were included. Cells were imaged immediately after ICC staining on the PerkinElmer Operetta or the Revvity Operetta CLS high-content screening system (Revvity, USA).

### 2.4. RNA Extraction and mRNA Sequencing

Total RNA was extracted from each hepatocyte culture at baseline and post-lipid challenge using the RNeasy Mini Kit (Qiagen, Germantown, MD, USA) following the manufacturer’s protocol. The quality and quantity of the extracted RNA samples were assessed using a NanoDrop 2000 Spectrophotometer (Thermo Fisher Scientific, Waltham, MA, USA) and an Agilent 4150 TapeStation system (Agilent, Santa Clara, CA, USA).

Genome-wide mRNA sequencing of both baseline and lipid-challenged hepatocyte samples was performed on an Illumina NovaSeq 6000 instrument using the Illumina Stranded mRNA Prep, Ligation Kit. The reagents supplied in the Illumina Stranded mRNA Prep, Ligation Kit (Illumina, Inc., San Diego, CA, USA) and 0.5–1 µg of high-quality total RNA from each sample were used to prepare mRNA sequencing libraries. First, oligo-dT beads were used to enrich poly-A-tailed mRNA from the total RNA sample. The enriched mRNA was then fragmented into molecules of approximately 200–600 base pairs using divalent cations and elevated temperature. The first-strand cDNA was synthesized from fragmented mRNA molecules using reverse transcriptase and random primers, followed by the second-strand cDNA synthesis using DNA polymerase I and RNase H. The synthesized cDNA fragments were end-repaired, and adaptors were ligated. The resulting cDNA libraries were purified, enriched by PCR, and then sequenced on an Illumina NovaSeq6000 instrument.

### 2.5. RNA Sequencing Analysis

The binary base call (bcl) sequencing files were converted and demultiplexed into raw fastq files using the Illumina bcl2fastq2 software v2.20.0 (Illumina, Inc., San Diego, CA, USA). After pre-alignment quality control, the raw sequence files were aligned to the human genome assembly GRCh38 (hg38) and mapped to the human RefSeq transcripts using StrandNGS software v4.1 (Strand Life Sciences Pvt. Ltd., Bangalore, India). The aligned reads were filtered based on read quality metrics (i.e., average base quality ≥ 20; number of “N”s ≤ 1; alignment score ≥ 95; mapping quality ≥ 40; read length ≥ 20; and reads failing the vendor’s quality check were removed), and log transformation and “DESeq” normalization were applied. Known genes/mRNAs with a normalized read count (NRC) ≥ 10 in at least 25% of samples per condition, as described in the results, were considered expressed for downstream differential gene expression and/or association analysis. Additionally, the gene expression data were transformed into Z-scores for the association analysis.

### 2.6. Differential Gene Expression Analysis

For the comparative analysis between representative iPSC lines and the generated hepatocyte cultures, moderated *t*-statistics and fold change analysis were performed to identify significantly differentially expressed genes. To quantify hepatocellular response to lipid challenge, pairwise gene expression change (log ratio/delta) was calculated between baseline and post-lipid challenge conditions per individual using the linear mixed model approach. Statistical significance of the paired change (delta) against a null hypothesis of no change (delta = 0) was assessed using Wald’s test. Confounding variables such as age, sex, and mRNAseq batch were included in the model.

### 2.7. Statistical/Association Analysis

A variance-component (VC) linear mixed model approach was used to estimate additive genetic influence on baseline and post-lipid-challenged hepatocyte lipid measures. In a simple model, variances or covariances between relatives as a function of the genetic relationships can be specified, and the proportion of phenotypic variance that is attributed to additive genetic effect (i.e., heritability: *h*^2^) can be estimated from the components of variance. To address non-normality, hepatocyte lipid measures (phenotypic data) were inverse-normalized, and the DEseq-normalized, log-transformed gene expression data were converted into Z-scores. All association analyses were performed using the VC linear mixed model approach as implemented in the computer program Sequential Oligogenic Linkage Analysis Routines (SOLAR v8.1.1) and adjusted for covariate effects such as age, sex, phenotyping instrument ID, and mRNAseq batch as described in the results [21]. All gene and phenotypic correlations were corrected for multiple testing using the Bonferroni correction [22].

### 2.8. Functional Annotations and Enrichment Analyses

Functional annotations and enrichment analyses of the gene sets of interest were performed using the Gene Ontology (GO) resource [23,24], the Kyoto Encyclopedia of Genes and Genomes (KEGG) database [25,26,27], the Ingenuity Pathway Analysis (IPA) platform (QIAGEN Digital Insights, Redwood City, CA, USA) and “Enrichr (https://maayanlab.cloud/Enrichr/)” and “ShinyGO v0.85” gene set enrichment analysis web tools [28,29] (accessed June–July 2026). The “Enrichr” tool implements several enrichment scores as described by Chen et al. [28]. We ranked our “Enrichr” results based on computed Fisher exact test *p*-values. In “ShinyGO” enrichment analyses, FDR is calculated based on the nominal *p*-value from the hypergeometric test. For “ShinyGO” enrichment analyses, we used FDR-corrected *p*-values ≤ 0.05 for statistical significance. To map gene sets to KEGG pathway maps, the KEGG Mapper v5 web tool was used. In IPA, right-tailed Fisher’s exact test FDR-corrected *p*-values were used for enrichment significance, and the direction of functional change was assessed by the activation *z*-score as described in Kramer et al. [30].

## 3. Results

### 3.1. Large-Scale Generation of Validated Hepatocyte Cultures and Hepatic Steatosis Measures

**Figure 1:**
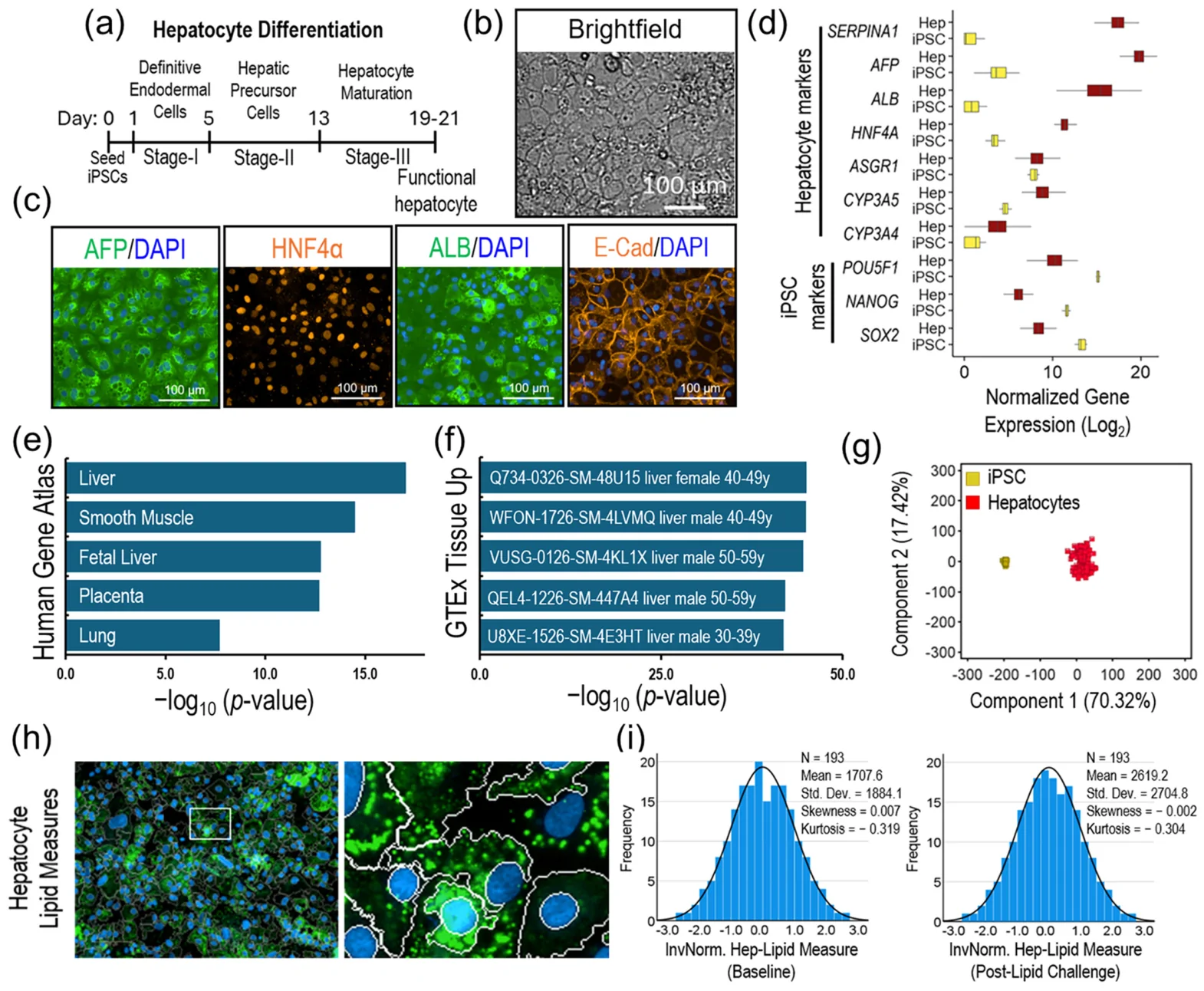
Generation of validated hepatocyte culture and hepatic steatosis measurements. (**a**) Schematic diagram of hepatocyte differentiation. (**b**) Brightfield image panel showing the morphology of the generated hepatocytes. (**c**) Representative ICC image panel of the hepatic markers AFP, ALB, HNF4α, and CDH1 (E-Cad) in the generated hepatocytes. (**d**) Gene expression box plots of key hepatocyte and pluripotency markers in iPSCs and generated hepatocytes. (**e**,**f**) Top five Human Gene Atlas and GTEx tissue upregulated gene sets, which were significantly enriched in the generated hepatocytes’ upregulated transcriptome. (**g**) PCA of iPSCs and the generated hepatocyte (hepC) differentially expressed transcriptome. (**h**) Representative image panel showing high-content screening analysis of hepatocyte lipid measures. (**i**) Summary statistics histograms of hepatocyte lipid measures at baseline and post-lipid challenge.

To confirm that the iPSC-derived hepatocyte cultures were uniform across all samples, possessed transcriptional and functional characteristics of human hepatocytes, and maintained those characteristics during the experiments, we performed ICC analysis of hepatic markers and a comparative transcriptomic analysis of the representative 18 iPSC lines and baseline hepatocyte cultures generated from all 193 individuals. All hepatocyte cultures exhibited characteristic hepatocyte morphology (i.e., flat, polygonal cells with distinct round nuclei and a cobblestone appearance) and expressed AFP, ALB, HNF4A, and CDH1 (E-Cad) protein markers, as shown in Figure 1a–c. The high correlation between the generated hepatocyte-expressed transcriptome (genes with an NRC ≥ 10 in at least 25% of baseline hepatocyte samples) from all 193 individuals (*r*^2^ [95% CI] = 0.911 ± 0.005) confirms that we achieved a highly uniform differentiation across all samples, and sample uniformity was maintained throughout the experiments. The comparative analysis of the iPSC and hepatocyte transcriptomes identified 9875 significantly differentially expressed (DE) genes (moderated *t*-statistics, FDR-corrected *p*-value ≤ 0.05, and absolute fold change [|FC|] ≥ 2.0), with 4595 genes being significantly upregulated in generated hepatocytes. These upregulated genes showed top (statistically significant) enrichment in the Human Gene Atlas gene set for the liver (*p*-value = 9.3 × 10^−18^) and the Genotype-Tissue Expression (GTEx) database (tissue upregulated gene sets) for human adult liver samples (*p*-value ≤ 1.36 × 10^−42^). We have also previously shown that our iPSC-derived hepatocytes exhibit cytochrome P450 activity without any stimulation [31]. To assess hepatocyte-specific characteristics, we evaluated the expression of hepatocyte- and pluripotency-specific genes within the iPSC and generated hepatocyte transcriptomes. The hepatocyte marker genes—serpin family A member 1 (*SERPINA1*, which encodes alpha-1 antitrypsin [AAT], a crucial protective protein predominantly produced in the liver); *AFP*; *ALB*; *HNF4A*; asialoglycoprotein receptor 1 (*ASGR1*); cytochrome P450 family 3 subfamily A member 5 (*CYP3A5*); and cytochrome P450 family 3 subfamily A member 4 (*CYP3A4*)—were all expressed in the generated hepatocytes and showed a significant upregulation as compared to the iPSC lines, except for *ASGR1*, which was also highly expressed in iPSCs. The expression of the three core pluripotency genes—POU class 5 homeobox 1 (*POU5F1*), nanog homeobox (*NANOG*), and SRY-box transcription factor 2 (*SOX2*)—was significantly downregulated (FC-abs ≥ 30) in generated hepatocytes (Figure 1d–f). Additionally, the principal component analysis (PCA) of the 9875 DE genes shows that about 70% of the differences in gene expression between iPSCs and the generated hepatocytes were due to the cellular transitions/differentiation process (Figure 1g). Overall, these results show that our iPSC-derived cultures possessed hepatocyte characteristics, were uniform in their transcriptional and functional profiles across different samples, and maintained these characteristics throughout the experiments.

For the hepatic steatosis assessment, the iPSC-derived hepatic cultures were quantitatively measured for cellular neutral lipid accumulation (hepatic steatosis), both at baseline (vehicle-treated) and post-lipid challenge, using high-content screening techniques. Measurements were performed across 8–9 visual fields in two independent replicates per sample per condition. The mean and standard deviation (mean ± standard deviation) of the raw hepatic steatosis measures were 1707.556 ± 1884.122 at baseline and 2619.161 ± 2704.817 post-lipid challenge. The mean hepatic steatosis measures were inverse-normalized to address non-normality. For the inverse-normalized hepatic steatosis measures, the skewness values were 0.007 and 0.002, and the kurtosis values were −0.319 and −0.304 for the baseline and post-lipid-challenged samples, respectively (Figure 1h–i). The additive genetic heritability of hepatic steatosis measures was 0.44 (*p*-value = 0.03) at baseline and 0.42 (*p*-value = 0.03) for the post-lipid-challenged samples.

### 3.2. Identification of Genes Whose Expression Was Significantly Correlated with Hepatic Steatosis Measures

To identify genes whose expression levels were significantly correlated with hepatic steatosis measures at baseline and post-lipid challenge, we conducted multivariable linear regression on each gene’s expression and the hepatic steatosis measures at baseline and post-lipid challenge, respectively, while including age, sex, hepatic steatosis phenotyping instrument ID, and mRNA sequencing batch as covariates in the model.

### 3.3. Genetics of Baseline Variation in Hepatic Steatosis

**Figure 2:**
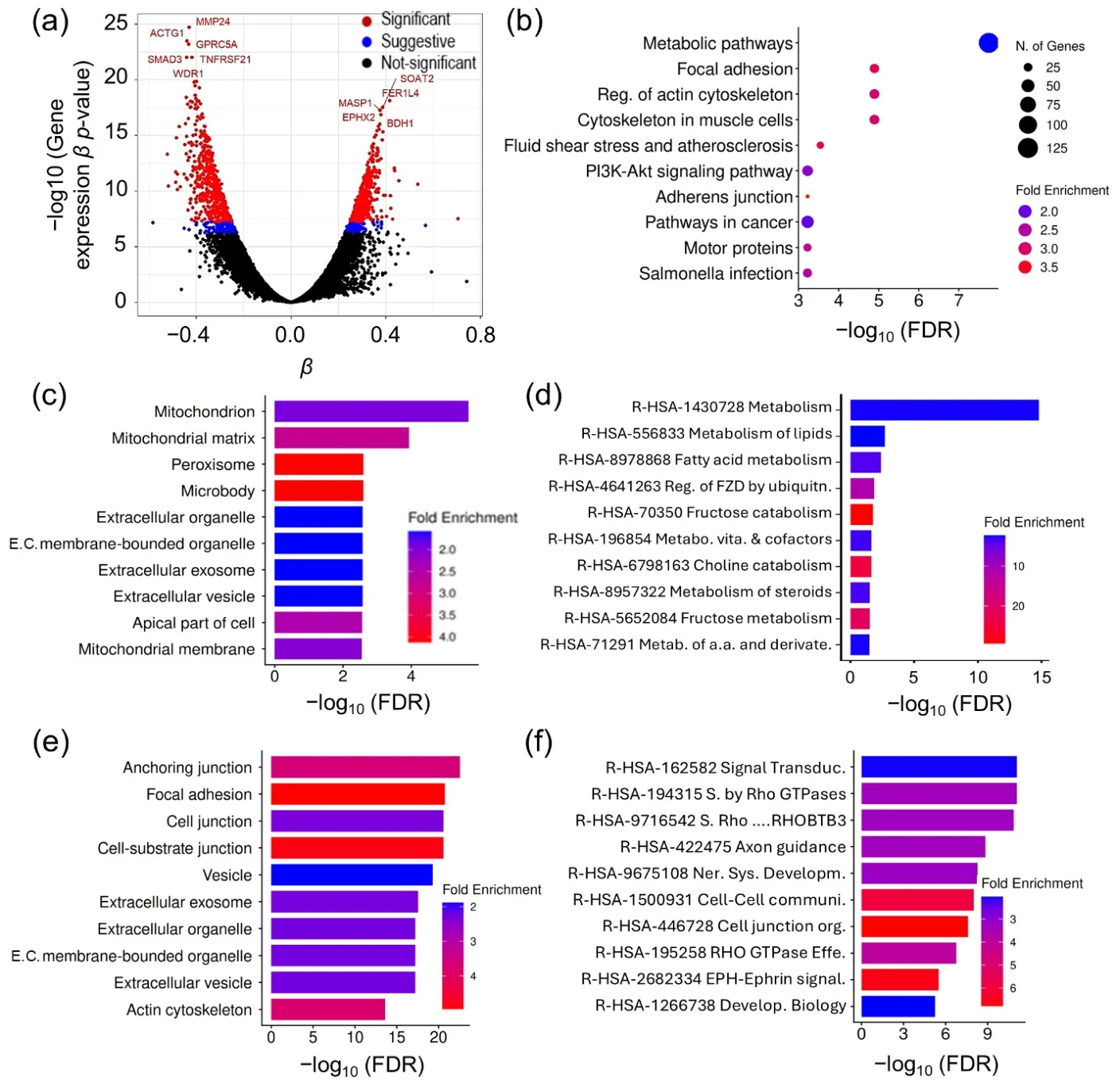
Functional annotation and pathway enrichment of genes that were significantly associated with baseline hepatic steatosis measures. (**a**) Volcano plot showing genes that were significantly associated with baseline hepatic steatosis. (**b**) KEGG pathway enrichment of the 1070 genes that were significantly associated with baseline hepatic steatosis. (**c**,**d**) GO cellular component and Reactome pathway enrichment of the 495 genes whose expression was directly correlated with baseline hepatic steatosis measures, respectively. (**e**,**f**) GO cellular component and Reactome pathway enrichment of the 575 genes whose expression was inversely correlated with baseline hepatic steatosis measures, respectively.

The expression of 1070 genes showed a transcriptome-wide statistically significant association (standardized |*β*| range = 0.25−0.70; Bonferroni-corrected *p*-value ≤ 0.001) with hepatic steatosis measures at baseline (Figure 2a; Supplementary Table S1). KEGG pathway annotation of these genes showed a significant enrichment in metabolism, cell cytoskeleton, and inflammatory immune response-related pathways (Figure 2b). To further dissect the functional signature of these genes, we performed separate enrichment analyses of genes whose expression was positively (directly) or negatively (inversely) correlated with the baseline hepatic steatosis measures into GO cellular component and Reactome pathway gene sets. The 495 genes whose expression was directly correlated with baseline steatosis measures were significantly enriched in mitochondria, peroxisome, and extracellular-exosome-related GO cellular components, and carbohydrate and lipid metabolism-related Reactome pathways (Figure 2c,d). This subset included several key genes heavily implicated in cellular lipid homeostasis such as: fatty acid binding protein 1 (*FABP1*) and solute carrier family 27 member 2 (*SLC27A2*; also known as *FATP2*), which are involved in fatty acid uptake and intracellular fatty acid transport [32,33]; StAR-related lipid transfer domain containing 4 (*STARD4*) is a intracellular cholesterol transporter [34]; MLX interacting protein like (*MLXIPL*; also known as *ChREBP*), a master transcription factor that activates the expression of glycolytic and lipogenic genes in response to high glucose [35]; elongation of very-long-chain fatty acids protein 6 (*ELOVL6*), which elongates saturated and monounsaturated fatty acids, contributing directly to the lipogenic pool [36]; sterol O-ascyltransferase 2 (*SOAT2*; also known as *ACAT2*), involved in cholesterol esterification for lipid droplet and very low-density lipoprotein (VLDL) assembly [37]; microsomal triglyceride transfer protein (*MTTP*), which is critical for assembling and secreting ApoB-containing lipoproteins [38]; and acetyl-CoA carboxylase beta (*ACACB*), which is localized to the outer mitochondrial membrane, and generates a localized pool of malonyl-CoA that effectively shuts down the carnitine shuttle, halting β-oxidation and forcing fatty acids toward esterification into TAGs [39]. Apart from these plausible hepatic steatosis risk candidates (core lipid metabolism and lipogenesis-associated genes), the 495 genes also included those likely to be involved in molecular signatures secondary to the hepatocyte response to higher lipid load. These include genes involved in elevated mitochondrial and peroxisomal β-oxidation, pyruvate and glucose shunt, ketogenesis, and immune and inflammatory signaling cellular functions. Additionally, genes associated with cell survival, differentiation, and tissue architecture, including hedgehog signaling, fibroblast and insulin-like growth factor signaling, CCAAT enhancer binding protein alpha (*CEBPA*) regulated adipogenesis and lipid homeostasis, and steatosis-induced endoplasmic reticulum (ER) stress, were also enriched (Supplementary Table S2). The 575 genes whose expression was inversely correlated with baseline hepatic steatosis measures were significantly enriched in cell junction, extracellular exosome, and cell cytoskeleton-related GO cellular components and Rho GTPase signaling and cell cytoskeleton and motor system-related Reactome pathways (Figure 2e,f). The key genes enriched in this category are associated with non-vesicular lipid transfer and cell membrane contact sites; lipid flippases and scramblases; lipid metabolism; vesicular carrier dynamics and Rab GTPases; actin cytoskeleton and remodeling factors; microtubules and molecular motors; intermediate filaments; vesicle mobility; membrane curvature and invagination; and lipid raft scaffolding (Supplementary Table S3).

Altogether, these results suggest that cell cytoskeleton and cellular lipid transport machineries, particularly cell junction integrity, cytoskeletal dynamics, and localized membrane scaffolding, including endosomal and autophagic clearance machinery, constitute a prominent molecular signature that is inversely correlated with hepatic steatosis risk.

### 3.4. Genetics of Post-Lipid-Challenge Variation in Hepatic Steatosis

**Figure 3:**
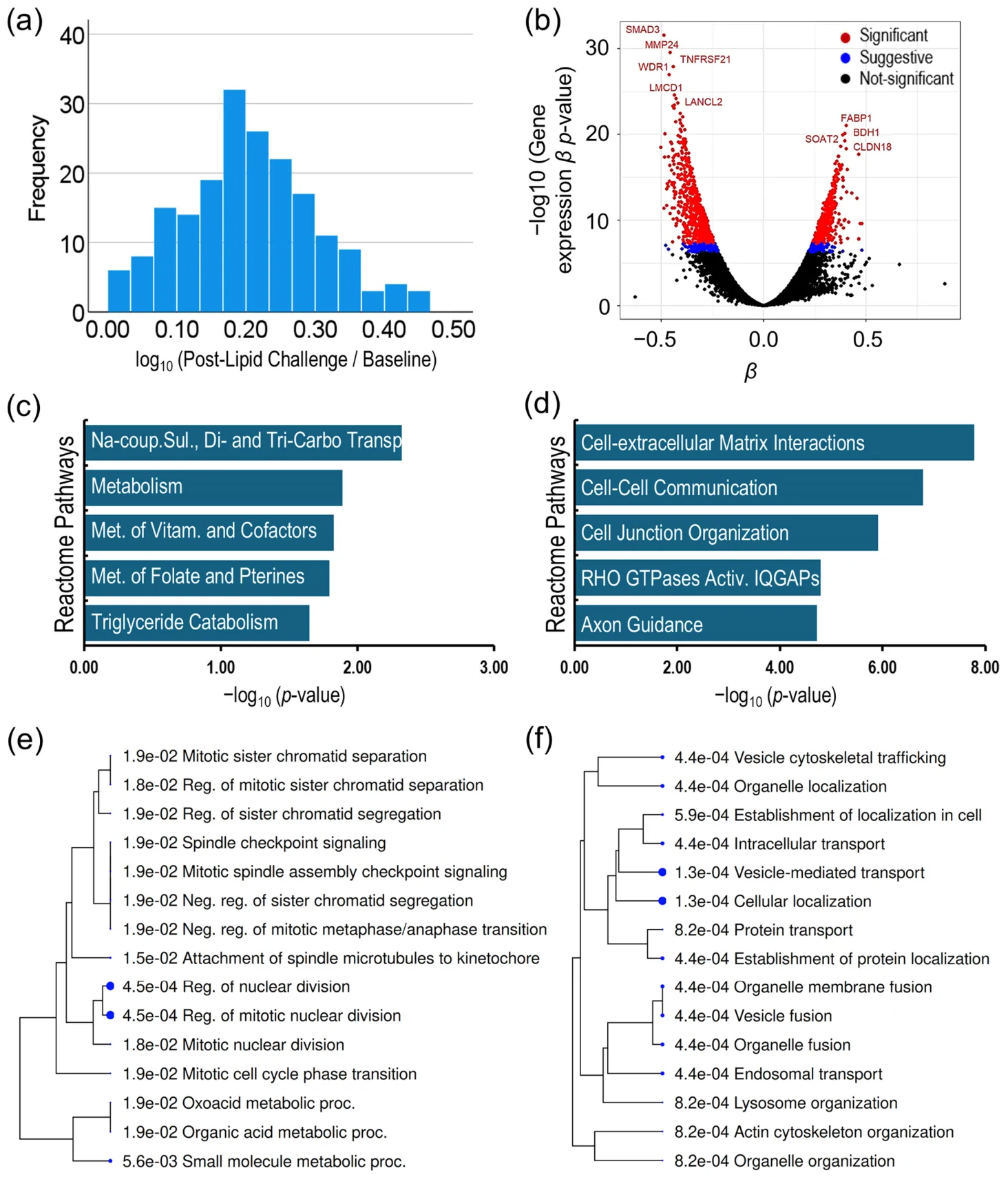
Functional annotation and pathway enrichment of genes that were significantly associated with post-lipid-challenge hepatic steatosis measures. (**a**) Log_10_ ratio histogram of post-lipid-challenge to baseline hepatic steatosis measure across samples from 193 studied individuals. (**b**) Volcano plot showing genes that were significantly associated with post-lipid-challenge hepatic steatosis. (**c**,**d**) Reactome pathway enrichment of shared genes directly (**c**) and inversely (**d**) correlated with hepatic steatosis measures across baseline and post-lipid-challenge analyses. (**e**,**f**) GO biological processes enrichment analysis of 421 genes significantly associated with post-lipid-challenge hepatic steatosis measures, showing (**e**) genes whose expression was directly and (**f**) genes whose expression was inversely correlated with post-lipid-challenge hepatic steatosis measures.

A lipid challenge was performed for 24 h to induce hepatic steatosis stress. Post-lipid-challenge hepatocytes from most individuals showed an increase in hepatic steatosis, albeit with individual-specific quantitative variability, as shown in Figure 3a. Cell viability of both baseline and post-lipid-challenge hepatocyte cultures was assessed and did not show any significant difference. The genetic association analysis of the post-lipid-challenge hepatic steatosis measures and gene expression identified 1229 genes, whose expression showed a transcriptome-wide statistically significant association (standardized |*β*| range = 0.24−0.50; Bonferroni-corrected *p*-value ≤ 0.001) with post-lipid-challenge hepatic steatosis measures (Figure 3b; Supplementary Table S4). The identified genes showed 65% overlap with the genes identified in baseline association analysis, suggesting a significant functional overlap in genetic risk factors of baseline and lipid stress-induced hepatic steatosis. To investigate the post-lipid-challenge change in this functional overlap, we focused on 51 genes whose post-lipid-challenge expression correlation with hepatic steatosis increased by an absolute Δ*β* ≥ 0.05 compared to baseline. Reactome pathway enrichment of this gene set, comprising 19 directly correlated and 32 inversely correlated genes, is shown in Figure 3c,d. The focal genes included in this set were *RAB26*, *FABP1*, solute carrier family 13 member 5 (*SLC13A5*), transmembrane protein 97 (*TMEM97*), and EBP cholestenol delta-isomerase (*EBP*), whose expression was directly correlated with hepatic steatosis, and Keratins (*KRT8*, *KRT18*, and *KRT7*), Actins (*ACTB* and *ACTG1*), and Tubulin (*TUBB3*), whose expression was inversely correlated with hepatic steatosis, suggesting a dysregulated lipid metabolism with increased lipid import, lipogenesis and lipophagy, alongside cellular cytoskeleton stress. The 421 new genes, which were significantly associated with post-lipid-challenge steatosis measures, were composed of 231 directly correlated and 190 inversely correlated genes. The 231 directly correlated genes were significantly enriched in the cell cycle-related and organic acid metabolic GO biological processes (Figure 3e), suggesting an increase in cell proliferation and altered citric acid cycle, fatty acid oxidation, and reactive oxygen species, a typical condition in metabolic stress. The 190 inversely correlated genes showed significant enrichment in actin cytoskeleton organization, lysosome organization, endosomal transport, vesicle fusion, vesicle-mediated transport, and vesicle cytoskeleton trafficking (Figure 3f), further strengthening the evidence that hepatocyte cytoskeleton and membrane transport that enable complex vesicle-based protein and lipid uptake, sorting, storage, and release mechanisms have a major role in hepatic steatosis risk and is adversely impacted by cellular lipid stress in hepatocytes.

### 3.5. Hepatocellular Response to Lipid Challenge

**Figure 4:**
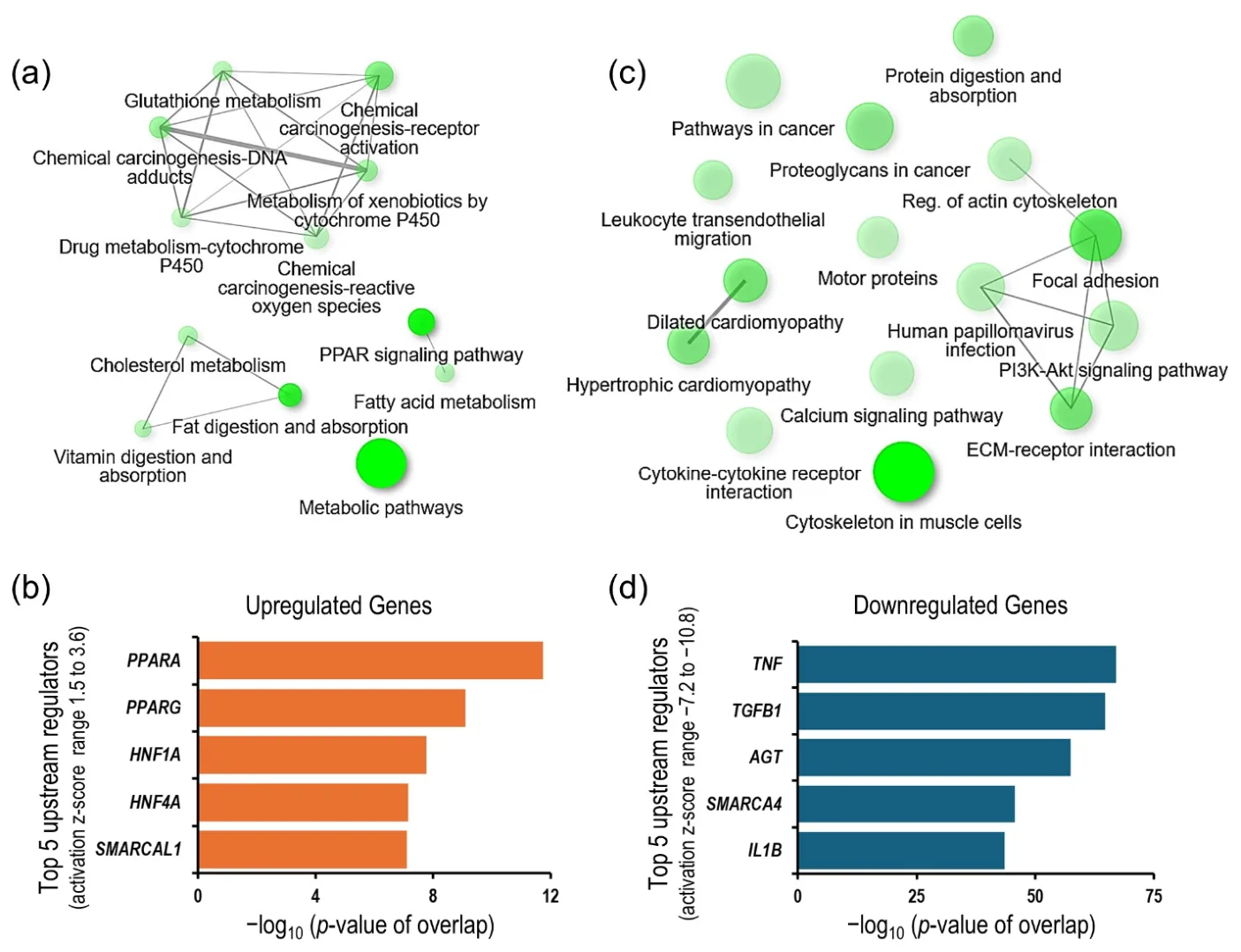
GO term and upstream regulator enrichment analysis of genes that were DE between baseline and post-lipid-challenged hepatocytes. (**a**) Network of the top 15 GO biological processes enriched in 216 upregulated genes. (**b**) Top 5 upstream regulators that were significantly enriched and predicted to be activated in 216 upregulated genes. (**c**) Network of the top 15 GO biological processes enriched in 902 downregulated genes. (**d**) Top 5 upstream regulators that were significantly enriched but predicted to be inhibited in 902 downregulated genes. Network Legend (**a**,**c**): Two nodes (biological processes) are connected if they share 20% or more genes. Darker nodes are more significantly enriched gene sets. Bigger nodes represent larger gene sets. Thicker edges represent more overlapping genes.

To further investigate the hepatocyte’s response to lipid overload challenge and the role it may have in hepatic steatosis risk, we performed differential gene expression analysis between baseline and post-lipid-challenged hepatocytes’ expressed transcriptomes (genes with NRC ≥ 10 in at least 25% of baseline and/or post-lipid-challenged hepatocytes). A total of 1150 genes were found to be significantly DE (Wald *χ*^2^ (1) = 10.297, FDR-corrected *p* ≤ 0.005, and absolute change in expression ≥ 0.2 standard deviation units) between the two conditions. The 228 genes that were upregulated showed significant enrichment in PPAR signaling and fatty acid, cholesterol, vitamin, and xenobiotic metabolism-related KEGG pathways, suggestive of lipid overload stress (Figure 4a).

The upstream regulator analysis of this gene set using the IPA platform showed a significant enrichment and predicted activation of peroxisome proliferator-activated receptor alpha and gamma (*PPARA* and *PPARG*); HNF1 homeobox A (*HNF1A*); *HNF4A*; and SNF2-related chromatin remodeling annealing helicase 1 (*SMARCAL1*) upstream regulators, a gene expression profile suggestive of elevated lipid catabolism (mitochondrial and peroxisomal β—oxidation, ketogenesis, and lipid flux), fatty acid uptake, glycerolipid synthesis, and lipid droplet formation, and replication and transcriptional stress, the conditions commonly seen in MASLD (Figure 4a,b). The post-lipid-challenge change in *PLIN2* expression showed the highest statistical significance (FDR-corrected *p* = 5.3 ×10^−160^). Altered turnover of *PLIN2* is a well-known risk factor for MASLD and MASH [12].

GO biological process enrichment (Figure 4c) and upstream regulator (Figure 4d) analyses of the 922 genes that were significantly downregulated in post-lipid-challenged hepatocytes suggest significant suppression of extracellular matrix (ECM) organization, ECM–receptor interactions, actin cytoskeleton dynamics, and motor protein-mediated intracellular transport and pro-inflammatory processes. These alterations are plausibly driven by cellular lipid stress, and loss of structural and transport machinery likely exacerbates lipid accumulation and worsens metabolic dysfunction in hepatocytes.

The DE genes showed a limited overlap (~12%) with genes significantly associated with baseline and post-challenge hepatic steatosis measures. Despite this low quantitative overlap, key biological pathways were captured: genes driving lipid metabolism and metabolic homeostasis were upregulated, while those regulating cell adhesion, cytoskeleton dynamics, integrin signaling, ECM interactions, and TGF-β signaling were downregulated. Overall, this suggests that while DE analysis highlights key functional pathways, it is limited in capturing the full transcriptomic architecture underlying hepatic steatosis risk.

## 4. Discussion

In this study, we demonstrate an epidemiological-scale in vitro approach leveraging human iPSC-derived hepatocyte cultures to investigate the complex transcriptomic architecture of risk for MASLD. We integrated transcriptome-wide gene expression analysis with cellular lipid measures as a core hepatic steatosis MASLD phenotype at baseline (vehicle-treated) and post-lipid challenge to identify genes associated with hepatic steatosis risk. As discussed in the introduction section, the iPSC-based approach offers a powerful advantage for mapping genetic determinants of variation in hepatic steatosis by effectively controlling for confounding organismal and environmental factors [14].

We have shown that variations in hepatic steatosis (neutral lipid) measures in iPSC-derived hepatocyte cultures were heritable, and we have identified a comprehensive list of genes whose expression shows a statistically significant association with hepatic steatosis measures. The expression of 1070 genes at baseline and 1229 genes post-lipid challenge was significantly associated with baseline and post-lipid-challenge hepatic steatosis measures, respectively. The genes identified in these analyses encompass a vast array of hepatocellular functions that likely contribute to MASLD-associated hepatic steatosis risk.

An imbalance between lipid input and disposal is the primary driver of hepatic steatosis in MASLD [40]. Intracellular FA pools available for TAG esterification and LD accumulation are predominantly derived from three pathways: uptake of free non-esterified FAs from adipose tissue lipolysis, DNL, and receptor-mediated endocytosis of lipoprotein remnants [10]. Adipose tissue-derived FAs constitute the major source (~50–70%) of FAs esterified into TAGs. The hepatocellular uptake of this FA pool is mediated by transporter proteins encoded by *CD36*, *SLC27A2* (also known as *FATP2*), and *FABP1* [41,42]. In our human iPSC-derived hepatocytes, *CD36* was robustly expressed, while the expression levels of *SLC27A2* and *FABP1* were significantly associated with and directly correlated with both baseline and post-lipid-challenge hepatic steatosis measures. Furthermore, *FABP1* expression was significantly upregulated post-lipid challenge, underscoring its dynamic role in FA handling and transport in hepatocytes.

Besides these dedicated FA transporters, expression of several genes involved in hepatic cholesterol uptake, endocytosis, and intracellular transport showed a statistically significant association and positive correlation with hepatic steatosis measures at baseline and post-lipid challenge. These included: NPC1 like intracellular cholesterol transporter 1 (*NPC1L1*), which is localized to the canalicular membrane and mediates the reuptake/absorption of biliary cholesterol back into liver cells; *ASGR1*, which mediates serum lipoprotein clearance and hepatic cholesterol uptake; *TMEM97* that interacts with low-density lipoprotein receptor (LDLR) to facilitate the endocytosis and cellular uptake of cholesterol-rich LDL particles into hepatocytes; GRAM domain containing 1B (*GRAMD1B*), which is a member of the Aster protein family that binds accessible plasma membrane cholesterol and mediates non-vesicular cholesterol transport into the endoplasmic reticulum following cellular uptake; and *STARD4* a soluble sterol transfer protein that senses cellular cholesterol levels and shuttles cholesterol between cellular membranes to coordinate cellular cholesterol uptake and homeostasis [34,43,44,45,46,47,48]. Although we have not directly measured VLDL secretion in our hepatocyte culture, the expression of *SOAT2* (involved in packaging cholesterol into cholesteryl ester for lipid droplet and VLDL assembly [37]) and *MTTP* (critical for assembling and secreting ApoB-containing lipoproteins [38]) was also significantly positively correlated with hepatic steatosis measures.

As indicated above, DNL is another major source of FAs for hepatic lipid accumulation. Its contribution expands from ~5–10% in healthy fasting states to ~30–40% of the FA pool in cases of hyperinsulinemia, hyperglycemia, and MASLD [49,50,51]. Expression of the primary carbohydrate-sensing transcription factor *MLXIPL* (*ChREBP*), which regulates an entire suite of DNL genes including *ACACA*, *ACACB*, *FASN*, and *ELOVL6* [52], was significantly associated with and directly correlated with both baseline and post-lipid-challenge hepatic steatosis measures. A similar positive correlation was observed for several other DNL and LD formation pathway genes, including DNL related substrate precursor enzymes (*ACACB*, acyl-CoA synthetase short chain family member 2 [*ACSS2*], *SLC13A5*, and pyruvate dehydrogenase E1 subunit alpha 1 [*PDHA1*]), NADPH supply and FA elongation factor (isocitrate dehydrogenase 1 [*IDH1*], and *ELOVL6*), and TAG assembly enzymes (diacylglycerol O-acyltransferase 1 and 2 [*DGAT1* and *DGAT2*]).

Interestingly, the expression of several genes associated with β-oxidation and ketogenesis was also directly correlated with hepatic steatosis. These included *ACACB*, which downregulates β-oxidation and forces fatty acids toward esterification, as well as 3-hydroxy-3-methylglutaryl-CoA synthase 2 (*HMGCS2*), 3-hydroxybutyrate dehydrogenase 1 (*BDH1*), hydroxyacyl-CoA dehydrogenase (*HADH*), enoyl-CoA hydratase and 3-hydroxyacyl-CoA dehydrogenase (*EHHADH*), acyl-CoA dehydrogenase family member 11 (*ACAD11*), and acyl-CoA oxidase 2 (*ACOX2*), which are involved in fatty acid breakdown via oxidation and ketogenesis. This simultaneous activation of both pro-esterification and pro-oxidative/ketogenic pathways suggests a high-turnover metabolic condition that likely results from lipid overload in MASLD [53,54].

In addition to these core lipid homeostasis genes, expression of genes enriched in several other cellular functions that may contribute to or be secondary to lipid accumulation/overload also showed a significant positive correlation with hepatic steatosis in our analysis. These included genes involved in pyruvate and glucose shunts, in which ketohexokinase (*KHK*), aldolase B (*ALDOB*), and pyruvate dehydrogenase kinase 3 (*PDK3*) function to divert carbohydrates to DNL, whereas fructose-1,6-bisphosphatase 1 (*FBP1*) functions to divert glycolytic intermediates to gluconeogenesis [55,56,57], suggestive of hepatic carbohydrate overload and pathway dysregulation characteristic of MASLD. Additionally, genes associated with several other functions indicative of hepatic steatotic stress showed positive correlations, including those involved in inflammatory signaling and immune activation, Hedgehog signaling, growth factor and receptor signaling (regulating cell survival, differentiation, proliferation, and metabolic homeostasis), adipogenesis and lipid homeostasis transcription factors, transcription factors associated with the cellular stress response, and markers of ER stress (as listed in Supplementary Table S2).

Besides the pro-lipogenic and lipid homeostasis genes discussed above, we identified a substantial cohort of genes (575 at baseline and 653 at post-lipid challenge) whose expression was inversely correlated with hepatic steatosis measures. These genes overwhelmingly implicate cell cytoskeletal dynamics, ECM interaction, and epithelial integrity of hepatocytes in hepatic steatosis risk. We know that intracellular LD growth, trafficking, and turnover depend heavily on the dynamic rearrangement of the cell cytoskeleton and cell-junction complexes, as well as LD accumulation, which adversely impacts these structures, likely instigating a vicious cycle that contributes to lipid accumulation/steatosis [58,59,60,61,62,63]. We observed a significant inverse correlation between baseline and post-lipid-challenge hepatic steatosis measures and the expression of genes encoding key structural components of the cell cytoskeleton, including actin isoforms (actin beta [*ACTB*], actin gamma 1 [*ACTG1*], and actin alpha 1 [*ACTA1*]), tubulins (tubulin alpha 1a [*TUBA1A*], tubulin beta 3 class III [*TUBB3*], tubulin beta 6 class V [*TUBB6*]), intermediate filaments (keratin 8, 18, and 7 [*KRT8*, *KRT18*, and *KRT7*] and vimentin [*VIM*], neurofilament light chain [*NEFL*], and neurofilament medium chain [*NEFM*]), and motor proteins (myosin heavy chain 9 [*MYH9*], myosin VB [*MYO5B*], myosin IE [*MYO1E*], kinesin family member 3C [*KIF3C*], kinesin family member C3 [*KIFC3*], and dynactin subunit 1 [*DCTN1*]). Furthermore, expression of genes associated with focal adhesion, tight junction, membrane curvature and lipid raft scaffolding—such as integrin subunit beta 1 (*ITGB1*); integrin subunit alpha 2 and 3 (*ITGA2* and *ITGA3*); cadherin 2, 3, and 6 (*CDH2*, *CDH3*, and *CDH6*); catenin alpha 1 (*CTNNA1*); and junction plakoglobin (*JUP*), membrane curvature protein kinase C and casein kinase substrate in neurons 2 (*PACSIN2*), caveolin 2 (*CAV2*), filamin A (*FLNA*), and spectrin alpha, non-erythrocytic 1 (*SPTAN1*)—were also significantly inversely correlated with hepatic steatosis. Interestingly, however, expression of only some of these genes was significantly downregulated post-lipid challenge, suggesting complex dynamics between the cell cytoskeleton and LD homeostasis.

Other prominent cellular functions emerging from the inversely correlated genes include impairments in endosomal trafficking, autophagy, and lysosomal degradation, all of which can exacerbate steatosis. The expression of NPC intracellular cholesterol transporter 1 (*NPC1*), which plays an essential role in the intracellular trafficking of cholesterol from late endosomes and lysosomes to the plasma membrane and endoplasmic reticulum, and LDLR-related protein 10 (*LRP10*), which is involved in receptor-mediated endocytosis and hepatic uptake/trafficking of lipoprotein ligands, was inversely correlated with hepatic steatosis measures suggesting potential role of impaired endolysosomal trafficking in hepatic steatosis risk [64,65]. Similarly, the expression of genes encoding early endosome fusion regulator (RAB5C, member RAS oncogene family [*RAB5C*]), recycling endosome effector (RAB11 family interacting protein 1 [*RAB11FIP1*]), membrane-tubulating proteins (EH domain containing 1 and 4 [*EHD1*, and *EHD4*]), and lysosomal hydrolytic enzymes and trafficking machinery (lysosome associated membrane protein 1 [*LAMP1*], glucocerebrosidase [*GBA*], iduronate 2-sulfatase [*IDS*], mannosidase alpha class 2B member 2 [*MAN2B2*], prosaposin [*PSAP*], and VPS41 subunit of HOPS complex [*VPS41*]) was also inversely correlated with hepatic steatosis measures across both baseline and post-lipid challenge. Lysosomal degradation pathways, working in tandem with chaperone-mediated autophagy (facilitated by heat shock protein family A (Hsp70) member 8 [*HSPA8*]) and selective autophagic receptors like sequestosome 1 (*SQSTM1*), are vital for the turnover of intracellular organelles and stored lipids. Taken together, these gene profiles suggest that the downregulation of lysosomal clearance capacity and autophagic flux accelerates neutral lipid storage and exacerbates hepatocellular steatosis [66].

In addition to these cellular structural components adversely impacting lipid homeostasis in hepatocytes, the inversely correlated genes also showed enrichment of genes associated with *TGFB2*/*TGFBR2* and *STAT3* pathways, anti-apoptotic responses, ubiquitin-mediated protein degradation, and protein folding/refolding chaperones, suggesting loss of survival and metabolic resilience with hepatic steatosis [67,68]. Other significant genes whose expression was inversely correlated with hepatic steatosis included long-chain acyl-CoA dehydrogenase (*ACADL*), a key enzyme for mitochondrial β-oxidation of long-chain fatty acids, and lipid remodeling and signaling enzymes such as diacylglycerol kinase eta (*DGKH*), glycerophosphodiester phosphodiesterase 1 (*GDE1*), phospholipase C beta 1 (*PLCB1*), and phospholipase D family member 5 (*PLD5*), suggestive of widespread alteration in membrane phospholipid composition and second-messenger lipid signaling that may be associated with hepatic steatosis [69,70,71,72,73].

## 5. Conclusions

Our work demonstrated an epidemiological-scale use of an iPSC-derived hepatocyte model to identify the transcriptomic determinants of MASLD-associated hepatic steatosis risk. We showed hepatic steatosis (neutral lipid) measures in iPSC-derived hepatocytes were significantly heritable, and we identified a comprehensive list of genes whose expression showed a statistically significant association with cellular hepatic steatosis measures both at baseline and post-lipid challenge. Functional annotations and pathway enrichment analysis of these genes implicate a vast array of hepatocellular functions and outline an overall transcriptomic architecture of MASLD-associated steatosis risk in Mexican Americans. The genes whose expression was positively correlated with hepatic steatosis measures suggest a direct role of processes involved in FA and cholesterol uptake, DNL, and carbohydrate shunts in increased hepatic steatosis, while cellular stress and simultaneous activation of FA-oxidation and ketogenesis indicate an associated state of high-turnover metabolism. The cohort of genes whose expression was inversely correlated with hepatic steatosis measures suggests a significant role of the cellular cytoskeleton, hepatocytes’ epithelial integrity, and endosomal and autophagic clearance machinery in hepatic steatosis risk. The gene list also suggests a reduced metabolic resilience in steatotic hepatocytes. While these in vitro findings await functional validation and validation against organismal phenotypes, this scalable human model provides a high-resolution genetic map of MASLD-associated steatosis risk for targeted therapeutic discovery.

## Data Availability

The mRNA sequence data generated from all 193 iPSC-derived hepatocyte cultures at baseline (HepC) and post-lipid challenges (HepFA) were submitted to the Gene Expression Omnibus (GEO) archive and are available under the accession number GSE342568.

## Supplementary Materials

The following supporting information can be downloaded at https://www.mdpi.com/article/10.3390/cells15171592/s1: Supplementary Table S1: Genes whose expression was significantly associated with baseline hepatic steatosis measures. Supplementary Table S2: Functional annotation of key genes whose expression was directly correlated with baseline steatosis measures. Supplementary Table S3: Functional annotation of key genes whose baseline expression was inversely correlated with baseline hepatic steatosis measures. Supplementary Table S4: Genes whose expression was significantly associated with post-lipid-challenge hepatic steatosis measures.

## References

[1] Miller D.M., McCauley K.F., Dunham-Snary K.J. (2025). Metabolic Dysfunction-Associated Steatotic Liver Disease (MASLD): Mechanisms, Clinical Implications and Therapeutic Advances. Endocrinol. Diabetes Metab..

[2] Rinella M.E., Lazarus J.V., Ratziu V., Francque S.M., Sanyal A.J., Kanwal F., Romero D., Abdelmalek M.F., Anstee Q.M., Arab J.P. (2023). A multisociety Delphi consensus statement on new fatty liver disease nomenclature. J. Hepatol..

[3] Cumpian N.A., Gutierrez J.A., Wu W., Saab S. (2025). Targeting MASLD and MASH in the US Hispanic/Latino Population: A Review. JAMA Intern. Med..

[4] Rich N.E., Oji S., Mufti A.R., Browning J.D., Parikh N.D., Odewole M., Mayo H., Singal A.G. (2018). Racial and Ethnic Disparities in Nonalcoholic Fatty Liver Disease Prevalence, Severity, and Outcomes in the United States: A Systematic Review and Meta-analysis. Clin. Gastroenterol. Hepatol..

[5] Gill C., Vatcheva K.P., Pan J.J., Smulevitz B., McPherson D.D., Fallon M., McCormick J.B., Fisher-Hoch S.P., Laing S.T. (2017). Frequency of Nonalcoholic Fatty Liver Disease and Subclinical Atherosclerosis Among Young Mexican Americans. Am. J. Cardiol..

[6] Schulze R.J., Schott M.B., Casey C.A., Tuma P.L., McNiven M.A. (2019). The cell biology of the hepatocyte: A membrane trafficking machine. J. Cell Biol..

[7] Choi S.H., Ginsberg H.N. (2011). Increased very low density lipoprotein (VLDL) secretion, hepatic steatosis, and insulin resistance. Trends Endocrinol. Metab..

[8] Mahdessian H., Taxiarchis A., Popov S., Silveira A., Franco-Cereceda A., Hamsten A., Eriksson P., van’t Hooft F. (2014). TM6SF2 is a regulator of liver fat metabolism influencing triglyceride secretion and hepatic lipid droplet content. Proc. Natl. Acad. Sci. USA.

[9] van Zwol W., van de Sluis B., Ginsberg H.N., Kuivenhoven J.A. (2024). VLDL Biogenesis and Secretion: It Takes a Village. Circ. Res..

[10] Mashek D.G. (2021). Hepatic lipid droplets: A balancing act between energy storage and metabolic dysfunction in NAFLD. Mol. Metab..

[11] Abul-Husn N.S., Cheng X., Li A.H., Xin Y., Schurmann C., Stevis P., Liu Y., Kozlitina J., Stender S., Wood G.C. (2018). A Protein-Truncating HSD17B13 Variant and Protection from Chronic Liver Disease. N. Engl. J. Med..

[12] Faulkner C.S., White C.M., Shah V.H., Jophlin L.L. (2020). A single nucleotide polymorphism of PLIN2 is associated with nonalcoholic steatohepatitis and causes phenotypic changes in hepatocyte lipid droplets: A pilot study. Biochim. Biophys. Acta Mol. Cell Biol. Lipids.

[13] Romeo S., Kozlitina J., Xing C., Pertsemlidis A., Cox D., Pennacchio L.A., Boerwinkle E., Cohen J.C., Hobbs H.H. (2008). Genetic variation in PNPLA3 confers susceptibility to nonalcoholic fatty liver disease. Nat. Genet..

[14] Rouhani F., Kumasaka N., de Brito M.C., Bradley A., Vallier L., Gaffney D. (2014). Genetic background drives transcriptional variation in human induced pluripotent stem cells. PLoS Genet..

[15] Kumar S., Curran J.E., Espinosa E.C., Glahn D.C., Blangero J. (2020). Highly efficient induced pluripotent stem cell reprogramming of cryopreserved lymphoblastoid cell lines. J. Biol. Methods.

[16] Kumar S., Curran J.E., Glahn D.C., Blangero J. (2016). Utility of Lymphoblastoid Cell Lines for Induced Pluripotent Stem Cell Generation. Stem Cells Int..

[17] Kumar S., Blangero J., Curran J.E. (2018). Induced Pluripotent Stem Cells in Disease Modeling and Gene Identification. Methods Mol. Biol..

[18] MacCluer J.W., Stern M.P., Almasy L., Atwood L.A., Blangero J., Comuzzie A.G., Dyke B., Haffner S.M., Henkel R.D., Hixson J.E. (1999). Genetics of atherosclerosis risk factors in Mexican Americans. Nutr. Rev..

[19] Ramstetter M.D., Shenoy S.A., Dyer T.D., Lehman D.M., Curran J.E., Duggirala R., Blangero J., Mezey J.G., Williams A.L. (2018). Inferring Identical-by-Descent Sharing of Sample Ancestors Promotes High-Resolution Relative Detection. Am. J. Hum. Genet..

[20] Kumar S., Curran J.E., Williams-Blangero S., Blangero J. (2021). Efficient Generation of Functional Hepatocytes from Human Induced Pluripotent Stem Cells for Disease Modeling and Disease Gene Discovery. Methods Mol. Biol..

[21] Almasy L., Blangero J. (1998). Multipoint quantitative-trait linkage analysis in general pedigrees. Am. J. Hum. Genet..

[22] Dunn O.J. (1961). Multiple Comparisons among Means. J. Am. Stat. Assoc..

[23] The Gene Ontology Consortium (2026). The Gene Ontology knowledgebase in 2026. Nucleic Acids Res..

[24] Ashburner M., Ball C.A., Blake J.A., Botstein D., Butler H., Cherry J.M., Davis A.P., Dolinski K., Dwight S.S., Eppig J.T. (2000). Gene ontology: Tool for the unification of biology. The Gene Ontology Consortium. Nat. Genet..

[25] Kanehisa M. (2019). Toward understanding the origin and evolution of cellular organisms. Protein Sci..

[26] Kanehisa M., Furumichi M., Sato Y., Matsuura Y., Ishiguro-Watanabe M. (2025). KEGG: Biological systems database as a model of the real world. Nucleic Acids Res..

[27] Kanehisa M., Goto S. (2000). KEGG: Kyoto encyclopedia of genes and genomes. Nucleic Acids Res..

[28] Chen E.Y., Tan C.M., Kou Y., Duan Q., Wang Z., Meirelles G.V., Clark N.R., Ma’ayan A. (2013). Enrichr: Interactive and collaborative HTML5 gene list enrichment analysis tool. BMC Bioinform..

[29] Ge S.X., Jung D., Yao R. (2020). ShinyGO: A graphical gene-set enrichment tool for animals and plants. Bioinformatics.

[30] Kramer A., Green J., Pollard J., Tugendreich S. (2014). Causal analysis approaches in Ingenuity Pathway Analysis. Bioinformatics.

[31] Manusov E.G., Diego V.P., Almeida M., Galan J.A., Bala A.A., Arriaga M.A., Garcia-Rodriguez N.S., Hernandez R., Kumar S., Blangero B., Ju-Seop K. (2024). Gene-Environment Interactions in Non-alcoholic Fatty Liver Disease: Insights from Mexican American Populations. A Comprehensive Guide to Non-Alcoholic Fatty Liver Disease.

[32] Perez V.M., Gabell J., Behrens M., Wase N., DiRusso C.C., Black P.N. (2020). Deletion of fatty acid transport protein 2 (FATP2) in the mouse liver changes the metabolic landscape by increasing the expression of PPARalpha-regulated genes. J. Biol. Chem..

[33] Wang G., Bonkovsky H.L., de Lemos A., Burczynski F.J. (2015). Recent insights into the biological functions of liver fatty acid binding protein 1. J. Lipid Res..

[34] Soccio R.E., Adams R.M., Romanowski M.J., Sehayek E., Burley S.K., Breslow J.L. (2002). The cholesterol-regulated StarD4 gene encodes a StAR-related lipid transfer protein with two closely related homologues, StarD5 and StarD6. Proc. Natl. Acad. Sci. USA.

[35] Abdul-Wahed A., Guilmeau S., Postic C. (2017). Sweet Sixteenth for ChREBP: Established Roles and Future Goals. Cell Metab..

[36] Matsuzaka T., Shimano H. (2009). Elovl6: A new player in fatty acid metabolism and insulin sensitivity. J. Mol. Med..

[37] Parini P., Davis M., Lada A.T., Erickson S.K., Wright T.L., Gustafsson U., Sahlin S., Einarsson C., Eriksson M., Angelin B. (2004). ACAT2 is localized to hepatocytes and is the major cholesterol-esterifying enzyme in human liver. Circulation.

[38] Wetterau J.R., Aggerbeck L.P., Bouma M.E., Eisenberg C., Munck A., Hermier M., Schmitz J., Gay G., Rader D.J., Gregg R.E. (1992). Absence of microsomal triglyceride transfer protein in individuals with abetalipoproteinemia. Science.

[39] Wang Y., Yu W., Li S., Guo D., He J., Wang Y. (2022). Acetyl-CoA Carboxylases and Diseases. Front. Oncol..

[40] Eslam M., Newsome P.N., Sarin S.K., Anstee Q.M., Targher G., Romero-Gomez M., Zelber-Sagi S., Wai-Sun Wong V., Dufour J.F., Schattenberg J.M. (2020). A new definition for metabolic dysfunction-associated fatty liver disease: An international expert consensus statement. J. Hepatol..

[41] Berk P.D., Stump D.D. (1999). Mechanisms of cellular uptake of long chain free fatty acids. Mol. Cell. Biochem..

[42] Zhang M., Bai X., Du Q., Xu J., Wang D., Chen L., Dong K., Chen Z., Yang J. (2023). The Different Mechanisms of Lipid Accumulation in Hepatocytes Induced by Oleic Acid/Palmitic Acid and High-Fat Diet. Molecules.

[43] Jia L., Betters J.L., Yu L. (2011). Niemann-pick C1-like 1 (NPC1L1) protein in intestinal and hepatic cholesterol transport. Annu. Rev. Physiol..

[44] Yu L., Bharadwaj S., Brown J.M., Ma Y., Du W., Davis M.A., Michaely P., Liu P., Willingham M.C., Rudel L.L. (2006). Cholesterol-regulated translocation of NPC1L1 to the cell surface facilitates free cholesterol uptake. J. Biol. Chem..

[45] Riad A., Zeng C., Weng C.C., Winters H., Xu K., Makvandi M., Metz T., Carlin S., Mach R.H. (2018). Sigma-2 Receptor/TMEM97 and PGRMC-1 Increase the Rate of Internalization of LDL by LDL Receptor through the Formation of a Ternary Complex. Sci. Rep..

[46] Wang J.Q., Li L.L., Hu A., Deng G., Wei J., Li Y.F., Liu Y.B., Lu X.Y., Qiu Z.P., Shi X.J. (2022). Inhibition of ASGR1 decreases lipid levels by promoting cholesterol excretion. Nature.

[47] Sandhu J., Li S., Fairall L., Pfisterer S.G., Gurnett J.E., Xiao X., Weston T.A., Vashi D., Ferrari A., Orozco J.L. (2018). Aster Proteins Facilitate Nonvesicular Plasma Membrane to ER Cholesterol Transport in Mammalian Cells. Cell.

[48] Mesmin B., Pipalia N.H., Lund F.W., Ramlall T.F., Sokolov A., Eliezer D., Maxfield F.R. (2011). STARD4 abundance regulates sterol transport and sensing. Mol. Biol. Cell.

[49] Donnelly K.L., Margosian M.R., Sheth S.S., Lusis A.J., Parks E.J. (2004). Increased lipogenesis and fatty acid reesterification contribute to hepatic triacylglycerol stores in hyperlipidemic Txnip-/- mice. J. Nutr..

[50] Smith G.I., Shankaran M., Yoshino M., Schweitzer G.G., Chondronikola M., Beals J.W., Okunade A.L., Patterson B.W., Nyangau E., Field T. (2020). Insulin resistance drives hepatic de novo lipogenesis in nonalcoholic fatty liver disease. J. Clin. Investig..

[51] Truong X.T., Lee D.H. (2025). Hepatic Insulin Resistance and Steatosis in Metabolic Dysfunction-Associated Steatotic Liver Disease: New Insights into Mechanisms and Clinical Implications. Diabetes Metab. J..

[52] Iizuka K. (2017). The Role of Carbohydrate Response Element Binding Protein in Intestinal and Hepatic Fructose Metabolism. Nutrients.

[53] Ipsen D.H., Lykkesfeldt J., Tveden-Nyborg P. (2018). Molecular mechanisms of hepatic lipid accumulation in non-alcoholic fatty liver disease. Cell. Mol. Life Sci..

[54] Koo S.H. (2013). Nonalcoholic fatty liver disease: Molecular mechanisms for the hepatic steatosis. Clin. Mol. Hepatol..

[55] Kazierad D.J., Chidsey K., Somayaji V.R., Bergman A.J., Birnbaum M.J., Calle R.A. (2021). Inhibition of ketohexokinase in adults with NAFLD reduces liver fat and inflammatory markers: A randomized phase 2 trial. Med.

[56] Li F., Huangyang P., Burrows M., Guo K., Riscal R., Godfrey J., Lee K.E., Lin N., Lee P., Blair I.A. (2020). FBP1 loss disrupts liver metabolism and promotes tumorigenesis through a hepatic stellate cell senescence secretome. Nat. Cell Biol..

[57] Zhu Z., Zhang X., Pan Q., Zhang L., Chai J. (2023). In-depth analysis of de novo lipogenesis in non-alcoholic fatty liver disease: Mechanism and pharmacological interventions. Liver Res..

[58] Loneker A.E., Alisafaei F., Kant A., Li D., Janmey P.A., Shenoy V.B., Wells R.G. (2023). Lipid droplets are intracellular mechanical stressors that impair hepatocyte function. Proc. Natl. Acad. Sci. USA.

[59] Fan L., Gokaltun A., Maggipinto S., Kitagawa Y., Martyn J., Yeh H., Uygun B.E., Yarmush M.L., Usta O.B. (2023). Alterations in Cytoskeleton and Mitochondria in the Development and Reversal of Steatosis in Human Hepatocytes. Cell. Mol. Gastroenterol. Hepatol..

[60] Jin Y., Ren Z., Tan Y., Zhao P., Wu J. (2021). Motility Plays an Important Role in the Lifetime of Mammalian Lipid Droplets. Int. J. Mol. Sci..

[61] Kilwein M.D., Welte M.A. (2019). Lipid droplet motility and organelle contacts. Contact.

[62] Blot C., Bertrand M., Siponen M., Li-Beisson Y. (2026). Lipid droplets on the move: Remodeling, trafficking, and interaction with other organelles. J. Exp. Bot..

[63] Liang Y., Zhu Z., Lu Y., Ma C., Li J., Yu K., Wu J., Che X., Liu X., Huang X. (2025). Cytoskeleton regulates lipid droplet fusion and lipid storage by controlling lipid droplet movement. Biochim. Biophys. Acta Mol. Cell Biol. Lipids.

[64] Yu X.H., Jiang N., Yao P.B., Zheng X.L., Cayabyab F.S., Tang C.K. (2014). NPC1, intracellular cholesterol trafficking and atherosclerosis. Clin. Chim. Acta.

[65] Pohlkamp T., Wasser C.R., Herz J. (2017). Functional Roles of the Interaction of APP and Lipoprotein Receptors. Front. Mol. Neurosci..

[66] Qian H., Chao X., Williams J., Fulte S., Li T., Yang L., Ding W.X. (2021). Autophagy in liver diseases: A review. Mol. Asp. Med..

[67] Duan L., Chang Y., Dai J., Lu H., Zhao W., Shen Y., Lin J., Cai X. (2025). Lipid metabolism orchestrates liver regeneration: An integrated metabolic network. J. Transl. Med..

[68] Fabregat I., Moreno-Caceres J., Sanchez A., Dooley S., Dewidar B., Giannelli G., Ten Dijke P., Consortium I.-L. (2016). TGF-beta signalling and liver disease. FEBS J..

[69] Lefort C., Roumain M., Van Hul M., Rastelli M., Manco R., Leclercq I., Delzenne N.M., Marzo V.D., Flamand N., Luquet S. (2020). Hepatic NAPE-PLD Is a Key Regulator of Liver Lipid Metabolism. Cells.

[70] Zheng B., Berrie C.P., Corda D., Farquhar M.G. (2003). GDE1/MIR16 is a glycerophosphoinositol phosphodiesterase regulated by stimulation of G protein-coupled receptors. Proc. Natl. Acad. Sci. USA.

[71] Wang H., Zhao Y., Pan Y., Yang A., Li C., Wang S., Dong Z., Li M., Wang S., Zhang Z. (2023). Inhibition of phospholipase D1 ameliorates hepatocyte steatosis and non-alcoholic fatty liver disease. JHEP Rep..

[72] Zhang J., Zhao J., Zheng X., Cai K., Mao Q., Xia H. (2018). Establishment of a novel hepatic steatosis cell model by Cas9/sgRNA-mediated DGKtheta gene knockout. Mol. Med. Rep..

[73] Liu Y., Yang Z., Zhou X., Li Z., Hideki N. (2024). Diacylglycerol Kinases and Its Role in Lipid Metabolism and Related Diseases. Int. J. Mol. Sci..

