# Supplementary material for: Transcriptomic Architecture of Metabolic Dysfunction-Associated Steatotic Liver Disease (MASLD) Risk in Mexican Americans
Source: Cells. 2026 Sep 1;15(17):1592. doi: 10.3390/cells15171592 (PMC13565466; doi:10.3390/cells15171592)
Supplement: Supplementary file 1 [file cells-15-01592-s001.zip › Supplementary Table S4.pdf]

**Supplementary Table S4**: Genes whose expression was significantly associated with post-lipid challenge hepatic steatosis measures.

| S. No. | Entrez ID | Gene Symbol | ***β*** | ***β*** **($\chi^2$)** | **Bonferroni-corrected *p*-value** |
|---|---|---|---|---|---|
| 1 | 4088 | *SMAD3* | −0.487 | 139.933 | 4.88E−28 |
| 2 | 10893 | *MMP24* | −0.457 | 130.756 | 4.96E−26 |
| 3 | 27242 | *TNFRSF21* | −0.441 | 123.217 | 2.22E−24 |
| 4 | 9948 | *WDR1* | −0.462 | 118.944 | 1.91E−23 |
| 5 | 29995 | *LMCD1* | −0.435 | 107.955 | 4.87E−21 |
| 6 | 55915 | *LANCL2* | −0.427 | 106.281 | 1.13E−20 |
| 7 | 8654 | *PDE5A* | −0.418 | 103.811 | 3.94E−20 |
| 8 | 25816 | *TNFAIP8* | −0.435 | 102.775 | 6.65E−20 |
| 9 | 1286 | *COL4A4* | −0.443 | 102.209 | 8.86E−20 |
| 10 | 51062 | *ATL1* | −0.437 | 101.105 | 1.55E−19 |
| 11 | 6397 | *SEC14L1* | −0.408 | 98.189 | 6.74E−19 |
| 12 | 3927 | *LASP1* | −0.394 | 96.405 | 1.66E−18 |
| 13 | 3797 | *KIF3C* | −0.409 | 94.770 | 3.79E−18 |
| 14 | 824 | *CAPN2* | −0.429 | 93.900 | 5.88E−18 |
| 15 | 11098 | *PRSS23* | −0.405 | 93.045 | 9.06E−18 |
| 16 | 2168 | *FABP1* | 0.403 | 91.776 | 1.72E−17 |
| 17 | 9052 | *GPRC5A* | −0.397 | 91.702 | 1.79E−17 |
| 18 | 7286 | *TUFT1* | −0.406 | 89.708 | 4.89E−17 |
| 19 | 7056 | *THBD* | −0.385 | 89.636 | 5.07E−17 |
| 20 | 30844 | *EHD4* | −0.402 | 88.739 | 7.99E−17 |
| 21 | 622 | *BDH1* | 0.396 | 87.667 | 1.37E−16 |
| 22 | 7360 | *UGP2* | −0.482 | 87.413 | 1.56E−16 |
| 23 | 8435 | *SOAT2* | 0.387 | 86.937 | 1.99E−16 |
| 24 | 5770 | *PTPN1* | −0.418 | 86.414 | 2.59E−16 |
| 25 | 3423 | *IDS* | −0.407 | 85.702 | 3.71E−16 |
| 26 | 8128 | *ST8SIA2* | −0.384 | 84.720 | 6.09E−16 |
| 27 | 2823 | *GPM6A* | −0.391 | 84.636 | 6.36E−16 |
| 28 | 51208 | *CLDN18* | 0.396 | 83.815 | 9.63E−16 |
| 29 | 6578 | *SLCO2A1* | −0.390 | 83.358 | 1.21E−15 |
| 30 | 10231 | *RCAN2* | −0.379 | 83.183 | 1.33E−15 |
| 31 | 55770 | *EXOC2* | −0.453 | 82.989 | 1.46E−15 |
| 32 | 8407 | *TAGLN2* | −0.415 | 82.938 | 1.50E−15 |
| 33 | 7126 | *TNFAIP1* | −0.399 | 82.899 | 1.53E−15 |
| 34 | 3694 | *ITGB6* | −0.408 | 82.677 | 1.71E−15 |
| 35 | 386617 | *KCTD8* | −0.383 | 82.253 | 2.12E−15 |
| 36 | 3918 | *LAMC2* | −0.450 | 82.229 | 2.15E−15 |
| 37 | 26136 | *TES* | −0.392 | 82.162 | 2.22E−15 |
| 38 | 200150 | *PLD5* | −0.386 | 81.106 | 3.79E−15 |
| 39 | 5648 | *MASP1* | 0.376 | 80.748 | 4.55E−15 |
| 40 | 283635 | *FAM177A1* | −0.385 | 80.668 | 4.73E−15 |
| 41 | 9991 | *PTBP3* | −0.501 | 80.192 | 6.02E−15 |
| 42 | 374378 | *GALNT18* | −0.396 | 79.965 | 6.75E−15 |
| 43 | 338645 | *LUZP2* | −0.380 | 79.699 | 7.73E−15 |
| 44 | 3294 | *HSD17B2* | 0.404 | 79.455 | 8.74E−15 |
| 45 | 23233 | *EXOC6B* | −0.445 | 79.198 | 9.96E−15 |
| 46 | 1837 | *DTNA* | −0.378 | 78.927 | 1.14E−14 |
| 47 | 283209 | *PGM2L1* | −0.442 | 78.491 | 1.42E−14 |
| 48 | 56675 | *NRIP3* | −0.401 | 78.223 | 1.63E−14 |
| 49 | 9124 | *PDLIM1* | −0.366 | 77.188 | 2.75E−14 |

| | | | | | |
|---|---|---|---|---|---|
| 50 | 9240 | *PNMA1* | −0.396 | 77.091 | 2.89E−14 |
| 51 | 304 | *ANXA2P2* | −0.378 | 76.902 | 3.18E−14 |
| 52 | 2051 | *EPHB6* | −0.417 | 76.523 | 3.86E−14 |
| 53 | 25837 | *RAB26* | 0.464 | 76.498 | 3.91E−14 |
| 54 | 4645 | *MYO5B* | −0.428 | 76.449 | 4.01E−14 |
| 55 | 3983 | *ABLIM1* | −0.417 | 76.032 | 4.95E−14 |
| 56 | 3985 | *LIMK2* | −0.416 | 75.764 | 5.67E−14 |
| 57 | 4013 | *VWA5A* | −0.436 | 75.564 | 6.27E−14 |
| 58 | 25840 | *METTL7A* | 0.364 | 75.494 | 6.50E−14 |
| 59 | 26355 | *FAM162A* | 0.366 | 75.387 | 6.86E−14 |
| 60 | 23603 | *CORO1C* | −0.465 | 75.278 | 7.25E−14 |
| 61 | 71 | *ACTG1* | −0.383 | 75.116 | 7.87E−14 |
| 62 | 285761 | *DCBLD1* | −0.483 | 75.022 | 8.25E−14 |
| 63 | 10602 | *CDC42EP3* | −0.384 | 74.842 | 9.04E−14 |
| 64 | 8291 | *DYSF* | −0.367 | 74.741 | 9.51E−14 |
| 65 | 5638 | *PRRG1* | −0.442 | 74.730 | 9.57E−14 |
| 66 | 79669 | *C3orf52* | −0.400 | 74.632 | 1.01E−13 |
| 67 | 58 | *ACTA1* | −0.388 | 74.573 | 1.04E−13 |
| 68 | 25893 | *TRIM58* | −0.366 | 73.770 | 1.56E−13 |
| 69 | 120114 | *FAT3* | −0.397 | 73.548 | 1.74E−13 |
| 70 | 5912 | *RAP2B* | −0.376 | 73.518 | 1.77E−13 |
| 71 | 54741 | *LEPROT* | −0.442 | 73.154 | 2.13E−13 |
| 72 | 11099 | *PTPN21* | −0.378 | 72.954 | 2.35E−13 |
| 73 | 4773 | *NFATC2* | 0.358 | 72.948 | 2.36E−13 |
| 74 | 23245 | *ASTN2* | −0.369 | 72.665 | 2.72E−13 |
| 75 | 4627 | *MYH9* | −0.363 | 72.384 | 3.14E−13 |
| 76 | 4643 | *MYO1E* | −0.409 | 71.625 | 4.61E−13 |
| 77 | 2053 | *EPHX2* | 0.349 | 71.614 | 4.64E−13 |
| 78 | 84280 | *BTBD10* | −0.416 | 71.538 | 4.82E−13 |
| 79 | 2782 | *GNB1* | −0.402 | 71.493 | 4.93E−13 |
| 80 | 1.02E+08 | *PCAT14* | 0.368 | 71.314 | 5.40E−13 |
| 81 | 64759 | *TNS3* | −0.438 | 71.262 | 5.54E−13 |
| 82 | 11259 | *FILIP1L* | −0.374 | 71.001 | 6.33E−13 |
| 83 | 649 | *BMP1* | −0.378 | 70.825 | 6.92E−13 |
| 84 | 3835 | *KIF22* | 0.383 | 70.780 | 7.08E−13 |
| 85 | 8842 | *PROM1* | −0.458 | 70.695 | 7.39E−13 |
| 86 | 272 | *AMPD3* | −0.391 | 70.584 | 7.82E−13 |
| 87 | 81619 | *TSPAN14* | −0.361 | 70.255 | 9.23E−13 |
| 88 | 1807 | *DPYS* | 0.372 | 70.159 | 9.70E−13 |
| 89 | 57624 | *NYAP2* | −0.373 | 70.110 | 9.94E−13 |
| 90 | 9645 | *MICAL2* | −0.421 | 69.458 | 1.38E−12 |
| 91 | 8218 | *CLTCL1* | −0.357 | 69.446 | 1.39E−12 |
| 92 | 647024 | *C6orf132* | −0.359 | 69.430 | 1.40E−12 |
| 93 | 432 | *ASGR1* | 0.377 | 69.406 | 1.42E−12 |
| 94 | 27319 | *BHLHE22* | −0.354 | 69.361 | 1.45E−12 |
| 95 | 26353 | *HSPB8* | −0.356 | 69.174 | 1.60E−12 |
| 96 | 6876 | *TAGLN* | −0.386 | 69.064 | 1.69E−12 |
| 97 | 54948 | *MRPL16* | 0.378 | 68.933 | 1.81E−12 |
| 98 | 27122 | *DKK3* | −0.347 | 68.825 | 1.91E−12 |
| 99 | 5530 | *PPP3CA* | −0.403 | 68.752 | 1.98E−12 |
| 100 | 126282 | *TNFAIP8L1* | 0.357 | 68.437 | 2.32E−12 |
| 101 | 10841 | *FTCD* | 0.409 | 68.398 | 2.37E−12 |
| 102 | 2746 | *GLUD1* | 0.373 | 68.321 | 2.46E−12 |

| | | | | | |
|---|---|---|---|---|---|
| 103 | 1363 | *CPE* | −0.359 | 68.310 | 2.48E−12 |
| 104 | 1495 | *CTNNA1* | −0.449 | 68.119 | 2.73E−12 |
| 105 | 5225 | *PGC* | 0.354 | 67.900 | 3.05E−12 |
| 106 | 5948 | *RBP2* | 0.353 | 67.845 | 3.14E−12 |
| 107 | 8802 | *SUCLG1* | 0.352 | 67.764 | 3.27E−12 |
| 108 | 651746 | *ANKRD33B* | −0.357 | 67.496 | 3.74E−12 |
| 109 | 5228 | *PGF* | 0.359 | 67.222 | 4.30E−12 |
| 110 | 7532 | *YWHAG* | −0.384 | 67.115 | 4.54E−12 |
| 111 | 3340 | *NDST1* | −0.380 | 67.055 | 4.68E−12 |
| 112 | 50859 | *SPOCK3* | −0.352 | 66.717 | 5.56E−12 |
| 113 | 11075 | *STMN2* | −0.361 | 66.351 | 6.69E−12 |
| 114 | 10487 | *CAP1* | −0.437 | 66.201 | 7.22E−12 |
| 115 | 29881 | *NPC1L1* | 0.365 | 66.200 | 7.22E−12 |
| 116 | 4864 | *NPC1* | −0.400 | 66.141 | 7.44E−12 |
| 117 | 5016 | *OVGP1* | 0.355 | 65.719 | 9.22E−12 |
| 118 | 64420 | *SUSD1* | −0.417 | 65.658 | 9.51E−12 |
| 119 | 115353 | *LRRC42* | −0.387 | 65.556 | 1.00E−11 |
| 120 | 8482 | *SEMA7A* | −0.357 | 65.541 | 1.01E−11 |
| 121 | 59277 | *NTN4* | −0.363 | 65.529 | 1.01E−11 |
| 122 | 79695 | *GALNT12* | 0.350 | 65.438 | 1.06E−11 |
| 123 | 116372 | *LYPD1* | −0.410 | 65.416 | 1.08E−11 |
| 124 | 23176 | *SEPTIN8* | −0.358 | 65.271 | 1.16E−11 |
| 125 | 754 | *PTTG1IP* | −0.374 | 64.834 | 1.44E−11 |
| 126 | 7852 | *CXCR4* | −0.343 | 64.558 | 1.66E−11 |
| 127 | 1030 | *CDKN2B* | −0.352 | 64.545 | 1.67E−11 |
| 128 | 56938 | *ARNTL2* | −0.383 | 64.533 | 1.68E−11 |
| 129 | 9468 | *PCYT1B* | 0.345 | 64.472 | 1.74E−11 |
| 130 | 6920 | *TCEA3* | 0.344 | 64.379 | 1.82E−11 |
| 131 | 10096 | *ACTR3* | −0.419 | 64.352 | 1.84E−11 |
| 132 | 27346 | *TMEM97* | 0.355 | 64.199 | 1.99E−11 |
| 133 | 3398 | *ID2* | 0.387 | 64.173 | 2.02E−11 |
| 134 | 57579 | *FAM135A* | −0.399 | 63.471 | 2.88E−11 |
| 135 | 414241 | *SHLD2P1* | 0.365 | 63.429 | 2.95E−11 |
| 136 | 3097 | *HIVEP2* | −0.401 | 63.419 | 2.96E−11 |
| 137 | 9208 | *LRRFIP1* | −0.407 | 63.297 | 3.15E−11 |
| 138 | 10381 | *TUBB3* | −0.385 | 63.194 | 3.32E−11 |
| 139 | 170954 | *PPP1R18* | −0.383 | 63.120 | 3.45E−11 |
| 140 | 57476 | *GRAMD1B* | 0.345 | 63.036 | 3.60E−11 |
| 141 | 8796 | *SCEL* | −0.360 | 62.823 | 4.01E−11 |
| 142 | 10808 | *HSPH1* | −0.385 | 62.801 | 4.05E−11 |
| 143 | 1889 | *ECE1* | −0.341 | 62.757 | 4.15E−11 |
| 144 | 55349 | *CHDH* | 0.388 | 62.752 | 4.16E−11 |
| 145 | 3875 | *KRT18* | −0.349 | 62.562 | 4.58E−11 |
| 146 | 3856 | *KRT8* | −0.379 | 62.445 | 4.86E−11 |
| 147 | 5330 | *PLCB2* | 0.358 | 62.322 | 5.17E−11 |
| 148 | 80854 | *SETD7* | −0.460 | 62.306 | 5.21E−11 |
| 149 | 81563 | *C1orf21* | 0.354 | 62.276 | 5.29E−11 |
| 150 | 57630 | *SH3RF1* | −0.363 | 62.097 | 5.79E−11 |
| 151 | 33 | *ACADL* | −0.339 | 61.997 | 6.10E−11 |
| 152 | 4747 | *NEFL* | −0.338 | 61.988 | 6.12E−11 |
| 153 | 4678 | *NASP* | 0.348 | 61.793 | 6.76E−11 |
| 154 | 55833 | *UBAP2* | −0.367 | 61.772 | 6.83E−11 |
| 155 | 6774 | *STAT3* | −0.436 | 61.513 | 7.80E−11 |

| | | | | | |
|---|---|---|---|---|---|
| 156 | 25852 | *ARMC8* | −0.446 | 61.449 | 8.05E−11 |
| 157 | 55422 | *ZNF331* | 0.335 | 61.116 | 9.54E−11 |
| 158 | 284111 | *SLC13A5* | 0.336 | 61.082 | 9.71E−11 |
| 159 | 9646 | *CTR9* | −0.470 | 61.044 | 9.89E−11 |
| 160 | 23313 | *KIAA0930* | −0.365 | 60.929 | 1.05E−10 |
| 161 | 5507 | *PPP1R3C* | 0.346 | 60.926 | 1.05E−10 |
| 162 | 6335 | *SCN9A* | −0.343 | 60.867 | 1.08E−10 |
| 163 | 7431 | *VIM* | −0.365 | 60.842 | 1.10E−10 |
| 164 | 9462 | *RASAL2* | −0.371 | 60.782 | 1.13E−10 |
| 165 | 445 | *ASS1* | 0.345 | 60.699 | 1.18E−10 |
| 166 | 3673 | *ITGA2* | −0.448 | 60.664 | 1.20E−10 |
| 167 | 64432 | *MRPS25* | 0.339 | 60.562 | 1.26E−10 |
| 168 | 23236 | *PLCB1* | −0.387 | 60.417 | 1.36E−10 |
| 169 | 4688 | *NCF2* | −0.336 | 60.210 | 1.51E−10 |
| 170 | 11223 | *MST1L* | 0.338 | 60.182 | 1.53E−10 |
| 171 | 123920 | *CMTM3* | −0.441 | 60.163 | 1.55E−10 |
| 172 | 9989 | *PPP4R1* | −0.389 | 60.147 | 1.56E−10 |
| 173 | 79574 | *EPS8L3* | 0.348 | 59.933 | 1.74E−10 |
| 174 | 898 | *CCNE1* | 0.337 | 59.882 | 1.79E−10 |
| 175 | 1285 | *COL4A3* | −0.337 | 59.877 | 1.79E−10 |
| 176 | 7732 | *RNF112* | 0.352 | 59.834 | 1.83E−10 |
| 177 | 55120 | *FANCL* | 0.342 | 59.770 | 1.89E−10 |
| 178 | 645369 | *TMEM200C* | −0.467 | 59.554 | 2.11E−10 |
| 179 | 144132 | *DNHD1* | 0.388 | 59.402 | 2.28E−10 |
| 180 | 9097 | *USP14* | −0.393 | 59.342 | 2.35E−10 |
| 181 | 79649 | *MAP7D3* | −0.349 | 59.118 | 2.63E−10 |
| 182 | 6249 | *CLIP1* | −0.436 | 58.961 | 2.85E−10 |
| 183 | 157922 | *CAMSAP1* | −0.381 | 58.955 | 2.86E−10 |
| 184 | 367 | *AR* | −0.338 | 58.889 | 2.96E−10 |
| 185 | 170690 | *ADAMTS16* | −0.379 | 58.777 | 3.13E−10 |
| 186 | 9537 | *TP53I11* | −0.370 | 58.612 | 3.40E−10 |
| 187 | 80307 | *FER1L4* | 0.352 | 58.456 | 3.69E−10 |
| 188 | 57194 | *ATP10A* | −0.331 | 58.429 | 3.74E−10 |
| 189 | 2861 | *GPR37* | −0.337 | 58.398 | 3.79E−10 |
| 190 | 2131 | *EXT1* | −0.351 | 58.397 | 3.80E−10 |
| 191 | 1004 | *CDH6* | −0.329 | 58.386 | 3.82E−10 |
| 192 | 9766 | *SUSD6* | −0.361 | 58.332 | 3.93E−10 |
| 193 | 4299 | *AFF1* | −0.472 | 58.200 | 4.20E−10 |
| 194 | 334 | *APLP2* | −0.356 | 58.144 | 4.32E−10 |
| 195 | 79837 | *PIP4K2C* | −0.359 | 57.870 | 4.96E−10 |
| 196 | 51203 | *NUSAP1* | 0.338 | 57.712 | 5.38E−10 |
| 197 | 8682 | *PEA15* | −0.327 | 57.516 | 5.94E−10 |
| 198 | 27072 | *VPS41* | −0.402 | 57.453 | 6.14E−10 |
| 199 | 23108 | *RAP1GAP2* | −0.335 | 57.447 | 6.15E−10 |
| 200 | 127845 | *GOLT1A* | 0.337 | 57.415 | 6.25E−10 |
| 201 | 9528 | *TMEM59* | −0.379 | 57.314 | 6.59E−10 |
| 202 | 9697 | *TRAM2* | −0.405 | 57.297 | 6.64E−10 |
| 203 | 1809 | *DPYSL3* | −0.341 | 57.259 | 6.77E−10 |
| 204 | 9620 | *CELSR1* | −0.336 | 57.215 | 6.92E−10 |
| 205 | 23276 | *KLHL18* | −0.335 | 57.043 | 7.56E−10 |
| 206 | 57182 | *ANKRD50* | −0.437 | 57.025 | 7.63E−10 |
| 207 | 54843 | *SYTL2* | −0.343 | 57.022 | 7.64E−10 |
| 208 | 23286 | *WWC1* | −0.345 | 56.997 | 7.74E−10 |

| | | | | | |
|---|---|---|---|---|---|
| 209 | 552 | *AVPR1A* | −0.381 | 56.960 | 7.88E−10 |
| 210 | 25788 | *RAD54B* | 0.347 | 56.899 | 8.13E−10 |
| 211 | 2941 | *GSTA4* | 0.332 | 56.896 | 8.14E−10 |
| 212 | 389158 | *PLSCR5* | 0.325 | 56.802 | 8.54E−10 |
| 213 | 54798 | *DCHS2* | −0.326 | 56.793 | 8.58E−10 |
| 214 | 202018 | *TAPT1* | −0.358 | 56.787 | 8.61E−10 |
| 215 | 30011 | *SH3KBP1* | −0.328 | 56.753 | 8.76E−10 |
| 216 | 5018 | *OXA1L* | 0.404 | 56.563 | 9.65E−10 |
| 217 | 23670 | *CEMIP2* | −0.429 | 56.542 | 9.75E−10 |
| 218 | 9886 | *RHOBTB1* | −0.398 | 56.478 | 1.01E−09 |
| 219 | 92521 | *SPECC1* | −0.328 | 56.374 | 1.06E−09 |
| 220 | 10890 | *RAB10* | −0.360 | 56.314 | 1.09E−09 |
| 221 | 8497 | *PPFIA4* | 0.345 | 56.252 | 1.13E−09 |
| 222 | 57591 | *MRTFA* | −0.392 | 56.229 | 1.14E−09 |
| 223 | 302 | *ANXA2* | −0.327 | 56.162 | 1.18E−09 |
| 224 | 58489 | *ABHD17C* | −0.353 | 56.156 | 1.19E−09 |
| 225 | 323 | *APBB2* | −0.356 | 56.094 | 1.22E−09 |
| 226 | 24145 | *PANX1* | −0.409 | 56.088 | 1.23E−09 |
| 227 | 60312 | *AFAP1* | −0.340 | 56.055 | 1.25E−09 |
| 228 | 11275 | *KLHL2* | −0.395 | 56.000 | 1.28E−09 |
| 229 | 23446 | *SLC44A1* | 0.430 | 55.994 | 1.29E−09 |
| 230 | 1.03E+08 | *ABALON* | −0.360 | 55.986 | 1.29E−09 |
| 231 | 4790 | *NFKB1* | −0.429 | 55.732 | 1.47E−09 |
| 232 | 415 | *ARSL* | 0.329 | 55.682 | 1.51E−09 |
| 233 | 7204 | *TRIO* | −0.326 | 55.642 | 1.54E−09 |
| 234 | 3853 | *KRT6A* | −0.344 | 55.530 | 1.63E−09 |
| 235 | 9369 | *NRXN3* | −0.348 | 55.484 | 1.67E−09 |
| 236 | 79071 | *ELOVL6* | 0.335 | 55.379 | 1.76E−09 |
| 237 | 1268 | *CNR1* | −0.316 | 55.195 | 1.93E−09 |
| 238 | 9690 | *UBE3C* | −0.433 | 55.167 | 1.96E−09 |
| 239 | 10892 | *MALT1* | −0.323 | 55.101 | 2.03E−09 |
| 240 | 26227 | *PHGDH* | 0.321 | 55.064 | 2.07E−09 |
| 241 | 6288 | *SAA1* | 0.331 | 54.937 | 2.21E−09 |
| 242 | 26509 | *MYOF* | −0.374 | 54.621 | 2.59E−09 |
| 243 | 6470 | *SHMT1* | 0.323 | 54.611 | 2.60E−09 |
| 244 | 6867 | *TACC1* | −0.386 | 54.531 | 2.71E−09 |
| 245 | 139065 | *SLITRK4* | −0.321 | 54.484 | 2.78E−09 |
| 246 | 4615 | *MYD88* | −0.328 | 54.446 | 2.83E−09 |
| 247 | 55741 | *EDEM2* | −0.320 | 54.398 | 2.90E−09 |
| 248 | 5793 | *PTPRG* | −0.333 | 54.270 | 3.10E−09 |
| 249 | 324 | *APC* | −0.385 | 54.051 | 3.46E−09 |
| 250 | 7342 | *UBP1* | −0.409 | 53.899 | 3.74E−09 |
| 251 | 91860 | *CALML4* | 0.346 | 53.872 | 3.79E−09 |
| 252 | 59342 | *SCPEP1* | 0.320 | 53.841 | 3.85E−09 |
| 253 | 4286 | *MITF* | −0.382 | 53.835 | 3.87E−09 |
| 254 | 10682 | *EBP* | 0.326 | 53.741 | 4.05E−09 |
| 255 | 3855 | *KRT7* | −0.328 | 53.732 | 4.07E−09 |
| 256 | 197257 | *LDHD* | 0.328 | 53.686 | 4.17E−09 |
| 257 | 6289 | *SAA2* | 0.321 | 53.663 | 4.22E−09 |
| 258 | 10928 | *RALBP1* | −0.358 | 53.628 | 4.29E−09 |
| 259 | 27233 | *SULT1C4* | 0.328 | 53.626 | 4.30E−09 |
| 260 | 80111 | *C3orf36* | −0.318 | 53.595 | 4.37E−09 |
| 261 | 307 | *ANXA4* | −0.317 | 53.579 | 4.40E−09 |

| | | | | | |
|---|---|---|---|---|---|
| 262 | 50862 | *RNF141* | −0.436 | 53.508 | 4.56E−09 |
| 263 | 4485 | *MST1* | 0.335 | 53.495 | 4.60E−09 |
| 264 | 2946 | *GSTM2* | 0.334 | 53.473 | 4.65E−09 |
| 265 | 3148 | *HMGB2* | 0.322 | 53.448 | 4.71E−09 |
| 266 | 64757 | *MTARC1* | 0.378 | 53.319 | 5.03E−09 |
| 267 | 420 | *ART4* | 0.456 | 53.315 | 5.04E−09 |
| 268 | 342979 | *PALM3* | 0.327 | 53.196 | 5.35E−09 |
| 269 | 10615 | *SPAG5* | 0.328 | 52.985 | 5.96E−09 |
| 270 | 84898 | *PLXDC2* | −0.339 | 52.977 | 5.98E−09 |
| 271 | 64859 | *NABP1* | −0.336 | 52.932 | 6.12E−09 |
| 272 | 1E+08 | *IPO5P1* | −0.332 | 52.890 | 6.25E−09 |
| 273 | 2202 | *EFEMP1* | −0.346 | 52.805 | 6.53E−09 |
| 274 | 2590 | *GALNT2* | −0.310 | 52.780 | 6.61E−09 |
| 275 | 5210 | *PFKFB4* | 0.312 | 52.728 | 6.79E−09 |
| 276 | 203111 | *ERICH5* | −0.310 | 52.638 | 7.11E−09 |
| 277 | 5066 | *PAM* | −0.372 | 52.527 | 7.52E−09 |
| 278 | 326625 | *MMAB* | 0.320 | 52.521 | 7.54E−09 |
| 279 | 57561 | *ARRDC3* | −0.334 | 52.487 | 7.68E−09 |
| 280 | 57504 | *MTA3* | 0.313 | 52.444 | 7.85E−09 |
| 281 | 5708 | *PSMD2* | −0.371 | 52.374 | 8.13E−09 |
| 282 | 2150 | *F2RL1* | −0.323 | 52.325 | 8.34E−09 |
| 283 | 3675 | *ITGA3* | −0.313 | 52.192 | 8.92E−09 |
| 284 | 169044 | *COL22A1* | −0.312 | 52.054 | 9.57E−09 |
| 285 | 124359 | *CDYL2* | −0.359 | 52.020 | 9.74E−09 |
| 286 | 1264 | *CNN1* | −0.353 | 52.015 | 9.76E−09 |
| 287 | 80223 | *RAB11FIP1* | −0.352 | 51.904 | 1.03E−08 |
| 288 | 9540 | *TP53I3* | −0.323 | 51.892 | 1.04E−08 |
| 289 | 3373 | *HYAL1* | 0.312 | 51.864 | 1.05E−08 |
| 290 | 29978 | *UBQLN2* | −0.324 | 51.849 | 1.06E−08 |
| 291 | 80256 | *FAM214B* | −0.320 | 51.782 | 1.10E−08 |
| 292 | 7905 | *REEP5* | −0.339 | 51.782 | 1.10E−08 |
| 293 | 51232 | *CRIM1* | −0.369 | 51.734 | 1.13E−08 |
| 294 | 51085 | *MLXIPL* | 0.337 | 51.623 | 1.19E−08 |
| 295 | 55540 | *IL17RB* | 0.322 | 51.617 | 1.20E−08 |
| 296 | 2155 | *F7* | 0.328 | 51.608 | 1.20E−08 |
| 297 | 187 | *APLNR* | 0.335 | 51.598 | 1.21E−08 |
| 298 | 308 | *ANXA5* | −0.327 | 51.581 | 1.22E−08 |
| 299 | 165257 | *C1QL2* | −0.372 | 51.557 | 1.23E−08 |
| 300 | 55347 | *ABHD10* | 0.346 | 51.508 | 1.26E−08 |
| 301 | 57633 | *LRRN1* | −0.340 | 51.489 | 1.28E−08 |
| 302 | 1982 | *EIF4G2* | −0.404 | 51.439 | 1.31E−08 |
| 303 | 6936 | *GCFC2* | 0.311 | 51.355 | 1.37E−08 |
| 304 | 10580 | *SORBS1* | 0.306 | 51.294 | 1.41E−08 |
| 305 | 91283 | *MSANTD3* | −0.337 | 51.284 | 1.42E−08 |
| 306 | 3840 | *KPNA4* | −0.414 | 51.245 | 1.45E−08 |
| 307 | 388015 | *RTL1* | 0.316 | 51.192 | 1.48E−08 |
| 308 | 84617 | *TUBB6* | −0.353 | 51.178 | 1.50E−08 |
| 309 | 3915 | *LAMC1* | −0.401 | 51.105 | 1.55E−08 |
| 310 | 341 | *APOC1* | 0.314 | 50.806 | 1.81E−08 |
| 311 | 91010 | *FMNL3* | 0.333 | 50.756 | 1.85E−08 |
| 312 | 57480 | *PLEKHG1* | −0.336 | 50.746 | 1.86E−08 |
| 313 | 8565 | *YARS1* | 0.332 | 50.733 | 1.88E−08 |
| 314 | 5349 | *FXYD3* | 0.293 | 50.682 | 1.92E−08 |

| | | | | | |
|---|---|---|---|---|---|
| 315 | 53826 | *FXYD6* | 0.344 | 50.682 | 1.93E−08 |
| 316 | 9104 | *RGN* | 0.306 | 50.674 | 1.93E−08 |
| 317 | 401251 | *SAPCD1* | 0.338 | 50.663 | 1.94E−08 |
| 318 | 2000 | *ELF4* | −0.326 | 50.608 | 2.00E−08 |
| 319 | 284340 | *CXCL17* | 0.317 | 50.582 | 2.03E−08 |
| 320 | 56271 | *BEX4* | 0.355 | 50.487 | 2.13E−08 |
| 321 | 2984 | *GUCY2C* | 0.310 | 50.447 | 2.17E−08 |
| 322 | 54853 | *WDR55* | −0.371 | 50.444 | 2.17E−08 |
| 323 | 3786 | *KCNQ3* | −0.330 | 50.417 | 2.20E−08 |
| 324 | 55740 | *ENAH* | −0.355 | 50.413 | 2.21E−08 |
| 325 | 80114 | *BICC1* | −0.352 | 50.376 | 2.25E−08 |
| 326 | 64061 | *TSPYL2* | 0.314 | 50.362 | 2.27E−08 |
| 327 | 84962 | *AJUBA* | −0.321 | 50.313 | 2.32E−08 |
| 328 | 189 | *AGXT* | 0.345 | 50.232 | 2.42E−08 |
| 329 | 4673 | *NAP1L1* | −0.350 | 50.117 | 2.57E−08 |
| 330 | 5165 | *PDK3* | 0.325 | 50.082 | 2.61E−08 |
| 331 | 23098 | *SARM1* | −0.318 | 50.079 | 2.62E−08 |
| 332 | 7048 | *TGFBR2* | −0.384 | 50.001 | 2.72E−08 |
| 333 | 4345 | *CD200* | −0.315 | 49.979 | 2.75E−08 |
| 334 | 1244 | *ABCC2* | 0.314 | 49.955 | 2.79E−08 |
| 335 | 121512 | *FGD4* | −0.386 | 49.952 | 2.79E−08 |
| 336 | 26005 | *C2CD3* | −0.321 | 49.911 | 2.85E−08 |
| 337 | 57142 | *RTN4* | −0.326 | 49.891 | 2.88E−08 |
| 338 | 64097 | *EPB41L4A* | −0.323 | 49.838 | 2.96E−08 |
| 339 | 6996 | *TDG* | −0.378 | 49.750 | 3.10E−08 |
| 340 | 400221 | *FLJ22447* | −0.309 | 49.630 | 3.29E−08 |
| 341 | 5062 | *PAK2* | −0.416 | 49.590 | 3.36E−08 |
| 342 | 39 | *ACAT2* | 0.353 | 49.584 | 3.37E−08 |
| 343 | 11108 | *PRDM4* | −0.376 | 49.572 | 3.39E−08 |
| 344 | 114800 | *CCDC85A* | −0.327 | 49.503 | 3.51E−08 |
| 345 | 2555 | *GABRA2* | −0.329 | 49.493 | 3.53E−08 |
| 346 | 598 | *BCL2L1* | −0.333 | 49.474 | 3.56E−08 |
| 347 | 1303 | *COL12A1* | −0.332 | 49.458 | 3.59E−08 |
| 348 | 5796 | *PTPRK* | −0.473 | 49.387 | 3.72E−08 |
| 349 | 10840 | *ALDH1L1* | 0.309 | 49.346 | 3.80E−08 |
| 350 | 5021 | *OXTR* | −0.322 | 49.328 | 3.84E−08 |
| 351 | 217 | *ALDH2* | 0.348 | 49.288 | 3.92E−08 |
| 352 | 10397 | *NDRG1* | 0.302 | 49.278 | 3.94E−08 |
| 353 | 64902 | *AGXT2* | 0.310 | 49.208 | 4.08E−08 |
| 354 | 4439 | *MSH5* | 0.331 | 49.193 | 4.11E−08 |
| 355 | 25957 | *PNISR* | 0.330 | 49.176 | 4.15E−08 |
| 356 | 3854 | *KRT6B* | −0.312 | 49.151 | 4.20E−08 |
| 357 | 6645 | *SNTB2* | −0.354 | 49.147 | 4.21E−08 |
| 358 | 84274 | *COQ5* | 0.300 | 49.131 | 4.24E−08 |
| 359 | 2013 | *EMP2* | 0.312 | 49.071 | 4.38E−08 |
| 360 | 1.01E+08 | *LINC00963* | −0.318 | 49.009 | 4.52E−08 |
| 361 | 9074 | *CLDN6* | −0.326 | 48.909 | 4.75E−08 |
| 362 | 10082 | *GPC6* | −0.405 | 48.889 | 4.80E−08 |
| 363 | 54442 | *KCTD5* | −0.374 | 48.789 | 5.05E−08 |
| 364 | 11161 | *ERG28* | 0.347 | 48.542 | 5.73E−08 |
| 365 | 256394 | *SERPINA11* | 0.307 | 48.530 | 5.77E−08 |
| 366 | 51202 | *DDX47* | −0.357 | 48.496 | 5.87E−08 |
| 367 | 2321 | *FLT1* | −0.313 | 48.454 | 5.99E−08 |

| | | | | | |
|---|---|---|---|---|---|
| 368 | 196410 | *METTL7B* | 0.315 | 48.436 | 6.05E−08 |
| 369 | 254427 | *PROSER2* | −0.390 | 48.421 | 6.10E−08 |
| 370 | 56672 | *AKIP1* | −0.298 | 48.371 | 6.25E−08 |
| 371 | 1000 | *CDH2* | −0.354 | 48.366 | 6.27E−08 |
| 372 | 11072 | *DUSP14* | −0.309 | 48.319 | 6.42E−08 |
| 373 | 132158 | *GLYCTK* | 0.327 | 48.299 | 6.49E−08 |
| 374 | 51573 | *GDE1* | −0.318 | 48.218 | 6.76E−08 |
| 375 | 9520 | *NPEPPS* | −0.446 | 48.191 | 6.85E−08 |
| 376 | 1946 | *EFNA5* | −0.319 | 48.172 | 6.92E−08 |
| 377 | 57493 | *HEG1* | −0.359 | 48.168 | 6.93E−08 |
| 378 | 7171 | *TPM4* | −0.334 | 48.134 | 7.06E−08 |
| 379 | 364 | *AQP7* | 0.318 | 48.133 | 7.06E−08 |
| 380 | 3482 | *IGF2R* | −0.436 | 48.129 | 7.07E−08 |
| 381 | 6575 | *SLC20A2* | −0.336 | 48.083 | 7.24E−08 |
| 382 | 10276 | *NET1* | 0.346 | 48.036 | 7.42E−08 |
| 383 | 116835 | *HSPA12B* | 0.312 | 47.983 | 7.62E−08 |
| 384 | 51430 | *SUCO* | −0.387 | 47.977 | 7.64E−08 |
| 385 | 3312 | *HSPA8* | −0.486 | 47.968 | 7.68E−08 |
| 386 | 6274 | *S100A3* | −0.304 | 47.910 | 7.91E−08 |
| 387 | 93145 | *OLFM2* | −0.381 | 47.892 | 7.98E−08 |
| 388 | 5340 | *PLG* | 0.335 | 47.877 | 8.04E−08 |
| 389 | 230 | *ALDOC* | 0.288 | 47.802 | 8.36E−08 |
| 390 | 9118 | *INA* | −0.342 | 47.734 | 8.65E−08 |
| 391 | 3659 | *IRF1* | −0.309 | 47.690 | 8.85E−08 |
| 392 | 401463 | *LOC401463* | −0.296 | 47.507 | 9.72E−08 |
| 393 | 10072 | *DPP3* | −0.319 | 47.497 | 9.77E−08 |
| 394 | 4776 | *NFATC4* | 0.336 | 47.486 | 9.82E−08 |
| 395 | 200931 | *SLC51A* | 0.294 | 47.298 | 1.08E−07 |
| 396 | 7534 | *YWHAZ* | −0.338 | 47.268 | 1.10E−07 |
| 397 | 8516 | *ITGA8* | 0.298 | 47.240 | 1.11E−07 |
| 398 | 8609 | *KLF7* | −0.376 | 47.204 | 1.13E−07 |
| 399 | 728377 | *ARHGEF34P* | −0.321 | 47.180 | 1.15E−07 |
| 400 | 4644 | *MYO5A* | −0.347 | 47.050 | 1.23E−07 |
| 401 | 5878 | *RAB5C* | −0.300 | 47.021 | 1.24E−07 |
| 402 | 23012 | *STK38L* | −0.340 | 46.953 | 1.29E−07 |
| 403 | 9747 | *TCAF1* | −0.389 | 46.940 | 1.30E−07 |
| 404 | 152816 | *ODAPH* | −0.309 | 46.850 | 1.36E−07 |
| 405 | 462 | *SERPINC1* | 0.321 | 46.817 | 1.38E−07 |
| 406 | 284422 | *SMIM24* | 0.303 | 46.738 | 1.44E−07 |
| 407 | 388403 | *YPEL2* | −0.356 | 46.716 | 1.45E−07 |
| 408 | 3662 | *IRF4* | −0.293 | 46.716 | 1.45E−07 |
| 409 | 486 | *FXYD2* | 0.309 | 46.653 | 1.50E−07 |
| 410 | 1436 | *CSF1R* | −0.299 | 46.562 | 1.57E−07 |
| 411 | 51053 | *GMNN* | 0.312 | 46.476 | 1.64E−07 |
| 412 | 10462 | *CLEC10A* | 0.312 | 46.449 | 1.67E−07 |
| 413 | 23673 | *STX12* | −0.362 | 46.397 | 1.71E−07 |
| 414 | 9644 | *SH3PXD2A* | 0.330 | 46.394 | 1.71E−07 |
| 415 | 4891 | *SLC11A2* | 0.326 | 46.335 | 1.77E−07 |
| 416 | 55690 | *PACS1* | −0.389 | 46.307 | 1.79E−07 |
| 417 | 445328 | *ARHGEF35* | −0.328 | 46.217 | 1.88E−07 |
| 418 | 55727 | *BTBD7* | −0.416 | 46.199 | 1.89E−07 |
| 419 | 83461 | *CDCA3* | 0.322 | 46.056 | 2.04E−07 |
| 420 | 23158 | *TBC1D9* | −0.329 | 46.021 | 2.07E−07 |

| | | | | | |
|---|---|---|---|---|---|
| 421 | 2139 | *EYA2* | −0.293 | 45.932 | 2.17E−07 |
| 422 | 26999 | *CYFIP2* | −0.313 | 45.899 | 2.21E−07 |
| 423 | 9056 | *SLC7A7* | −0.314 | 45.864 | 2.25E−07 |
| 424 | 8643 | *PTCH2* | 0.348 | 45.771 | 2.36E−07 |
| 425 | 1644 | *DDC* | 0.316 | 45.719 | 2.42E−07 |
| 426 | 5727 | *PTCH1* | 0.316 | 45.700 | 2.44E−07 |
| 427 | 3177 | *SLC29A2* | 0.330 | 45.694 | 2.45E−07 |
| 428 | 51776 | *MAP3K20* | −0.414 | 45.676 | 2.47E−07 |
| 429 | 22982 | *DIP2C* | −0.354 | 45.451 | 2.77E−07 |
| 430 | 29970 | *SCHIP1* | −0.321 | 45.417 | 2.82E−07 |
| 431 | 83892 | *KCTD10* | −0.312 | 45.355 | 2.91E−07 |
| 432 | 57222 | *ERGIC1* | −0.312 | 45.334 | 2.94E−07 |
| 433 | 3685 | *ITGAV* | −0.393 | 45.322 | 2.96E−07 |
| 434 | 7165 | *TPD52L2* | −0.319 | 45.288 | 3.01E−07 |
| 435 | 11221 | *DUSP10* | −0.306 | 45.236 | 3.10E−07 |
| 436 | 8991 | *SELENBP1* | 0.333 | 45.205 | 3.15E−07 |
| 437 | 9169 | *SCAF11* | −0.455 | 45.175 | 3.19E−07 |
| 438 | 26010 | *SPATS2L* | −0.305 | 45.146 | 3.24E−07 |
| 439 | 6553 | *SLC9A5* | 0.368 | 45.131 | 3.27E−07 |
| 440 | 571 | *BACH1* | −0.384 | 45.091 | 3.33E−07 |
| 441 | 91614 | *DEPDC7* | 0.297 | 45.081 | 3.35E−07 |
| 442 | 54097 | *FAM3B* | 0.289 | 45.058 | 3.39E−07 |
| 443 | 55211 | *DPPA4* | 0.300 | 45.054 | 3.40E−07 |
| 444 | 861 | *RUNX1* | −0.295 | 45.040 | 3.42E−07 |
| 445 | 54346 | *UNC93A* | 0.290 | 44.972 | 3.54E−07 |
| 446 | 29940 | *DSE* | −0.287 | 44.970 | 3.55E−07 |
| 447 | 51088 | *KLHL5* | −0.346 | 44.955 | 3.57E−07 |
| 448 | 115290 | *FBXO17* | 0.314 | 44.861 | 3.75E−07 |
| 449 | 54915 | *YTHDF1* | −0.301 | 44.794 | 3.88E−07 |
| 450 | 10247 | *RIDA* | 0.293 | 44.768 | 3.93E−07 |
| 451 | 9079 | *LDB2* | 0.306 | 44.749 | 3.97E−07 |
| 452 | 56997 | *COQ8A* | 0.306 | 44.724 | 4.02E−07 |
| 453 | 200010 | *SLC5A9* | 0.295 | 44.681 | 4.11E−07 |
| 454 | 23594 | *ORC6* | 0.297 | 44.658 | 4.16E−07 |
| 455 | 51363 | *CHST15* | −0.308 | 44.650 | 4.18E−07 |
| 456 | 9185 | *REPS2* | −0.328 | 44.641 | 4.19E−07 |
| 457 | 80221 | *ACSF2* | 0.290 | 44.621 | 4.24E−07 |
| 458 | 10886 | *NPFFR2* | −0.305 | 44.619 | 4.24E−07 |
| 459 | 10024 | *TROAP* | 0.318 | 44.502 | 4.50E−07 |
| 460 | 51084 | *CRYL1* | 0.300 | 44.498 | 4.51E−07 |
| 461 | 516 | *ATP5MC1* | 0.330 | 44.496 | 4.52E−07 |
| 462 | 5320 | *PLA2G2A* | 0.311 | 44.441 | 4.65E−07 |
| 463 | 9232 | *PTTG1* | 0.305 | 44.436 | 4.66E−07 |
| 464 | 116984 | *ARAP2* | −0.376 | 44.424 | 4.69E−07 |
| 465 | 7984 | *ARHGEF5* | −0.333 | 44.397 | 4.75E−07 |
| 466 | 635 | *BHMT* | 0.292 | 44.390 | 4.77E−07 |
| 467 | 377 | *ARF3* | −0.298 | 44.336 | 4.90E−07 |
| 468 | 51582 | *AZIN1* | −0.397 | 44.329 | 4.92E−07 |
| 469 | 51744 | *CD244* | 0.289 | 44.325 | 4.93E−07 |
| 470 | 55315 | *SLC29A3* | −0.297 | 44.290 | 5.02E−07 |
| 471 | 25819 | *NOCT* | −0.293 | 44.269 | 5.07E−07 |
| 472 | 66000 | *TMEM108* | −0.294 | 44.268 | 5.07E−07 |
| 473 | 1293 | *COL6A3* | 0.306 | 44.160 | 5.36E−07 |

| | | | | | |
|---|---|---|---|---|---|
| 474 | 1.02E+08 | *LOC101928034* | −0.300 | 44.100 | 5.53E−07 |
| 475 | 2304 | *FOXE1* | −0.303 | 44.086 | 5.57E−07 |
| 476 | 9786 | *KIAA0586* | −0.330 | 43.995 | 5.83E−07 |
| 477 | 283375 | *SLC39A5* | 0.308 | 43.979 | 5.88E−07 |
| 478 | 90861 | *JPT2* | −0.351 | 43.972 | 5.90E−07 |
| 479 | 159195 | *USP54* | −0.330 | 43.965 | 5.93E−07 |
| 480 | 79960 | *JADE1* | −0.305 | 43.954 | 5.96E−07 |
| 481 | 11001 | *SLC27A2* | 0.287 | 43.926 | 6.04E−07 |
| 482 | 9693 | *RAPGEF2* | −0.339 | 43.858 | 6.26E−07 |
| 483 | 1612 | *DAPK1* | −0.289 | 43.843 | 6.31E−07 |
| 484 | 10197 | *PSME3* | −0.359 | 43.824 | 6.37E−07 |
| 485 | 1001 | *CDH3* | −0.301 | 43.823 | 6.37E−07 |
| 486 | 26508 | *HEYL* | 0.304 | 43.757 | 6.59E−07 |
| 487 | 9900 | *SV2A* | 0.317 | 43.724 | 6.70E−07 |
| 488 | 358 | *AQP1* | 0.314 | 43.722 | 6.71E−07 |
| 489 | 23484 | *LEPROTL1* | −0.285 | 43.717 | 6.73E−07 |
| 490 | 11041 | *B4GAT1* | 0.312 | 43.646 | 6.98E−07 |
| 491 | 1E+08 | *LOC100419583* | −0.294 | 43.631 | 7.03E−07 |
| 492 | 5792 | *PTPRF* | −0.292 | 43.598 | 7.15E−07 |
| 493 | 8935 | *SKAP2* | −0.363 | 43.498 | 7.52E−07 |
| 494 | 5420 | *PODXL* | 0.312 | 43.438 | 7.76E−07 |
| 495 | 6047 | *RNF4* | −0.310 | 43.436 | 7.76E−07 |
| 496 | 3720 | *JARID2* | 0.302 | 43.377 | 8.00E−07 |
| 497 | 158586 | *ZXDB* | −0.341 | 43.372 | 8.02E−07 |
| 498 | 89970 | *RSPRY1* | −0.349 | 43.344 | 8.14E−07 |
| 499 | 50507 | *NOX4* | −0.292 | 43.254 | 8.52E−07 |
| 500 | 85463 | *ZC3H12C* | −0.334 | 43.253 | 8.53E−07 |
| 501 | 54886 | *PLPPR1* | 0.292 | 43.237 | 8.60E−07 |
| 502 | 10050 | *SLC17A4* | 0.285 | 43.219 | 8.68E−07 |
| 503 | 158326 | *FREM1* | 0.283 | 43.196 | 8.78E−07 |
| 504 | 9832 | *JAKMIP2* | −0.309 | 43.177 | 8.86E−07 |
| 505 | 7046 | *TGFBR1* | −0.353 | 43.165 | 8.92E−07 |
| 506 | 11127 | *KIF3A* | −0.378 | 43.136 | 9.05E−07 |
| 507 | 3213 | *HOXB3* | 0.329 | 43.104 | 9.20E−07 |
| 508 | 22808 | *MRAS* | −0.281 | 43.079 | 9.32E−07 |
| 509 | 5293 | *PIK3CD* | −0.314 | 43.077 | 9.33E−07 |
| 510 | 10133 | *OPTN* | −0.358 | 43.065 | 9.38E−07 |
| 511 | 11004 | *KIF2C* | 0.298 | 42.967 | 9.87E−07 |
| 512 | 94081 | *SFXN1* | 0.339 | 42.937 | 1.00E−06 |
| 513 | 64780 | *MICAL1* | −0.353 | 42.900 | 1.02E−06 |
| 514 | 5089 | *PBX2* | 0.314 | 42.822 | 1.06E−06 |
| 515 | 8309 | *ACOX2* | 0.287 | 42.726 | 1.12E−06 |
| 516 | 3895 | *KTN1* | −0.424 | 42.698 | 1.13E−06 |
| 517 | 7107 | *GPR137B* | −0.297 | 42.662 | 1.15E−06 |
| 518 | 84647 | *PLA2G12B* | 0.292 | 42.643 | 1.16E−06 |
| 519 | 51669 | *SARAF* | −0.316 | 42.638 | 1.17E−06 |
| 520 | 25996 | *REXO2* | −0.284 | 42.600 | 1.19E−06 |
| 521 | 23229 | *ARHGEF9* | 0.295 | 42.571 | 1.21E−06 |
| 522 | 85377 | *MICALL1* | −0.314 | 42.560 | 1.22E−06 |
| 523 | 23677 | *SH3BP4* | −0.319 | 42.488 | 1.26E−06 |
| 524 | 7380 | *UPK3A* | 0.301 | 42.400 | 1.32E−06 |
| 525 | 64946 | *CENPH* | 0.300 | 42.386 | 1.33E−06 |
| 526 | 54751 | *FBLIM1* | −0.341 | 42.372 | 1.34E−06 |

| | | | | | |
|---|---|---|---|---|---|
| 527 | 53944 | *CSNK1G1* | −0.440 | 42.356 | 1.35E−06 |
| 528 | 9421 | *HAND1* | 0.319 | 42.344 | 1.36E−06 |
| 529 | 2819 | *GPD1* | 0.284 | 42.332 | 1.36E−06 |
| 530 | 2224 | *FDPS* | 0.308 | 42.271 | 1.41E−06 |
| 531 | 9547 | *CXCL14* | 0.313 | 42.165 | 1.49E−06 |
| 532 | 51317 | *PHF21A* | 0.297 | 42.123 | 1.52E−06 |
| 533 | 29968 | *PSAT1* | 0.296 | 42.118 | 1.52E−06 |
| 534 | 1845 | *DUSP3* | −0.332 | 42.107 | 1.53E−06 |
| 535 | 7162 | *TPBG* | −0.320 | 42.100 | 1.54E−06 |
| 536 | 3184 | *HNRNPD* | 0.295 | 42.083 | 1.55E−06 |
| 537 | 4547 | *MTTP* | 0.280 | 42.043 | 1.58E−06 |
| 538 | 5118 | *PCOLCE* | 0.308 | 42.039 | 1.59E−06 |
| 539 | 221393 | *ADGRF4* | −0.309 | 42.009 | 1.61E−06 |
| 540 | 26020 | *LRP10* | −0.303 | 41.991 | 1.63E−06 |
| 541 | 5160 | *PDHA1* | 0.289 | 41.974 | 1.64E−06 |
| 542 | 254122 | *SNX32* | 0.305 | 41.952 | 1.66E−06 |
| 543 | 818 | *CAMK2G* | 0.280 | 41.951 | 1.66E−06 |
| 544 | 10863 | *ADAM28* | −0.303 | 41.934 | 1.67E−06 |
| 545 | 284325 | *C19orf54* | 0.397 | 41.917 | 1.69E−06 |
| 546 | 678 | *ZFP36L2* | 0.314 | 41.887 | 1.71E−06 |
| 547 | 9963 | *SLC23A1* | 0.276 | 41.753 | 1.84E−06 |
| 548 | 8448 | *DOC2A* | 0.370 | 41.747 | 1.84E−06 |
| 549 | 2919 | *CXCL1* | −0.277 | 41.718 | 1.87E−06 |
| 550 | 60626 | *RIC8A* | −0.306 | 41.692 | 1.89E−06 |
| 551 | 146850 | *PIK3R6* | 0.294 | 41.662 | 1.92E−06 |
| 552 | 10446 | *LRRN2* | −0.308 | 41.644 | 1.94E−06 |
| 553 | 25791 | *NGEF* | 0.301 | 41.634 | 1.95E−06 |
| 554 | 9180 | *OSMR* | −0.356 | 41.612 | 1.97E−06 |
| 555 | 11021 | *RAB35* | −0.294 | 41.611 | 1.97E−06 |
| 556 | 220323 | *OAF* | 0.335 | 41.596 | 1.99E−06 |
| 557 | 3716 | *JAK1* | −0.418 | 41.586 | 2.00E−06 |
| 558 | 55556 | *ENOSF1* | 0.294 | 41.572 | 2.01E−06 |
| 559 | 2239 | *GPC4* | −0.303 | 41.541 | 2.05E−06 |
| 560 | 283152 | *CCDC153* | 0.288 | 41.534 | 2.05E−06 |
| 561 | 9507 | *ADAMTS4* | 0.394 | 41.530 | 2.06E−06 |
| 562 | 2869 | *GRK5* | 0.293 | 41.523 | 2.07E−06 |
| 563 | 9980 | *DOP1B* | −0.303 | 41.496 | 2.09E−06 |
| 564 | 7529 | *YWHAB* | −0.405 | 41.486 | 2.10E−06 |
| 565 | 22800 | *RRAS2* | −0.274 | 41.435 | 2.16E−06 |
| 566 | 11065 | *UBE2C* | 0.314 | 41.419 | 2.18E−06 |
| 567 | 1.01E+08 | *DNM3OS* | 0.297 | 41.409 | 2.19E−06 |
| 568 | 79170 | *PRR15L* | 0.276 | 41.370 | 2.23E−06 |
| 569 | 433 | *ASGR2* | 0.308 | 41.325 | 2.29E−06 |
| 570 | 3916 | *LAMP1* | −0.283 | 41.313 | 2.30E−06 |
| 571 | 6492 | *SIM1* | −0.289 | 41.312 | 2.30E−06 |
| 572 | 51114 | *ZDHHC9* | −0.299 | 41.307 | 2.31E−06 |
| 573 | 9058 | *SLC13A2* | 0.275 | 41.289 | 2.33E−06 |
| 574 | 9819 | *TSC22D2* | −0.305 | 41.241 | 2.38E−06 |
| 575 | 93 | *ACVR2B* | 0.297 | 41.237 | 2.39E−06 |
| 576 | 23705 | *CADM1* | −0.306 | 41.182 | 2.46E−06 |
| 577 | 51312 | *SLC25A37* | 0.290 | 41.110 | 2.55E−06 |
| 578 | 23225 | *NUP210* | 0.295 | 41.067 | 2.61E−06 |
| 579 | 56181 | *MTFR1L* | 0.315 | 41.050 | 2.63E−06 |

| | | | | | |
|---|---|---|---|---|---|
| 580 | 32 | *ACACB* | 0.270 | 41.020 | 2.67E−06 |
| 581 | 286343 | *LURAP1L* | −0.288 | 40.968 | 2.74E−06 |
| 582 | 85004 | *RERG* | 0.289 | 40.921 | 2.81E−06 |
| 583 | 286887 | *KRT6C* | −0.284 | 40.921 | 2.81E−06 |
| 584 | 166824 | *RASSF6* | −0.342 | 40.898 | 2.84E−06 |
| 585 | 8840 | *CCN4* | 0.313 | 40.893 | 2.85E−06 |
| 586 | 4239 | *MFAP4* | 0.303 | 40.869 | 2.89E−06 |
| 587 | 22 | *ABCB7* | 0.328 | 40.810 | 2.97E−06 |
| 588 | 58510 | *PRODH2* | 0.280 | 40.810 | 2.97E−06 |
| 589 | 57464 | *STRIP2* | −0.292 | 40.787 | 3.01E−06 |
| 590 | 1357 | *CPA1* | 0.289 | 40.761 | 3.05E−06 |
| 591 | 55884 | *WSB2* | −0.296 | 40.756 | 3.06E−06 |
| 592 | 1.02E+08 | *LINC01108* | 0.279 | 40.751 | 3.07E−06 |
| 593 | 9924 | *PAN2* | 0.278 | 40.726 | 3.10E−06 |
| 594 | 91433 | *RCCD1* | 0.416 | 40.665 | 3.20E−06 |
| 595 | 3549 | *IHH* | 0.335 | 40.657 | 3.22E−06 |
| 596 | 858 | *CAV2* | −0.312 | 40.647 | 3.23E−06 |
| 597 | 283219 | *KCTD21* | −0.296 | 40.592 | 3.32E−06 |
| 598 | 55227 | *LRRC1* | −0.276 | 40.578 | 3.35E−06 |
| 599 | 3590 | *IL11RA* | 0.321 | 40.576 | 3.35E−06 |
| 600 | 23326 | *USP22* | −0.283 | 40.537 | 3.42E−06 |
| 601 | 166929 | *SGMS2* | −0.364 | 40.520 | 3.45E−06 |
| 602 | 3242 | *HPD* | 0.312 | 40.451 | 3.57E−06 |
| 603 | 83891 | *SNX25* | −0.334 | 40.451 | 3.57E−06 |
| 604 | 57715 | *SEMA4G* | 0.311 | 40.438 | 3.60E−06 |
| 605 | 57678 | *GPAM* | 0.364 | 40.422 | 3.63E−06 |
| 606 | 27075 | *TSPAN13* | −0.290 | 40.400 | 3.67E−06 |
| 607 | 6844 | *VAMP2* | 0.301 | 40.319 | 3.82E−06 |
| 608 | 160851 | *DGKH* | −0.312 | 40.284 | 3.89E−06 |
| 609 | 23582 | *CCNDBP1* | −0.315 | 40.267 | 3.93E−06 |
| 610 | 79153 | *GDPD3* | 0.284 | 40.259 | 3.94E−06 |
| 611 | 54521 | *WDR44* | −0.389 | 40.258 | 3.94E−06 |
| 612 | 404093 | *CUEDC1* | −0.342 | 40.251 | 3.96E−06 |
| 613 | 130733 | *TMEM178A* | 0.328 | 40.135 | 4.20E−06 |
| 614 | 151636 | *DTX3L* | −0.354 | 40.129 | 4.21E−06 |
| 615 | 229 | *ALDOB* | 0.274 | 40.124 | 4.22E−06 |
| 616 | 242 | *ALOX12B* | 0.286 | 40.124 | 4.22E−06 |
| 617 | 23492 | *CBX7* | 0.303 | 40.094 | 4.29E−06 |
| 618 | 1441 | *CSF3R* | 0.312 | 40.080 | 4.32E−06 |
| 619 | 54810 | *GIPC2* | 0.303 | 40.072 | 4.34E−06 |
| 620 | 388969 | *C2orf68* | 0.326 | 40.067 | 4.35E−06 |
| 621 | 10938 | *EHD1* | −0.387 | 40.060 | 4.37E−06 |
| 622 | 54842 | *MFSD6* | −0.307 | 40.056 | 4.37E−06 |
| 623 | 7067 | *THRA* | 0.472 | 40.038 | 4.42E−06 |
| 624 | 114804 | *RNF157* | 0.292 | 40.021 | 4.45E−06 |
| 625 | 84830 | *ADTRP* | 0.280 | 40.009 | 4.48E−06 |
| 626 | 57048 | *PLSCR3* | −0.426 | 40.009 | 4.48E−06 |
| 627 | 3082 | *HGF* | 0.284 | 39.971 | 4.57E−06 |
| 628 | 3486 | *IGFBP3* | 0.313 | 39.964 | 4.59E−06 |
| 629 | 161176 | *SYNE3* | 0.480 | 39.949 | 4.62E−06 |
| 630 | 2629 | *GBA* | −0.294 | 39.910 | 4.71E−06 |
| 631 | 9921 | *RNF10* | −0.317 | 39.878 | 4.79E−06 |
| 632 | 9992 | *KCNE2* | 0.268 | 39.863 | 4.83E−06 |

| | | | | | |
|---|---|---|---|---|---|
| 633 | 55312 | *RFK* | −0.296 | 39.816 | 4.95E−06 |
| 634 | 93594 | *TBC1D31* | 0.290 | 39.793 | 5.00E−06 |
| 635 | 163 | *AP2B1* | −0.388 | 39.793 | 5.01E−06 |
| 636 | 6886 | *TAL1* | −0.283 | 39.773 | 5.06E−06 |
| 637 | 728215 | *FAM155A* | −0.281 | 39.759 | 5.09E−06 |
| 638 | 53836 | *GPR87* | −0.299 | 39.754 | 5.11E−06 |
| 639 | 253769 | *WDR27* | 0.327 | 39.746 | 5.13E−06 |
| 640 | 64116 | *SLC39A8* | −0.312 | 39.736 | 5.15E−06 |
| 641 | 1649 | *DDIT3* | 0.299 | 39.733 | 5.16E−06 |
| 642 | 4094 | *MAF* | −0.276 | 39.697 | 5.26E−06 |
| 643 | 2294 | *FOXF1* | 0.308 | 39.662 | 5.35E−06 |
| 644 | 5358 | *PLS3* | −0.364 | 39.658 | 5.36E−06 |
| 645 | 56937 | *PMEPA1* | −0.314 | 39.651 | 5.38E−06 |
| 646 | 134553 | *C5orf24* | −0.321 | 39.636 | 5.42E−06 |
| 647 | 2317 | *FLNB* | −0.311 | 39.625 | 5.45E−06 |
| 648 | 137872 | *ADHFE1* | 0.292 | 39.621 | 5.47E−06 |
| 649 | 6581 | *SLC22A3* | 0.293 | 39.590 | 5.55E−06 |
| 650 | 2274 | *FHL2* | −0.301 | 39.570 | 5.61E−06 |
| 651 | 162387 | *MFSD6L* | 0.280 | 39.547 | 5.68E−06 |
| 652 | 5314 | *PKHD1* | −0.311 | 39.547 | 5.68E−06 |
| 653 | 51809 | *GALNT7* | −0.318 | 39.545 | 5.68E−06 |
| 654 | 90102 | *PHLDB2* | −0.392 | 39.529 | 5.73E−06 |
| 655 | 283383 | *ADGRD1* | 0.288 | 39.521 | 5.75E−06 |
| 656 | 55707 | *NECAP2* | −0.320 | 39.486 | 5.86E−06 |
| 657 | 26115 | *TANC2* | −0.328 | 39.398 | 6.13E−06 |
| 658 | 29919 | *RMC1* | −0.278 | 39.389 | 6.16E−06 |
| 659 | 3164 | *NR4A1* | 0.321 | 39.389 | 6.16E−06 |
| 660 | 26030 | *PLEKHG3* | −0.348 | 39.349 | 6.28E−06 |
| 661 | 203259 | *FAM219A* | −0.284 | 39.345 | 6.30E−06 |
| 662 | 56902 | *PNO1* | −0.320 | 39.339 | 6.31E−06 |
| 663 | 6783 | *SULT1E1* | 0.299 | 39.337 | 6.32E−06 |
| 664 | 64121 | *RRAGC* | −0.389 | 39.311 | 6.41E−06 |
| 665 | 1861 | *TOR1A* | −0.276 | 39.305 | 6.42E−06 |
| 666 | 284119 | *CAVIN1* | −0.306 | 39.288 | 6.48E−06 |
| 667 | 51195 | *RAPGEFL1* | 0.378 | 39.225 | 6.70E−06 |
| 668 | 80014 | *WWC2* | −0.351 | 39.215 | 6.73E−06 |
| 669 | 2331 | *FMOD* | 0.289 | 39.209 | 6.75E−06 |
| 670 | 319089 | *TTC6* | 0.276 | 39.183 | 6.84E−06 |
| 671 | 4134 | *MAP4* | −0.295 | 39.171 | 6.88E−06 |
| 672 | 6291 | *SAA4* | 0.284 | 39.139 | 7.00E−06 |
| 673 | 22898 | *DENND3* | −0.344 | 39.128 | 7.04E−06 |
| 674 | 91768 | *CABLES1* | −0.286 | 39.113 | 7.09E−06 |
| 675 | 6718 | *AKR1D1* | 0.276 | 39.057 | 7.30E−06 |
| 676 | 23092 | *ARHGAP26* | 0.278 | 39.018 | 7.44E−06 |
| 677 | 10962 | *MLLT11* | −0.283 | 38.999 | 7.51E−06 |
| 678 | 966 | *CD59* | −0.318 | 38.989 | 7.56E−06 |
| 679 | 4258 | *MGST2* | 0.278 | 38.982 | 7.58E−06 |
| 680 | 7403 | *KDM6A* | −0.428 | 38.968 | 7.63E−06 |
| 681 | 65010 | *SLC26A6* | 0.303 | 38.957 | 7.68E−06 |
| 682 | 1187 | *CLCNKA* | 0.291 | 38.924 | 7.81E−06 |
| 683 | 4351 | *MPI* | 0.278 | 38.907 | 7.88E−06 |
| 684 | 4046 | *LSP1* | 0.290 | 38.868 | 8.04E−06 |
| 685 | 4741 | *NEFM* | −0.283 | 38.865 | 8.05E−06 |

| | | | | | |
|---|---|---|---|---|---|
| 686 | 55937 | *APOM* | 0.281 | 38.821 | 8.23E−06 |
| 687 | 130074 | *FAM168B* | −0.376 | 38.769 | 8.45E−06 |
| 688 | 5764 | *PTN* | 0.284 | 38.769 | 8.46E−06 |
| 689 | 4224 | *MEP1A* | 0.279 | 38.758 | 8.51E−06 |
| 690 | 79987 | *SVEP1* | 0.289 | 38.757 | 8.51E−06 |
| 691 | 30851 | *TAX1BP3* | −0.283 | 38.748 | 8.55E−06 |
| 692 | 84656 | *GLYR1* | −0.298 | 38.740 | 8.58E−06 |
| 693 | 956 | *ENTPD3* | −0.271 | 38.734 | 8.61E−06 |
| 694 | 84129 | *ACAD11* | 0.296 | 38.724 | 8.65E−06 |
| 695 | 1282 | *COL4A1* | −0.294 | 38.692 | 8.80E−06 |
| 696 | 3033 | *HADH* | 0.286 | 38.662 | 8.93E−06 |
| 697 | 55037 | *PTCD3* | 0.301 | 38.633 | 9.06E−06 |
| 698 | 51652 | *CHMP3* | −0.327 | 38.605 | 9.20E−06 |
| 699 | 57465 | *TBC1D24* | 0.293 | 38.592 | 9.26E−06 |
| 700 | 55002 | *TMCO3* | −0.314 | 38.567 | 9.38E−06 |
| 701 | 1124 | *CHN2* | 0.272 | 38.512 | 9.65E−06 |
| 702 | 51330 | *TNFRSF12A* | −0.385 | 38.479 | 9.81E−06 |
| 703 | 2147 | *F2* | 0.295 | 38.463 | 9.89E−06 |
| 704 | 153129 | *SLC38A9* | −0.329 | 38.455 | 9.93E−06 |
| 705 | 8994 | *LIMD1* | −0.286 | 38.455 | 9.93E−06 |
| 706 | 2159 | *F10* | 0.298 | 38.419 | 1.01E−05 |
| 707 | 115209 | *OMA1* | 0.305 | 38.401 | 1.02E−05 |
| 708 | 57523 | *NYNRIN* | 0.281 | 38.394 | 1.02E−05 |
| 709 | 50486 | *G0S2* | 0.296 | 38.393 | 1.03E−05 |
| 710 | 6505 | *SLC1A1* | −0.304 | 38.390 | 1.03E−05 |
| 711 | 2813 | *GP2* | 0.289 | 38.356 | 1.04E−05 |
| 712 | 9455 | *HOMER2* | −0.283 | 38.354 | 1.05E−05 |
| 713 | 254863 | *TMEM256* | 0.354 | 38.350 | 1.05E−05 |
| 714 | 55273 | *TMEM100* | 0.289 | 38.329 | 1.06E−05 |
| 715 | 6271 | *S100A1* | 0.278 | 38.300 | 1.07E−05 |
| 716 | 55811 | *ADCY10* | 0.280 | 38.298 | 1.08E−05 |
| 717 | 84502 | *JPH4* | 0.290 | 38.295 | 1.08E−05 |
| 718 | 118788 | *PIK3AP1* | −0.310 | 38.211 | 1.13E−05 |
| 719 | 11112 | *HIBADH* | 0.272 | 38.161 | 1.15E−05 |
| 720 | 203328 | *SUSD3* | 0.289 | 38.154 | 1.16E−05 |
| 721 | 51303 | *FKBP11* | 0.326 | 38.098 | 1.19E−05 |
| 722 | 6324 | *SCN1B* | −0.308 | 38.088 | 1.20E−05 |
| 723 | 84978 | *FRMD5* | −0.293 | 38.084 | 1.20E−05 |
| 724 | 3189 | *HNRNPH3* | 0.295 | 38.056 | 1.22E−05 |
| 725 | 171425 | *CLYBL* | 0.262 | 38.028 | 1.24E−05 |
| 726 | 10563 | *CXCL13* | 0.280 | 38.012 | 1.25E−05 |
| 727 | 64968 | *MRPS6* | 0.281 | 38.001 | 1.25E−05 |
| 728 | 85236 | *H2BC12* | −0.283 | 37.988 | 1.26E−05 |
| 729 | 4329 | *ALDH6A1* | 0.281 | 37.945 | 1.29E−05 |
| 730 | 3481 | *IGF2* | 0.296 | 37.932 | 1.30E−05 |
| 731 | 146754 | *DNAH2* | 0.267 | 37.879 | 1.33E−05 |
| 732 | 6429 | *SRSF4* | 0.322 | 37.867 | 1.34E−05 |
| 733 | 23493 | *HEY2* | 0.278 | 37.836 | 1.36E−05 |
| 734 | 643650 | *LINC00842* | −0.275 | 37.836 | 1.36E−05 |
| 735 | 727758 | *ROCK1P1* | −0.285 | 37.800 | 1.39E−05 |
| 736 | 92421 | *CHMP4C* | −0.305 | 37.780 | 1.40E−05 |
| 737 | 79730 | *NSUN7* | 0.280 | 37.747 | 1.43E−05 |
| 738 | 1191 | *CLU* | 0.276 | 37.723 | 1.45E−05 |

| | | | | | |
|---|---|---|---|---|---|
| 739 | 9943 | *OXSR1* | −0.374 | 37.713 | 1.45E−05 |
| 740 | 51385 | *ZNF589* | 0.275 | 37.698 | 1.46E−05 |
| 741 | 25875 | *LETMD1* | 0.390 | 37.676 | 1.48E−05 |
| 742 | 79188 | *TMEM43* | −0.278 | 37.662 | 1.49E−05 |
| 743 | 283999 | *TMEM235* | 0.274 | 37.657 | 1.49E−05 |
| 744 | 11168 | *PSIP1* | 0.290 | 37.631 | 1.52E−05 |
| 745 | 56262 | *LRRC8A* | −0.286 | 37.606 | 1.53E−05 |
| 746 | 995 | *CDC25C* | 0.277 | 37.588 | 1.55E−05 |
| 747 | 10493 | *VAT1* | −0.272 | 37.569 | 1.56E−05 |
| 748 | 3268 | *AGFG2* | 0.285 | 37.553 | 1.58E−05 |
| 749 | 55276 | *PGM2* | −0.381 | 37.536 | 1.59E−05 |
| 750 | 64220 | *STRA6* | 0.303 | 37.527 | 1.60E−05 |
| 751 | 200942 | *KLHDC8B* | 0.324 | 37.514 | 1.61E−05 |
| 752 | 10319 | *LAMC3* | 0.297 | 37.513 | 1.61E−05 |
| 753 | 10980 | *COPS6* | 0.298 | 37.508 | 1.61E−05 |
| 754 | 55379 | *LRRC59* | −0.293 | 37.443 | 1.67E−05 |
| 755 | 7042 | *TGFB2* | −0.298 | 37.417 | 1.69E−05 |
| 756 | 5922 | *RASA2* | −0.282 | 37.404 | 1.70E−05 |
| 757 | 64764 | *CREB3L2* | −0.330 | 37.393 | 1.71E−05 |
| 758 | 2982 | *GUCY1A1* | 0.286 | 37.368 | 1.73E−05 |
| 759 | 114034 | *TOE1* | 0.269 | 37.353 | 1.75E−05 |
| 760 | 730051 | *ZNF814* | 0.281 | 37.326 | 1.77E−05 |
| 761 | 10121 | *ACTR1A* | −0.272 | 37.321 | 1.78E−05 |
| 762 | 6564 | *SLC15A1* | 0.284 | 37.318 | 1.78E−05 |
| 763 | 5525 | *PPP2R5A* | 0.265 | 37.312 | 1.78E−05 |
| 764 | 126129 | *CPT1C* | 0.307 | 37.300 | 1.79E−05 |
| 765 | 139818 | *DOCK11* | −0.351 | 37.293 | 1.80E−05 |
| 766 | 94240 | *EPSTI1* | −0.270 | 37.281 | 1.81E−05 |
| 767 | 684 | *BST2* | 0.274 | 37.257 | 1.84E−05 |
| 768 | 2222 | *FDFT1* | 0.314 | 37.216 | 1.87E−05 |
| 769 | 6943 | *TCF21* | 0.272 | 37.213 | 1.88E−05 |
| 770 | 79962 | *DNAJC22* | −0.294 | 37.169 | 1.92E−05 |
| 771 | 57136 | *APMAP* | −0.275 | 37.033 | 2.06E−05 |
| 772 | 55800 | *SCN3B* | −0.275 | 36.987 | 2.11E−05 |
| 773 | 23240 | *TMEM131L* | −0.303 | 36.935 | 2.16E−05 |
| 774 | 1050 | *CEBPA* | 0.330 | 36.884 | 2.22E−05 |
| 775 | 51283 | *BFAR* | −0.371 | 36.870 | 2.24E−05 |
| 776 | 6582 | *SLC22A2* | 0.284 | 36.863 | 2.25E−05 |
| 777 | 7139 | *TNNT2* | 0.284 | 36.862 | 2.25E−05 |
| 778 | 2735 | *GLI1* | 0.292 | 36.852 | 2.26E−05 |
| 779 | 10537 | *UBD* | −0.273 | 36.785 | 2.34E−05 |
| 780 | 151306 | *GPBAR1* | 0.306 | 36.695 | 2.45E−05 |
| 781 | 199731 | *CADM4* | −0.326 | 36.651 | 2.50E−05 |
| 782 | 60 | *ACTB* | −0.324 | 36.629 | 2.53E−05 |
| 783 | 7296 | *TXNRD1* | −0.368 | 36.610 | 2.56E−05 |
| 784 | 5139 | *PDE3A* | −0.313 | 36.600 | 2.57E−05 |
| 785 | 8644 | *AKR1C3* | 0.268 | 36.582 | 2.59E−05 |
| 786 | 1802 | *DPH2* | −0.273 | 36.569 | 2.61E−05 |
| 787 | 9557 | *CHD1L* | 0.283 | 36.568 | 2.61E−05 |
| 788 | 84889 | *SLC7A3* | 0.278 | 36.565 | 2.62E−05 |
| 789 | 48 | *ACO1* | −0.302 | 36.537 | 2.66E−05 |
| 790 | 3762 | *KCNJ5* | 0.347 | 36.526 | 2.67E−05 |
| 791 | 9570 | *GOSR2* | −0.310 | 36.525 | 2.67E−05 |

| | | | | | |
|---|---|---|---|---|---|
| 792 | 23324 | *MAN2B2* | −0.270 | 36.522 | 2.68E−05 |
| 793 | 79754 | *ASB13* | 0.266 | 36.510 | 2.69E−05 |
| 794 | 8091 | *HMGA2* | 0.288 | 36.421 | 2.82E−05 |
| 795 | 2676 | *GFRA3* | 0.272 | 36.416 | 2.83E−05 |
| 796 | 8633 | *UNC5C* | 0.287 | 36.399 | 2.85E−05 |
| 797 | 6565 | *SLC15A2* | −0.290 | 36.383 | 2.87E−05 |
| 798 | 377047 | *PRSS45P* | 0.272 | 36.331 | 2.95E−05 |
| 799 | 4293 | *MAP3K9* | −0.333 | 36.285 | 3.02E−05 |
| 800 | 7447 | *VSNL1* | 0.278 | 36.284 | 3.02E−05 |
| 801 | 345 | *APOC3* | 0.277 | 36.265 | 3.05E−05 |
| 802 | 201562 | *HACD2* | −0.321 | 36.212 | 3.14E−05 |
| 803 | 1635 | *DCTD* | −0.271 | 36.209 | 3.14E−05 |
| 804 | 3212 | *HOXB2* | 0.315 | 36.198 | 3.16E−05 |
| 805 | 90874 | *ZNF697* | −0.282 | 36.196 | 3.16E−05 |
| 806 | 23596 | *OPN3* | −0.283 | 36.116 | 3.30E−05 |
| 807 | 23023 | *TMCC1* | −0.289 | 36.102 | 3.32E−05 |
| 808 | 256435 | *ST6GALNAC3* | 0.281 | 36.086 | 3.35E−05 |
| 809 | 9304 | *SNORD22* | 0.283 | 36.038 | 3.43E−05 |
| 810 | 2805 | *GOT1* | 0.281 | 36.012 | 3.48E−05 |
| 811 | 6891 | *TAP2* | −0.273 | 35.996 | 3.50E−05 |
| 812 | 55625 | *ZDHHC7* | −0.296 | 35.992 | 3.51E−05 |
| 813 | 57447 | *NDRG2* | 0.293 | 35.978 | 3.54E−05 |
| 814 | 728464 | *METTL24* | 0.335 | 35.975 | 3.54E−05 |
| 815 | 59352 | *LGR6* | 0.274 | 35.974 | 3.54E−05 |
| 816 | 9212 | *AURKB* | 0.288 | 35.950 | 3.59E−05 |
| 817 | 257629 | *ANKS4B* | 0.297 | 35.935 | 3.62E−05 |
| 818 | 10391 | *CORO2B* | −0.276 | 35.889 | 3.70E−05 |
| 819 | 83877 | *TM2D2* | −0.273 | 35.881 | 3.72E−05 |
| 820 | 9464 | *HAND2* | 0.288 | 35.876 | 3.73E−05 |
| 821 | 3516 | *RBPJ* | 0.321 | 35.835 | 3.81E−05 |
| 822 | 56670 | *SUCNR1* | 0.276 | 35.833 | 3.81E−05 |
| 823 | 57553 | *MICAL3* | −0.312 | 35.821 | 3.83E−05 |
| 824 | 23607 | *CD2AP* | −0.361 | 35.811 | 3.85E−05 |
| 825 | 4781 | *NFIB* | 0.274 | 35.787 | 3.90E−05 |
| 826 | 8678 | *BECN1* | −0.383 | 35.768 | 3.94E−05 |
| 827 | 732 | *C8B* | 0.280 | 35.746 | 3.98E−05 |
| 828 | 79109 | *MAPKAP1* | −0.262 | 35.732 | 4.01E−05 |
| 829 | 25853 | *DCAF12* | −0.308 | 35.716 | 4.05E−05 |
| 830 | 84649 | *DGAT2* | 0.278 | 35.688 | 4.10E−05 |
| 831 | 9915 | *ARNT2* | −0.268 | 35.669 | 4.15E−05 |
| 832 | 80332 | *ADAM33* | 0.289 | 35.605 | 4.28E−05 |
| 833 | 7138 | *TNNT1* | 0.284 | 35.594 | 4.31E−05 |
| 834 | 81788 | *NUAK2* | −0.336 | 35.563 | 4.38E−05 |
| 835 | 51382 | *ATP6V1D* | −0.292 | 35.542 | 4.42E−05 |
| 836 | 55666 | *NPLOC4* | −0.297 | 35.530 | 4.45E−05 |
| 837 | 7408 | *VASP* | −0.392 | 35.495 | 4.53E−05 |
| 838 | 11342 | *RNF13* | −0.319 | 35.486 | 4.55E−05 |
| 839 | 79571 | *GCC1* | −0.387 | 35.474 | 4.58E−05 |
| 840 | 7247 | *TSN* | −0.305 | 35.464 | 4.61E−05 |
| 841 | 5837 | *PYGM* | 0.271 | 35.450 | 4.64E−05 |
| 842 | 668 | *FOXL2* | −0.289 | 35.418 | 4.71E−05 |
| 843 | 3671 | *ISLR* | 0.282 | 35.410 | 4.73E−05 |
| 844 | 55075 | *UACA* | −0.266 | 35.410 | 4.73E−05 |

| | | | | | |
|---|---|---|---|---|---|
| 845 | 6447 | *SCG5* | 0.271 | 35.389 | 4.79E−05 |
| 846 | 92126 | *DSEL* | −0.304 | 35.384 | 4.80E−05 |
| 847 | 54874 | *FNBP1L* | −0.320 | 35.375 | 4.82E−05 |
| 848 | 47 | *ACLY* | −0.359 | 35.343 | 4.90E−05 |
| 849 | 2887 | *GRB10* | −0.294 | 35.340 | 4.91E−05 |
| 850 | 3218 | *HOXB8* | 0.291 | 35.329 | 4.94E−05 |
| 851 | 83930 | *STARD3NL* | −0.288 | 35.326 | 4.94E−05 |
| 852 | 85364 | *ZCCHC3* | 0.312 | 35.322 | 4.95E−05 |
| 853 | 3688 | *ITGB1* | −0.366 | 35.296 | 5.02E−05 |
| 854 | 7798 | *LUZP1* | −0.369 | 35.284 | 5.05E−05 |
| 855 | 29980 | *DONSON* | 0.274 | 35.283 | 5.05E−05 |
| 856 | 6890 | *TAP1* | −0.274 | 35.256 | 5.12E−05 |
| 857 | 862 | *RUNX1T1* | 0.278 | 35.231 | 5.19E−05 |
| 858 | 2330 | *FMO5* | 0.286 | 35.225 | 5.21E−05 |
| 859 | 9423 | *NTN1* | −0.276 | 35.203 | 5.27E−05 |
| 860 | 57820 | *CCNB1IP1* | 0.270 | 35.194 | 5.29E−05 |
| 861 | 55041 | *PLEKHB2* | −0.381 | 35.184 | 5.32E−05 |
| 862 | 7132 | *TNFRSF1A* | −0.297 | 35.142 | 5.43E−05 |
| 863 | 8720 | *MBTPS1* | −0.297 | 35.130 | 5.47E−05 |
| 864 | 142683 | *ITLN2* | 0.267 | 35.096 | 5.56E−05 |
| 865 | 79154 | *DHRS11* | 0.276 | 35.079 | 5.61E−05 |
| 866 | 84002 | *B3GNT5* | −0.332 | 35.056 | 5.68E−05 |
| 867 | 79774 | *GRTP1* | 0.283 | 35.052 | 5.69E−05 |
| 868 | 27063 | *ANKRD1* | −0.266 | 35.037 | 5.73E−05 |
| 869 | 3795 | *KHK* | 0.254 | 35.036 | 5.74E−05 |
| 870 | 84904 | *ARHGEF39* | 0.284 | 34.991 | 5.87E−05 |
| 871 | 11340 | *EXOSC8* | 0.261 | 34.979 | 5.91E−05 |
| 872 | 5159 | *PDGFRB* | 0.279 | 34.928 | 6.07E−05 |
| 873 | 59344 | *ALOXE3* | −0.263 | 34.834 | 6.37E−05 |
| 874 | 55573 | *CDV3* | −0.358 | 34.832 | 6.37E−05 |
| 875 | 5538 | *PPT1* | 0.267 | 34.815 | 6.43E−05 |
| 876 | 80176 | *SPSB1* | −0.283 | 34.784 | 6.53E−05 |
| 877 | 8639 | *AOC3* | 0.261 | 34.710 | 6.78E−05 |
| 878 | 6430 | *SRSF5* | 0.307 | 34.699 | 6.82E−05 |
| 879 | 55917 | *CTTNBP2NL* | −0.342 | 34.681 | 6.88E−05 |
| 880 | 126661 | *CCDC163* | 0.265 | 34.668 | 6.93E−05 |
| 881 | 90134 | *KCNH7* | −0.275 | 34.649 | 7.00E−05 |
| 882 | 10605 | *PAIP1* | −0.262 | 34.646 | 7.01E−05 |
| 883 | 1997 | *ELF1* | −0.320 | 34.628 | 7.07E−05 |
| 884 | 51703 | *ACSL5* | 0.301 | 34.627 | 7.08E−05 |
| 885 | 8908 | *GYG2* | 0.293 | 34.626 | 7.08E−05 |
| 886 | 27239 | *GPR162* | 0.298 | 34.588 | 7.22E−05 |
| 887 | 387640 | *SKIDA1* | 0.261 | 34.586 | 7.23E−05 |
| 888 | 54930 | *HAUS4* | 0.257 | 34.574 | 7.27E−05 |
| 889 | 2978 | *GUCA1A* | 0.275 | 34.571 | 7.28E−05 |
| 890 | 57600 | *FNIP2* | −0.369 | 34.571 | 7.29E−05 |
| 891 | 5322 | *PLA2G5* | 0.264 | 34.570 | 7.29E−05 |
| 892 | 84695 | *LOXL3* | 0.287 | 34.568 | 7.30E−05 |
| 893 | 5493 | *PPL* | −0.274 | 34.557 | 7.34E−05 |
| 894 | 643236 | *TMEM72* | 0.269 | 34.550 | 7.36E−05 |
| 895 | 9936 | *CD302* | 0.264 | 34.547 | 7.38E−05 |
| 896 | 93974 | *ATP5IF1* | 0.353 | 34.537 | 7.41E−05 |
| 897 | 656 | *BMP8B* | −0.276 | 34.519 | 7.48E−05 |

| | | | | | |
|---|---|---|---|---|---|
| 898 | 113419 | *TEX261* | −0.266 | 34.506 | 7.53E−05 |
| 899 | 2184 | *FAH* | 0.275 | 34.484 | 7.62E−05 |
| 900 | 79794 | *C12orf49* | −0.273 | 34.438 | 7.80E−05 |
| 901 | 80228 | *ORAI2* | −0.268 | 34.431 | 7.83E−05 |
| 902 | 1.05E+08 | *LINC01767* | 0.262 | 34.398 | 7.96E−05 |
| 903 | 54838 | *WBP1L* | −0.303 | 34.386 | 8.01E−05 |
| 904 | 336 | *APOA2* | 0.278 | 34.384 | 8.02E−05 |
| 905 | 26190 | *FBXW2* | −0.314 | 34.371 | 8.07E−05 |
| 906 | 54680 | *ZNHIT6* | −0.314 | 34.351 | 8.16E−05 |
| 907 | 167410 | *LIX1* | 0.265 | 34.342 | 8.20E−05 |
| 908 | 27294 | *DHDH* | 0.295 | 34.302 | 8.37E−05 |
| 909 | 387758 | *FIBIN* | 0.271 | 34.288 | 8.42E−05 |
| 910 | 255743 | *NPNT* | −0.255 | 34.255 | 8.57E−05 |
| 911 | 3417 | *IDH1* | 0.294 | 34.254 | 8.57E−05 |
| 912 | 26511 | *CHIC2* | −0.288 | 34.240 | 8.64E−05 |
| 913 | 11252 | *PACSIN2* | −0.309 | 34.215 | 8.75E−05 |
| 914 | 2628 | *GATM* | 0.261 | 34.203 | 8.80E−05 |
| 915 | 10733 | *PLK4* | 0.288 | 34.151 | 9.04E−05 |
| 916 | 79849 | *PDZD3* | 0.264 | 34.127 | 9.15E−05 |
| 917 | 120376 | *COLCA2* | 0.256 | 34.060 | 9.47E−05 |
| 918 | 171024 | *SYNPO2* | 0.265 | 34.059 | 9.48E−05 |
| 919 | 84676 | *TRIM63* | 0.251 | 34.049 | 9.53E−05 |
| 920 | 3215 | *HOXB5* | 0.300 | 34.048 | 9.53E−05 |
| 921 | 55 | *ACP3* | 0.265 | 34.020 | 9.67E−05 |
| 922 | 159 | *ADSS2* | −0.307 | 34.020 | 9.67E−05 |
| 923 | 134429 | *STARD4* | 0.320 | 34.010 | 9.72E−05 |
| 924 | 344595 | *DUBR* | −0.281 | 33.973 | 9.91E−05 |
| 925 | 56300 | *IL36G* | 0.272 | 33.963 | 9.96E−05 |
| 926 | 1611 | *DAP* | −0.260 | 33.915 | 1.02E−04 |
| 927 | 23409 | *SIRT4* | 0.263 | 33.897 | 1.03E−04 |
| 928 | 79630 | *C1orf54* | 0.264 | 33.877 | 1.04E−04 |
| 929 | 6328 | *SCN3A* | −0.292 | 33.874 | 1.04E−04 |
| 930 | 1238 | *ACKR2* | −0.275 | 33.863 | 1.05E−04 |
| 931 | 25978 | *CHMP2B* | −0.373 | 33.851 | 1.05E−04 |
| 932 | 220134 | *SKA1* | 0.268 | 33.836 | 1.06E−04 |
| 933 | 10278 | *EFS* | 0.295 | 33.809 | 1.08E−04 |
| 934 | 84640 | *USP38* | −0.329 | 33.800 | 1.08E−04 |
| 935 | 528 | *ATP6V1C1* | −0.369 | 33.792 | 1.09E−04 |
| 936 | 55108 | *BSDC1* | −0.253 | 33.779 | 1.09E−04 |
| 937 | 57169 | *ZNFX1* | −0.294 | 33.758 | 1.11E−04 |
| 938 | 9666 | *DZIP3* | 0.308 | 33.753 | 1.11E−04 |
| 939 | 11215 | *AKAP11* | −0.326 | 33.708 | 1.14E−04 |
| 940 | 64093 | *SMOC1* | 0.279 | 33.708 | 1.14E−04 |
| 941 | 51090 | *PLLP* | 0.277 | 33.699 | 1.14E−04 |
| 942 | 2752 | *GLUL* | 0.268 | 33.694 | 1.14E−04 |
| 943 | 64208 | *POPDC3* | 0.260 | 33.693 | 1.14E−04 |
| 944 | 26160 | *IFT172* | 0.264 | 33.669 | 1.16E−04 |
| 945 | 2706 | *GJB2* | 0.274 | 33.577 | 1.21E−04 |
| 946 | 51719 | *CAB39* | −0.320 | 33.564 | 1.22E−04 |
| 947 | 401262 | *CRIP3* | 0.266 | 33.552 | 1.23E−04 |
| 948 | 4018 | *LPA* | 0.268 | 33.537 | 1.24E−04 |
| 949 | 8507 | *ENC1* | −0.266 | 33.519 | 1.25E−04 |
| 950 | 88 | *ACTN2* | 0.266 | 33.506 | 1.26E−04 |

| | | | | | |
|---|---|---|---|---|---|
| 951 | 1129 | *CHRM2* | 0.259 | 33.497 | 1.27E−04 |
| 952 | 55858 | *TMEM165* | −0.256 | 33.496 | 1.27E−04 |
| 953 | 11057 | *ABHD2* | −0.374 | 33.466 | 1.29E−04 |
| 954 | 214 | *ALCAM* | −0.358 | 33.455 | 1.29E−04 |
| 955 | 84148 | *KAT8* | 0.329 | 33.444 | 1.30E−04 |
| 956 | 7070 | *THY1* | 0.274 | 33.444 | 1.30E−04 |
| 957 | 254225 | *RNF169* | −0.334 | 33.427 | 1.31E−04 |
| 958 | 4817 | *NIT1* | 0.295 | 33.427 | 1.31E−04 |
| 959 | 27020 | *NPTN* | −0.294 | 33.392 | 1.34E−04 |
| 960 | 731 | *C8A* | 0.256 | 33.373 | 1.35E−04 |
| 961 | 4076 | *CAPRIN1* | −0.360 | 33.363 | 1.36E−04 |
| 962 | 2705 | *GJB1* | 0.275 | 33.337 | 1.37E−04 |
| 963 | 3216 | *HOXB6* | 0.287 | 33.330 | 1.38E−04 |
| 964 | 5781 | *PTPN11* | −0.382 | 33.306 | 1.40E−04 |
| 965 | 1E+08 | *ZNF587B* | 0.261 | 33.296 | 1.40E−04 |
| 966 | 9754 | *STARD8* | 0.252 | 33.268 | 1.42E−04 |
| 967 | 55107 | *ANO1* | 0.256 | 33.258 | 1.43E−04 |
| 968 | 114793 | *FMNL2* | −0.288 | 33.232 | 1.45E−04 |
| 969 | 445815 | *PALM2AKAP2* | −0.334 | 33.203 | 1.47E−04 |
| 970 | 29855 | *UBN1* | −0.295 | 33.175 | 1.49E−04 |
| 971 | 6405 | *SEMA3F* | 0.281 | 33.117 | 1.54E−04 |
| 972 | 4082 | *MARCKS* | −0.290 | 33.101 | 1.55E−04 |
| 973 | 8536 | *CAMK1* | 0.316 | 33.095 | 1.56E−04 |
| 974 | 8869 | *ST3GAL5* | 0.276 | 33.090 | 1.56E−04 |
| 975 | 11341 | *SCRG1* | 0.373 | 33.080 | 1.57E−04 |
| 976 | 9077 | *DIRAS3* | 0.264 | 33.071 | 1.57E−04 |
| 977 | 128338 | *DRAM2* | 0.263 | 33.060 | 1.58E−04 |
| 978 | 10049 | *DNAJB6* | −0.254 | 33.053 | 1.59E−04 |
| 979 | 7402 | *UTRN* | −0.390 | 33.022 | 1.62E−04 |
| 980 | 3837 | *KPNB1* | −0.343 | 33.019 | 1.62E−04 |
| 981 | 51704 | *GPRC5B* | −0.260 | 33.015 | 1.62E−04 |
| 982 | 9308 | *CD83* | 0.268 | 32.967 | 1.66E−04 |
| 983 | 92667 | *MGME1* | 0.263 | 32.962 | 1.67E−04 |
| 984 | 387921 | *NHLRC3* | 0.290 | 32.935 | 1.69E−04 |
| 985 | 666 | *BOK* | −0.340 | 32.930 | 1.69E−04 |
| 986 | 6372 | *CXCL6* | −0.248 | 32.871 | 1.75E−04 |
| 987 | 387882 | *C12orf75* | −0.273 | 32.870 | 1.75E−04 |
| 988 | 1428 | *CRYM* | 0.257 | 32.837 | 1.78E−04 |
| 989 | 23185 | *LARP4B* | −0.277 | 32.828 | 1.78E−04 |
| 990 | 1203 | *CLN5* | −0.284 | 32.823 | 1.79E−04 |
| 991 | 4751 | *NEK2* | 0.268 | 32.791 | 1.82E−04 |
| 992 | 147841 | *SPC24* | 0.261 | 32.784 | 1.83E−04 |
| 993 | 338094 | *FAM151A* | 0.260 | 32.770 | 1.84E−04 |
| 994 | 6599 | *SMARCC1* | −0.339 | 32.755 | 1.85E−04 |
| 995 | 8772 | *FADD* | −0.314 | 32.744 | 1.86E−04 |
| 996 | 130589 | *GALM* | 0.260 | 32.730 | 1.88E−04 |
| 997 | 3158 | *HMGCS2* | 0.258 | 32.717 | 1.89E−04 |
| 998 | 23002 | *DAAM1* | −0.413 | 32.652 | 1.95E−04 |
| 999 | 284716 | *RIMKLA* | −0.253 | 32.597 | 2.01E−04 |
| 1000 | 340348 | *TSPAN33* | 0.287 | 32.549 | 2.06E−04 |
| 1001 | 83988 | *NCALD* | 0.267 | 32.547 | 2.06E−04 |
| 1002 | 799 | *CALCR* | −0.253 | 32.543 | 2.07E−04 |
| 1003 | 3065 | *HDAC1* | 0.260 | 32.537 | 2.07E−04 |

| | | | | | |
|---|---|---|---|---|---|
| 1004 | 5327 | *PLAT* | 0.276 | 32.529 | 2.08E−04 |
| 1005 | 23089 | *PEG10* | −0.268 | 32.529 | 2.08E−04 |
| 1006 | 3091 | *HIF1A* | −0.296 | 32.485 | 2.13E−04 |
| 1007 | 5566 | *PRKACA* | 0.342 | 32.480 | 2.13E−04 |
| 1008 | 55974 | *SLC50A1* | 0.266 | 32.453 | 2.16E−04 |
| 1009 | 64411 | *ARAP3* | 0.269 | 32.446 | 2.17E−04 |
| 1010 | 8303 | *SNN* | −0.262 | 32.440 | 2.18E−04 |
| 1011 | 79650 | *USB1* | −0.295 | 32.429 | 2.19E−04 |
| 1012 | 7094 | *TLN1* | −0.257 | 32.418 | 2.20E−04 |
| 1013 | 23016 | *EXOSC7* | 0.260 | 32.404 | 2.22E−04 |
| 1014 | 161497 | *STRC* | 0.273 | 32.391 | 2.23E−04 |
| 1015 | 2305 | *FOXM1* | 0.265 | 32.376 | 2.25E−04 |
| 1016 | 835 | *CASP2* | −0.258 | 32.361 | 2.27E−04 |
| 1017 | 92906 | *HNRNPLL* | −0.260 | 32.354 | 2.28E−04 |
| 1018 | 9586 | *CREB5* | −0.273 | 32.340 | 2.29E−04 |
| 1019 | 409 | *ARRB2* | 0.271 | 32.322 | 2.31E−04 |
| 1020 | 79443 | *FYCO1* | −0.335 | 32.313 | 2.33E−04 |
| 1021 | 1E+08 | *LINC00173* | 0.257 | 32.311 | 2.33E−04 |
| 1022 | 158219 | *TTC39B* | 0.313 | 32.291 | 2.35E−04 |
| 1023 | 10085 | *EDIL3* | −0.277 | 32.287 | 2.36E−04 |
| 1024 | 5142 | *PDE4B* | 0.262 | 32.273 | 2.37E−04 |
| 1025 | 220441 | *RNF152* | 0.410 | 32.262 | 2.39E−04 |
| 1026 | 151313 | *FAHD2B* | 0.269 | 32.259 | 2.39E−04 |
| 1027 | 56033 | *BARX1* | −0.285 | 32.245 | 2.41E−04 |
| 1028 | 219654 | *ZCCHC24* | 0.264 | 32.239 | 2.42E−04 |
| 1029 | 55841 | *WWC3* | −0.263 | 32.238 | 2.42E−04 |
| 1030 | 56256 | *SERTAD4* | 0.269 | 32.236 | 2.42E−04 |
| 1031 | 55332 | *DRAM1* | 0.260 | 32.234 | 2.42E−04 |
| 1032 | 8773 | *SNAP23* | −0.273 | 32.220 | 2.44E−04 |
| 1033 | 9498 | *SLC4A8* | 0.261 | 32.205 | 2.46E−04 |
| 1034 | 116832 | *RPL39L* | 0.263 | 32.190 | 2.48E−04 |
| 1035 | 1.03E+08 | *LOC102724064* | 0.265 | 32.184 | 2.49E−04 |
| 1036 | 7879 | *RAB7A* | −0.270 | 32.173 | 2.50E−04 |
| 1037 | 7846 | *TUBA1A* | −0.276 | 32.163 | 2.51E−04 |
| 1038 | 23179 | *RGL1* | −0.307 | 32.116 | 2.57E−04 |
| 1039 | 144363 | *ETFRF1* | 0.282 | 32.093 | 2.61E−04 |
| 1040 | 84284 | *NTPCR* | 0.287 | 32.084 | 2.62E−04 |
| 1041 | 3485 | *IGFBP2* | −0.331 | 32.038 | 2.68E−04 |
| 1042 | 80381 | *CD276* | −0.280 | 32.036 | 2.68E−04 |
| 1043 | 5550 | *PREP* | −0.271 | 32.021 | 2.70E−04 |
| 1044 | 517 | *ATP5MC2* | 0.266 | 32.018 | 2.71E−04 |
| 1045 | 51250 | *MTRES1* | 0.250 | 32.014 | 2.71E−04 |
| 1046 | 80271 | *ITPKC* | −0.261 | 31.998 | 2.74E−04 |
| 1047 | 343637 | *RSPO4* | 0.271 | 31.994 | 2.74E−04 |
| 1048 | 84329 | *HVCN1* | 0.273 | 31.994 | 2.74E−04 |
| 1049 | 140680 | *C20orf96* | 0.288 | 31.985 | 2.75E−04 |
| 1050 | 57412 | *AS3MT* | 0.259 | 31.957 | 2.79E−04 |
| 1051 | 64172 | *OSGEPL1* | 0.256 | 31.957 | 2.79E−04 |
| 1052 | 129807 | *NEU4* | 0.325 | 31.946 | 2.81E−04 |
| 1053 | 83478 | *ARHGAP24* | −0.276 | 31.928 | 2.84E−04 |
| 1054 | 26027 | *ACOT11* | 0.465 | 31.923 | 2.84E−04 |
| 1055 | 10232 | *MSLN* | 0.297 | 31.909 | 2.86E−04 |
| 1056 | 79183 | *TTPAL* | −0.309 | 31.908 | 2.87E−04 |

| | | | | | |
|---|---|---|---|---|---|
| 1057 | 10627 | *MYL12A* | −0.256 | 31.894 | 2.89E−04 |
| 1058 | 23704 | *KCNE4* | 0.263 | 31.892 | 2.89E−04 |
| 1059 | 64399 | *HHIP* | 0.284 | 31.889 | 2.89E−04 |
| 1060 | 8106 | *PABPN1* | 0.464 | 31.814 | 3.01E−04 |
| 1061 | 57333 | *RCN3* | 0.288 | 31.794 | 3.04E−04 |
| 1062 | 2316 | *FLNA* | −0.262 | 31.769 | 3.08E−04 |
| 1063 | 2002 | *ELK1* | −0.311 | 31.768 | 3.08E−04 |
| 1064 | 10097 | *ACTR2* | −0.317 | 31.767 | 3.08E−04 |
| 1065 | 201161 | *CENPV* | 0.291 | 31.745 | 3.12E−04 |
| 1066 | 23370 | *ARHGEF18* | −0.298 | 31.705 | 3.18E−04 |
| 1067 | 51660 | *MPC1* | 0.279 | 31.637 | 3.29E−04 |
| 1068 | 114787 | *GPRIN1* | −0.263 | 31.634 | 3.30E−04 |
| 1069 | 79576 | *NKAP* | 0.323 | 31.617 | 3.33E−04 |
| 1070 | 375444 | *C5orf34* | 0.260 | 31.615 | 3.33E−04 |
| 1071 | 9411 | *ARHGAP29* | −0.313 | 31.600 | 3.36E−04 |
| 1072 | 55144 | *LRRC8D* | 0.270 | 31.596 | 3.36E−04 |
| 1073 | 5834 | *PYGB* | −0.275 | 31.593 | 3.37E−04 |
| 1074 | 282996 | *RBM20* | 0.261 | 31.581 | 3.39E−04 |
| 1075 | 10083 | *USH1C* | 0.269 | 31.578 | 3.40E−04 |
| 1076 | 23051 | *ZHX3* | −0.344 | 31.575 | 3.40E−04 |
| 1077 | 5074 | *PAWR* | −0.393 | 31.544 | 3.46E−04 |
| 1078 | 22846 | *VASH1* | 0.281 | 31.523 | 3.49E−04 |
| 1079 | 5155 | *PDGFB* | −0.289 | 31.519 | 3.50E−04 |
| 1080 | 8334 | *H2AC6* | −0.252 | 31.517 | 3.50E−04 |
| 1081 | 399474 | *TMEM200B* | 0.376 | 31.506 | 3.52E−04 |
| 1082 | 6539 | *SLC6A12* | 0.254 | 31.486 | 3.56E−04 |
| 1083 | 84918 | *LRP11* | −0.248 | 31.467 | 3.60E−04 |
| 1084 | 55795 | *PCID2* | 0.260 | 31.460 | 3.61E−04 |
| 1085 | 923 | *CD6* | 0.253 | 31.457 | 3.61E−04 |
| 1086 | 23386 | *NUDCD3* | −0.264 | 31.456 | 3.62E−04 |
| 1087 | 84263 | *HSDL2* | 0.284 | 31.453 | 3.62E−04 |
| 1088 | 5984 | *RFC4* | 0.266 | 31.452 | 3.62E−04 |
| 1089 | 23209 | *MLC1* | 0.257 | 31.447 | 3.63E−04 |
| 1090 | 80736 | *SLC44A4* | 0.251 | 31.416 | 3.69E−04 |
| 1091 | 129804 | *FBLN7* | 0.256 | 31.393 | 3.74E−04 |
| 1092 | 1.01E+08 | *LINC01356* | 0.245 | 31.349 | 3.82E−04 |
| 1093 | 57124 | *CD248* | 0.275 | 31.332 | 3.85E−04 |
| 1094 | 3957 | *LGALS2* | 0.258 | 31.331 | 3.86E−04 |
| 1095 | 10257 | *ABCC4* | −0.316 | 31.329 | 3.86E−04 |
| 1096 | 130132 | *RFTN2* | 0.333 | 31.327 | 3.87E−04 |
| 1097 | 55967 | *NDUFA12* | 0.264 | 31.289 | 3.94E−04 |
| 1098 | 115361 | *GBP4* | 0.256 | 31.289 | 3.94E−04 |
| 1099 | 284656 | *EPHA10* | −0.269 | 31.283 | 3.95E−04 |
| 1100 | 348801 | *LNP1* | 0.253 | 31.270 | 3.98E−04 |
| 1101 | 253639 | *ZNF620* | 0.251 | 31.248 | 4.03E−04 |
| 1102 | 7468 | *NSD2* | 0.280 | 31.244 | 4.03E−04 |
| 1103 | 5576 | *PRKAR2A* | −0.374 | 31.226 | 4.07E−04 |
| 1104 | 22906 | *TRAK1* | −0.256 | 31.202 | 4.12E−04 |
| 1105 | 90550 | *MCU* | −0.271 | 31.197 | 4.13E−04 |
| 1106 | 7371 | *UCK2* | −0.248 | 31.196 | 4.13E−04 |
| 1107 | 7010 | *TEK* | 0.261 | 31.183 | 4.16E−04 |
| 1108 | 130872 | *AHSA2P* | 0.265 | 31.164 | 4.20E−04 |
| 1109 | 51560 | *RAB6B* | −0.252 | 31.146 | 4.24E−04 |

| | | | | | |
|---|---|---|---|---|---|
| 1110 | 6503 | *SLA* | 0.245 | 31.141 | 4.25E−04 |
| 1111 | 2203 | *FBP1* | 0.259 | 31.136 | 4.26E−04 |
| 1112 | 5972 | *REN* | 0.253 | 31.113 | 4.32E−04 |
| 1113 | 55082 | *ARGLU1* | 0.271 | 31.103 | 4.34E−04 |
| 1114 | 1370 | *CPN2* | 0.246 | 31.095 | 4.36E−04 |
| 1115 | 4724 | *NDUFS4* | 0.294 | 31.093 | 4.36E−04 |
| 1116 | 8835 | *SOCS2* | −0.271 | 31.084 | 4.38E−04 |
| 1117 | 1350 | *COX7C* | 0.258 | 31.078 | 4.39E−04 |
| 1118 | 63898 | *SH2D4A* | −0.290 | 31.072 | 4.41E−04 |
| 1119 | 4714 | *NDUFB8* | 0.285 | 31.063 | 4.43E−04 |
| 1120 | 5464 | *PPA1* | −0.279 | 31.030 | 4.50E−04 |
| 1121 | 8829 | *NRP1* | −0.281 | 31.023 | 4.52E−04 |
| 1122 | 221692 | *PHACTR1* | 0.316 | 31.005 | 4.56E−04 |
| 1123 | 112487 | *DTD2* | −0.258 | 30.981 | 4.62E−04 |
| 1124 | 203 | *AK1* | 0.274 | 30.980 | 4.62E−04 |
| 1125 | 7328 | *UBE2H* | −0.277 | 30.936 | 4.73E−04 |
| 1126 | 10973 | *ASCC3* | −0.362 | 30.927 | 4.75E−04 |
| 1127 | 90736 | *FAM104B* | 0.246 | 30.908 | 4.79E−04 |
| 1128 | 4921 | *DDR2* | 0.262 | 30.891 | 4.84E−04 |
| 1129 | 10643 | *IGF2BP3* | −0.363 | 30.883 | 4.86E−04 |
| 1130 | 5740 | *PTGIS* | 0.257 | 30.871 | 4.89E−04 |
| 1131 | 4060 | *LUM* | 0.267 | 30.851 | 4.94E−04 |
| 1132 | 10864 | *SLC22A7* | 0.242 | 30.838 | 4.97E−04 |
| 1133 | 64965 | *MRPS9* | 0.255 | 30.830 | 4.99E−04 |
| 1134 | 51106 | *TFB1M* | 0.263 | 30.816 | 5.03E−04 |
| 1135 | 140876 | *RIPOR3* | 0.258 | 30.808 | 5.05E−04 |
| 1136 | 10521 | *DDX17* | 0.243 | 30.776 | 5.13E−04 |
| 1137 | 23514 | *SPIDR* | 0.253 | 30.764 | 5.16E−04 |
| 1138 | 10045 | *SH2D3A* | −0.312 | 30.753 | 5.19E−04 |
| 1139 | 6709 | *SPTAN1* | −0.249 | 30.738 | 5.23E−04 |
| 1140 | 116028 | *RMI2* | 0.270 | 30.732 | 5.25E−04 |
| 1141 | 162494 | *RHBDL3* | −0.255 | 30.726 | 5.27E−04 |
| 1142 | 115330 | *GPR146* | 0.275 | 30.643 | 5.50E−04 |
| 1143 | 114794 | *ELFN2* | −0.260 | 30.633 | 5.53E−04 |
| 1144 | 8573 | *CASK* | −0.303 | 30.632 | 5.53E−04 |
| 1145 | 64344 | *HIF3A* | 0.276 | 30.619 | 5.57E−04 |
| 1146 | 8742 | *TNFSF12* | 0.284 | 30.616 | 5.57E−04 |
| 1147 | 9619 | *ABCG1* | −0.247 | 30.607 | 5.60E−04 |
| 1148 | 8438 | *RAD54L* | 0.255 | 30.606 | 5.60E−04 |
| 1149 | 4248 | *MGAT3* | 0.280 | 30.590 | 5.65E−04 |
| 1150 | 93166 | *PRDM6* | 0.259 | 30.574 | 5.70E−04 |
| 1151 | 57733 | *GBA3* | 0.259 | 30.567 | 5.72E−04 |
| 1152 | 283659 | *PRTG* | 0.275 | 30.561 | 5.74E−04 |
| 1153 | 284992 | *CCDC150* | 0.255 | 30.518 | 5.86E−04 |
| 1154 | 51552 | *RAB14* | −0.359 | 30.493 | 5.94E−04 |
| 1155 | 25921 | *ZDHHC5* | −0.283 | 30.469 | 6.01E−04 |
| 1156 | 10403 | *NDC80* | 0.257 | 30.464 | 6.03E−04 |
| 1157 | 87 | *ACTN1* | −0.252 | 30.461 | 6.04E−04 |
| 1158 | 120892 | *LRRK2* | 0.257 | 30.448 | 6.08E−04 |
| 1159 | 51755 | *CDK12* | −0.444 | 30.431 | 6.13E−04 |
| 1160 | 8546 | *AP3B1* | −0.390 | 30.373 | 6.32E−04 |
| 1161 | 11105 | *PRDM7* | 0.247 | 30.371 | 6.33E−04 |
| 1162 | 283 | *ANG* | 0.263 | 30.325 | 6.48E−04 |

| | | | | | |
|---|---|---|---|---|---|
| 1163 | 2766 | *GMPR* | 0.247 | 30.320 | 6.49E−04 |
| 1164 | 9324 | *HMGN3* | 0.252 | 30.268 | 6.67E−04 |
| 1165 | 10507 | *SEMA4D* | 0.245 | 30.230 | 6.80E−04 |
| 1166 | 30850 | *CDR2L* | −0.322 | 30.223 | 6.83E−04 |
| 1167 | 1033 | *CDKN3* | 0.252 | 30.215 | 6.85E−04 |
| 1168 | 138151 | *NACC2* | −0.275 | 30.210 | 6.87E−04 |
| 1169 | 83607 | *AMMECR1L* | −0.324 | 30.198 | 6.91E−04 |
| 1170 | 158135 | *TTLL11* | −0.250 | 30.191 | 6.94E−04 |
| 1171 | 85315 | *PAQR8* | 0.264 | 30.191 | 6.94E−04 |
| 1172 | 65123 | *INTS3* | 0.260 | 30.168 | 7.02E−04 |
| 1173 | 1.05E+08 | *LOC105379252* | 0.285 | 30.167 | 7.03E−04 |
| 1174 | 8148 | *TAF15* | 0.247 | 30.164 | 7.04E−04 |
| 1175 | 389119 | *INKA1* | 0.273 | 30.123 | 7.19E−04 |
| 1176 | 1825 | *DSC3* | 0.256 | 30.111 | 7.23E−04 |
| 1177 | 3697 | *ITIH1* | 0.253 | 30.100 | 7.27E−04 |
| 1178 | 3836 | *KPNA1* | −0.347 | 30.099 | 7.28E−04 |
| 1179 | 55765 | *INAVA* | −0.266 | 30.095 | 7.29E−04 |
| 1180 | 1315 | *COPB1* | −0.350 | 30.091 | 7.31E−04 |
| 1181 | 79370 | *BCL2L14* | 0.244 | 30.064 | 7.41E−04 |
| 1182 | 285148 | *IAH1* | 0.266 | 30.059 | 7.43E−04 |
| 1183 | 28982 | *FLVCR1* | 0.260 | 30.040 | 7.50E−04 |
| 1184 | 1E+08 | *FAR2P2* | 0.255 | 30.032 | 7.53E−04 |
| 1185 | 81566 | *CSRNP2* | −0.270 | 30.029 | 7.54E−04 |
| 1186 | 79148 | *MMP28* | 0.275 | 30.000 | 7.66E−04 |
| 1187 | 22864 | *R3HDM2* | 0.253 | 29.992 | 7.69E−04 |
| 1188 | 57555 | *NLGN2* | 0.379 | 29.979 | 7.74E−04 |
| 1189 | 1.01E+08 | *SMLR1* | 0.263 | 29.970 | 7.78E−04 |
| 1190 | 374977 | *MROH7* | 0.276 | 29.963 | 7.80E−04 |
| 1191 | 143 | *PARP4* | −0.369 | 29.955 | 7.84E−04 |
| 1192 | 25890 | *ABI3BP* | 0.239 | 29.947 | 7.87E−04 |
| 1193 | 55773 | *TBC1D23* | −0.333 | 29.939 | 7.90E−04 |
| 1194 | 10240 | *MRPS31* | 0.270 | 29.937 | 7.91E−04 |
| 1195 | 5734 | *PTGER4* | −0.249 | 29.913 | 8.01E−04 |
| 1196 | 7135 | *TNNI1* | 0.255 | 29.906 | 8.04E−04 |
| 1197 | 1.05E+08 | *LINC02609* | 0.299 | 29.895 | 8.09E−04 |
| 1198 | 5502 | *PPP1R1A* | 0.261 | 29.892 | 8.10E−04 |
| 1199 | 247 | *ALOX15B* | 0.268 | 29.849 | 8.28E−04 |
| 1200 | 5660 | *PSAP* | −0.276 | 29.824 | 8.38E−04 |
| 1201 | 26578 | *OSTF1* | −0.250 | 29.813 | 8.44E−04 |
| 1202 | 2619 | *GAS1* | 0.270 | 29.778 | 8.59E−04 |
| 1203 | 89866 | *SEC16B* | 0.249 | 29.777 | 8.59E−04 |
| 1204 | 7188 | *TRAF5* | 0.268 | 29.768 | 8.63E−04 |
| 1205 | 115557 | *ARHGEF25* | 0.292 | 29.767 | 8.64E−04 |
| 1206 | 1E+08 | *PLA2G4B* | 0.351 | 29.762 | 8.66E−04 |
| 1207 | 143686 | *SESN3* | −0.284 | 29.745 | 8.73E−04 |
| 1208 | 284439 | *SLC25A42* | 0.279 | 29.742 | 8.75E−04 |
| 1209 | 122622 | *ADSS1* | 0.262 | 29.737 | 8.77E−04 |
| 1210 | 10125 | *RASGRP1* | −0.267 | 29.734 | 8.78E−04 |
| 1211 | 51474 | *LIMA1* | −0.358 | 29.721 | 8.84E−04 |
| 1212 | 9768 | *PCLAF* | 0.258 | 29.715 | 8.87E−04 |
| 1213 | 5451 | *POU2F1* | 0.255 | 29.692 | 8.98E−04 |
| 1214 | 1259 | *CNGA1* | 0.239 | 29.691 | 8.98E−04 |
| 1215 | 3383 | *ICAM1* | −0.247 | 29.668 | 9.09E−04 |

| | | | | | |
|---|---|---|---|---|---|
| 1216 | 57687 | *VAT1L* | 0.263 | 29.647 | 9.19E−04 |
| 1217 | 1562 | *CYP2C18* | 0.241 | 29.640 | 9.22E−04 |
| 1218 | 79884 | *MAP9* | −0.385 | 29.636 | 9.24E−04 |
| 1219 | 8029 | *CUBN* | 0.249 | 29.609 | 9.37E−04 |
| 1220 | 5985 | *RFC5* | 0.251 | 29.601 | 9.41E−04 |
| 1221 | 30061 | *SLC40A1* | −0.276 | 29.588 | 9.47E−04 |
| 1222 | 147968 | *CAPN12* | 0.277 | 29.579 | 9.52E−04 |
| 1223 | 5739 | *PTGIR* | 0.252 | 29.573 | 9.54E−04 |
| 1224 | 219736 | *STOX1* | 0.249 | 29.536 | 9.73E−04 |
| 1225 | 51760 | *SYT17* | −0.259 | 29.532 | 9.75E−04 |
| 1226 | 9448 | *MAP4K4* | −0.288 | 29.531 | 9.75E−04 |
| 1227 | 81624 | *DIAPH3* | −0.316 | 29.500 | 9.91E−04 |
| 1228 | 65267 | *WNK3* | 0.258 | 29.500 | 9.91E−04 |
| 1229 | 4194 | *MDM4* | 0.257 | 29.495 | 9.94E−04 |
