# Supplementary material for: Transcriptomic Architecture of Metabolic Dysfunction-Associated Steatotic Liver Disease (MASLD) Risk in Mexican Americans
Source: Cells. 2026 Sep 1;15(17):1592. doi: 10.3390/cells15171592 (PMC13565466; doi:10.3390/cells15171592)
Supplement: Supplementary file 1 [file cells-15-01592-s001.zip › Supplementary Table S1.pdf]

Supplementary Table S1: Genes whose expression was significantly associated with baseline hepatic steatosis measures.

S. No.	Entrez ID	Gene Symbol	β	β (χ^2)	Bonferroni-corrected *p*-value
1	10893	*MMP24*	−0.431	108.700	3.35E−21
2	71	*ACTG1*	−0.439	103.128	5.57E−20
3	9052	*GPRC5A*	−0.432	101.811	1.08E−19
4	4088	*SMAD3*	−0.440	96.455	1.62E−18
5	27242	*TNFRSF21*	−0.418	96.436	1.63E−18
6	4627	*MYH9*	−0.399	86.527	2.44E−16
7	9948	*WDR1*	−0.407	86.497	2.48E−16
8	8654	*PDE5A*	−0.410	86.250	2.81E−16
9	26136	*TES*	−0.404	84.735	6.05E−16
10	3927	*LASP1*	−0.387	83.859	9.42E−16
11	30844	*EHD4*	−0.405	80.902	4.20E−15
12	3856	*KRT8*	−0.442	80.029	6.54E−15
13	386617	*KCTD8*	−0.403	79.549	8.34E−15
14	338645	*LUZP2*	−0.398	78.778	1.23E−14
15	80307	*FER1L4*	0.416	78.658	1.31E−14
16	7286	*TUFT1*	−0.390	78.608	1.34E−14
17	79669	*C3orf52*	−0.424	78.447	1.46E−14
18	835	*CASP2*	−0.396	78.434	1.47E−14
19	1286	*COL4A4*	−0.416	78.097	1.74E−14
20	9124	*PDLIM1*	−0.382	77.412	2.46E−14
21	10381	*TUBB3*	−0.435	77.297	2.61E−14
22	649	*BMP1*	−0.403	77.099	2.88E−14
23	3340	*NDST1*	−0.423	76.110	4.76E−14
24	2051	*EPHB6*	−0.426	76.039	4.93E−14
25	3855	*KRT7*	−0.395	76.031	4.95E−14
26	8435	*SOAT2*	0.387	75.951	5.15E−14
27	3694	*ITGB6*	−0.405	75.497	6.49E−14
28	3875	*KRT18*	−0.399	75.464	6.59E−14
29	2053	*EPHX2*	0.377	74.892	8.81E−14
30	5648	*MASP1*	0.376	74.884	8.85E−14
31	6578	*SLCO2A1*	−0.376	73.271	2.00E−13
32	622	*BDH1*	0.379	72.962	2.34E−13
33	11098	*PRSS23*	−0.379	72.436	3.06E−13
34	9074	*CLDN6*	−0.395	72.152	3.53E−13
35	8128	*ST8SIA2*	−0.372	71.765	4.30E−13
36	3918	*LAMC2*	−0.433	71.351	5.30E−13
37	51062	*ATL1*	−0.384	71.209	5.69E−13
38	8407	*TAGLN2*	−0.397	70.533	8.02E−13
39	116372	*LYPD1*	−0.445	69.194	1.58E−12
40	57504	*MTA3*	0.375	69.077	1.68E−12
41	10072	*DPP3*	−0.405	68.989	1.76E−12
42	10231	*RCAN2*	−0.366	68.256	2.55E−12
43	5228	*PGF*	0.368	68.221	2.59E−12
44	285761	*DCBLD1*	−0.471	68.138	2.70E−12
45	27319	*BHLHE22*	−0.363	68.087	2.77E−12
46	57476	*GRAMD1B*	0.372	66.912	5.03E−12
47	272	*AMPD3*	−0.401	66.696	5.61E−12
48	10840	*ALDH1L1*	0.362	66.323	6.78E−12
49	3797	*KIF3C*	−0.366	66.276	6.95E−12

50	23313	*KIAA0930*	−0.381	66.172	7.32E−12
51	144132	*DNHD1*	0.389	65.910	8.37E−12
52	1363	*CPE*	−0.379	65.758	9.04E−12
53	7126	*TNFAIP1*	−0.375	65.081	1.27E−11
54	25893	*TRIM58*	−0.361	64.763	1.50E−11
55	8796	*SCEL*	−0.367	64.757	1.50E−11
56	4643	*MYO1E*	−0.403	64.754	1.50E−11
57	8802	*SUCLG1*	0.359	64.668	1.57E−11
58	6575	*SLC20A2*	−0.414	64.514	1.70E−11
59	2823	*GPM6A*	−0.380	64.438	1.77E−11
60	29995	*LMCD1*	−0.369	64.403	1.80E−11
61	84280	*BTBD10*	−0.441	64.125	2.07E−11
62	55833	*UBAP2*	−0.382	64.066	2.13E−11
63	1889	*ECE1*	−0.354	64.041	2.16E−11
64	26353	*HSPB8*	−0.362	63.845	2.39E−11
65	754	*PTTG1IP*	−0.383	63.802	2.44E−11
66	5210	*PFKFB4*	0.353	63.762	2.49E−11
67	1837	*DTNA*	−0.358	63.709	2.56E−11
68	7056	*THBD*	−0.348	63.612	2.68E−11
69	123920	*CMTM3*	−0.484	63.433	2.94E−11
70	33	*ACADL*	−0.344	62.968	3.72E−11
71	55915	*LANCL2*	−0.355	62.818	4.02E−11
72	432	*ASGR1*	0.385	62.639	4.40E−11
73	50859	*SPOCK3*	−0.357	62.570	4.56E−11
74	152816	*ODAPH*	−0.358	62.566	4.57E−11
75	4688	*NCF2*	−0.353	62.231	5.41E−11
76	5021	*OXTR*	−0.364	62.181	5.55E−11
77	200150	*PLD5*	−0.353	62.036	5.98E−11
78	286887	*KRT6C*	−0.359	61.997	6.10E−11
79	170954	*PPP1R18*	−0.399	61.908	6.38E−11
80	5638	*PRRG1*	−0.422	61.689	7.13E−11
81	64420	*SUSD1*	−0.431	61.458	8.02E−11
82	374378	*GALNT18*	−0.380	61.292	8.72E−11
83	9537	*TP53I11*	−0.383	61.105	9.59E−11
84	26355	*FAM162A*	0.349	60.981	1.02E−10
85	8842	*PROM1*	−0.448	60.813	1.11E−10
86	5530	*PPP3CA*	−0.413	60.705	1.18E−10
87	54751	*FBLIM1*	−0.418	60.541	1.28E−10
88	3853	*KRT6A*	−0.367	60.476	1.32E−10
89	7431	*VIM*	−0.399	60.385	1.38E−10
90	54843	*SYTL2*	−0.360	60.351	1.41E−10
91	6920	*TCEA3*	0.343	60.130	1.57E−10
92	9620	*CELSR1*	−0.350	60.075	1.62E−10
93	6335	*SCN9A*	−0.353	60.062	1.63E−10
94	824	*CAPN2*	−0.374	59.798	1.86E−10
95	81619	*TSPAN14*	−0.339	59.735	1.92E−10
96	3786	*KCNQ3*	−0.366	59.566	2.10E−10
97	5016	*OVGP1*	0.347	59.558	2.10E−10
98	3720	*JARID2*	0.364	59.330	2.36E−10
99	10397	*NDRG1*	0.342	59.305	2.39E−10
100	23286	*WWC1*	−0.367	59.226	2.49E−10
101	5324	*PLAG1*	0.366	58.636	3.36E−10
102	2555	*GABRA2*	−0.371	58.205	4.19E−10

103	647024	*C6orf132*	−0.343	58.185	4.23E−10
104	283635	*FAM177A1*	−0.350	58.127	4.36E−10
105	1124	*CHN2*	0.345	58.052	4.52E−10
106	2168	*FABP1*	0.346	58.035	4.56E−10
107	64759	*TNS3*	−0.424	58.023	4.59E−10
108	25816	*TNFAIP8*	−0.350	57.919	4.84E−10
109	51317	*PHF21A*	0.367	57.814	5.11E−10
110	5912	*RAP2B*	−0.361	57.771	5.22E−10
111	8482	*SEMA7A*	−0.350	57.535	5.89E−10
112	3659	*IRF1*	−0.346	57.388	6.34E−10
113	3675	*ITGA3*	−0.336	57.309	6.60E−10
114	8291	*DYSF*	−0.331	57.305	6.62E−10
115	25840	*METTL7A*	0.332	57.222	6.90E−10
116	25791	*NGEF*	0.360	57.215	6.93E−10
117	56938	*ARNTL2*	−0.379	57.183	7.04E−10
118	203111	*ERICH5*	−0.336	57.170	7.09E−10
119	9991	*PTBP3*	−0.442	57.080	7.42E−10
120	51330	*TNFRSF12A*	−0.522	56.939	7.97E−10
121	6876	*TAGLN*	−0.359	56.934	7.99E−10
122	6274	*S100A3*	−0.341	56.761	8.72E−10
123	25786	*DGCR11*	−0.344	56.676	9.11E−10
124	1268	*CNR1*	−0.334	56.644	9.26E−10
125	4615	*MYD88*	−0.348	56.594	9.50E−10
126	3662	*IRF4*	−0.336	56.578	9.57E−10
127	51312	*SLC25A37*	0.365	56.472	1.01E−09
128	1807	*DPYS*	0.346	56.390	1.05E−09
129	7905	*REEP5*	−0.372	56.336	1.08E−09
130	1264	*CNN1*	−0.385	56.168	1.18E−09
131	3294	*HSD17B2*	0.360	56.136	1.20E−09
132	9697	*TRAM2*	−0.409	56.102	1.22E−09
133	9540	*TP53I3*	−0.335	55.841	1.39E−09
134	9468	*PCYT1B*	0.341	55.710	1.49E−09
135	11259	*FILIP1L*	−0.361	55.643	1.54E−09
136	254427	*PROSER2*	−0.430	55.527	1.63E−09
137	2317	*FLNB*	−0.369	55.465	1.69E−09
138	1004	*CDH6*	−0.337	55.301	1.83E−09
139	304	*ANXA2P2*	−0.356	55.183	1.95E−09
140	283209	*PGM2L1*	−0.388	55.144	1.99E−09
141	55349	*CHDH*	0.379	55.015	2.12E−09
142	55315	*SLC29A3*	−0.347	54.814	2.35E−09
143	79695	*GALNT12*	0.334	54.766	2.41E−09
144	5770	*PTPN1*	−0.360	54.766	2.41E−09
145	6271	*S100A1*	0.346	54.721	2.46E−09
146	7852	*CXCR4*	−0.334	54.520	2.73E−09
147	4747	*NEFL*	−0.341	54.368	2.95E−09
148	94081	*SFXN1*	0.371	54.351	2.97E−09
149	23108	*RAP1GAP2*	−0.332	54.329	3.01E−09
150	60	*ACTB*	−0.435	54.324	3.01E−09
151	202018	*TAPT1*	−0.355	54.224	3.17E−09
152	302	*ANXA2*	−0.332	54.200	3.21E−09
153	3398	*ID2*	0.365	54.189	3.23E−09
154	115353	*LRRC42*	−0.390	54.173	3.25E−09
155	165257	*C1QL2*	−0.372	54.105	3.37E−09

156	6404	*SELPLG*	−0.366	53.976	3.60E−09
157	1030	*CDKN2B*	−0.340	53.942	3.66E−09
158	57591	*MRTFA*	−0.406	53.849	3.84E−09
159	8516	*ITGA8*	0.343	53.797	3.94E−09
160	2746	*GLUD1*	0.347	53.779	3.98E−09
161	54930	*HAUS4*	0.326	53.644	4.26E−09
162	10808	*HSPH1*	−0.385	53.470	4.65E−09
163	10602	*CDC42EP3*	−0.352	53.372	4.89E−09
164	9240	*PNMA1*	−0.356	52.908	6.19E−09
165	80256	*FAM214B*	−0.329	52.421	7.94E−09
166	64859	*NABP1*	−0.339	52.417	7.95E−09
167	230	*ALDOC*	0.322	52.402	8.02E−09
168	4645	*MYO5B*	−0.378	52.340	8.27E−09
169	6397	*SEC14L1*	−0.330	52.232	8.74E−09
170	2590	*GALNT2*	−0.322	52.226	8.77E−09
171	10096	*ACTR3*	−0.387	52.129	9.21E−09
172	57194	*ATP10A*	−0.323	51.967	1.00E−08
173	60312	*AFAP1*	−0.343	51.960	1.00E−08
174	55770	*EXOC2*	−0.384	51.883	1.04E−08
175	55741	*EDEM2*	−0.329	51.856	1.06E−08
176	57579	*FAM135A*	−0.384	51.740	1.12E−08
177	1495	*CTNNA1*	−0.414	51.706	1.14E−08
178	1001	*CDH3*	−0.331	51.704	1.14E−08
179	83877	*TM2D2*	−0.355	51.663	1.17E−08
180	157922	*CAMSAP1*	−0.388	51.657	1.17E−08
181	3983	*ABLIM1*	−0.354	51.599	1.21E−08
182	3835	*KIF22*	0.351	51.587	1.21E−08
183	23276	*KLHL18*	−0.332	51.583	1.22E−08
184	7360	*UGP2*	−0.400	51.510	1.26E−08
185	7532	*YWHAG*	−0.358	51.472	1.29E−08
186	2013	*EMP2*	0.330	51.445	1.30E−08
187	80114	*BICC1*	−0.373	51.334	1.38E−08
188	254863	*TMEM256*	0.437	51.223	1.46E−08
189	120114	*FAT3*	−0.347	51.064	1.58E−08
190	10894	*LYVE1*	−0.332	51.009	1.63E−08
191	23233	*EXOC6B*	−0.399	50.852	1.77E−08
192	10487	*CAP1*	−0.386	50.850	1.77E−08
193	326625	*MMAB*	0.329	50.800	1.81E−08
194	2202	*EFEMP1*	−0.352	50.798	1.81E−08
195	9528	*TMEM59*	−0.401	50.772	1.84E−08
196	898	*CCNE1*	0.325	50.731	1.88E−08
197	552	*AVPR1A*	−0.410	50.655	1.95E−08
198	9644	*SH3PXD2A*	0.357	50.512	2.10E−08
199	84617	*TUBB6*	−0.360	50.500	2.11E−08
200	56675	*NRIP3*	−0.338	50.426	2.19E−08
201	139065	*SLITRK4*	−0.321	50.412	2.21E−08
202	53836	*GPR87*	−0.349	50.391	2.23E−08
203	4329	*ALDH6A1*	0.324	50.361	2.27E−08
204	4485	*MST1*	0.348	50.298	2.34E−08
205	6774	*STAT3*	−0.413	50.126	2.56E−08
206	122622	*ADSS1*	0.372	50.122	2.56E−08
207	23446	*SLC44A1*	0.437	50.088	2.61E−08
208	79071	*ELOVL6*	0.329	49.996	2.73E−08

209	2766	*GMPR*	0.325	49.996	2.73E−08
210	3985	*LIMK2*	−0.367	49.957	2.79E−08
211	7204	*TRIO*	−0.311	49.897	2.87E−08
212	3854	*KRT6B*	−0.329	49.818	2.99E−08
213	2150	*F2RL1*	−0.331	49.750	3.10E−08
214	79837	*PIP4K2C*	−0.360	49.687	3.20E−08
215	51208	*CLDN18*	0.327	49.637	3.28E−08
216	445	*ASS1*	0.339	49.624	3.30E−08
217	10644	*IGF2BP2*	0.332	49.614	3.32E−08
218	9520	*NPEPPS*	−0.487	49.614	3.32E−08
219	50507	*NOX4*	−0.329	49.495	3.52E−08
220	1.02E+08	*PCAT14*	0.337	49.474	3.56E−08
221	79650	*USB1*	−0.346	49.439	3.63E−08
222	55347	*ABHD10*	0.360	49.412	3.68E−08
223	55298	*RNF121*	−0.342	49.361	3.77E−08
224	10045	*SH2D3A*	−0.396	49.351	3.79E−08
225	3590	*IL11RA*	0.346	49.314	3.87E−08
226	22	*ABCB7*	0.374	49.224	4.05E−08
227	2256	*FGF11*	0.322	49.127	4.25E−08
228	57142	*RTN4*	−0.343	49.002	4.53E−08
229	5089	*PBX2*	0.338	48.901	4.77E−08
230	8497	*PPFIA4*	0.323	48.837	4.93E−08
231	5155	*PDGFB*	−0.374	48.811	5.00E−08
232	7408	*VASP*	−0.479	48.775	5.09E−08
233	29881	*NPC1L1*	0.329	48.767	5.11E−08
234	51306	*FAM13B*	−0.450	48.666	5.38E−08
235	56262	*LRRC8A*	−0.330	48.580	5.62E−08
236	27233	*SULT1C4*	0.329	48.525	5.78E−08
237	9645	*MICAL2*	−0.369	48.508	5.83E−08
238	64061	*TSPYL2*	0.324	48.459	5.98E−08
239	414241	*SHLD2P1*	0.335	48.369	6.26E−08
240	23670	*CEMIP2*	−0.423	48.353	6.31E−08
241	26020	*LRP10*	−0.350	48.345	6.34E−08
242	27122	*DKK3*	−0.320	48.245	6.67E−08
243	4773	*NFATC2*	0.313	48.226	6.73E−08
244	1370	*CPN2*	0.330	48.212	6.78E−08
245	93145	*OLFM2*	−0.419	48.156	6.98E−08
246	3898	*LAD1*	−0.327	48.080	7.25E−08
247	3097	*HIVEP2*	−0.382	48.043	7.39E−08
248	51704	*GPRC5B*	−0.323	47.992	7.59E−08
249	4351	*MPI*	0.313	47.972	7.66E−08
250	342979	*PALM3*	0.328	47.962	7.70E−08
251	6249	*CLIP1*	−0.418	47.925	7.85E−08
252	358	*AQP1*	0.343	47.902	7.94E−08
253	54741	*LEPROT*	−0.394	47.891	7.99E−08
254	3164	*NR4A1*	0.362	47.789	8.41E−08
255	2155	*F7*	0.333	47.742	8.62E−08
256	5708	*PSMD2*	−0.385	47.740	8.62E−08
257	53826	*FXYD6*	0.351	47.717	8.73E−08
258	3033	*HADH*	0.323	47.709	8.76E−08
259	23092	*ARHGAP26*	0.325	47.694	8.83E−08
260	5727	*PTCH1*	0.337	47.694	8.83E−08
261	3383	*ICAM1*	−0.315	47.646	9.05E−08

262	59277	*NTN4*	−0.329	47.599	9.27E−08
263	9924	*PAN2*	0.312	47.599	9.27E−08
264	51202	*DDX47*	−0.384	47.587	9.32E−08
265	51129	*ANGPTL4*	−0.325	47.556	9.47E−08
266	9423	*NTN1*	−0.327	47.527	9.61E−08
267	127845	*GOLT1A*	0.329	47.501	9.75E−08
268	64116	*SLC39A8*	−0.356	47.486	9.82E−08
269	8908	*GYG2*	0.354	47.465	9.93E−08
270	114804	*RNF157*	0.326	47.463	9.94E−08
271	219654	*ZCCHC24*	0.329	47.421	1.02E−07
272	5525	*PPP2R5A*	0.307	47.396	1.03E−07
273	3549	*IHH*	0.366	47.392	1.03E−07
274	85377	*MICALL1*	−0.335	47.344	1.06E−07
275	26030	*PLEKHG3*	−0.387	47.313	1.07E−07
276	26262	*TSPAN17*	−0.344	47.311	1.07E−07
277	1861	*TOR1A*	−0.331	47.305	1.08E−07
278	64834	*ELOVL1*	−0.328	47.170	1.15E−07
279	10938	*EHD1*	−0.441	47.147	1.17E−07
280	6844	*VAMP2*	0.324	47.138	1.17E−07
281	308	*ANXA5*	−0.333	47.109	1.19E−07
282	1285	*COL4A3*	−0.315	47.100	1.20E−07
283	9766	*SUSD6*	−0.336	47.068	1.22E−07
284	159195	*USP54*	−0.330	47.004	1.26E−07
285	10197	*PSME3*	−0.385	46.954	1.29E−07
286	5793	*PTPRG*	−0.336	46.905	1.32E−07
287	57553	*MICAL3*	−0.370	46.839	1.37E−07
288	151636	*DTX3L*	−0.392	46.823	1.38E−07
289	5165	*PDK3*	0.320	46.797	1.40E−07
290	3423	*IDS*	−0.320	46.785	1.40E−07
291	29970	*SCHIP1*	−0.352	46.730	1.44E−07
292	51084	*CRYL1*	0.323	46.651	1.50E−07
293	23603	*CORO1C*	−0.396	46.626	1.52E−07
294	3916	*LAMP1*	−0.332	46.624	1.52E−07
295	9185	*REPS2*	−0.364	46.613	1.53E−07
296	57182	*ANKRD50*	−0.437	46.583	1.56E−07
297	5507	*PPP1R3C*	0.308	46.524	1.60E−07
298	25921	*ZDHHC5*	−0.351	46.476	1.64E−07
299	54442	*KCTD5*	−0.385	46.470	1.65E−07
300	11041	*B4GAT1*	0.322	46.442	1.67E−07
301	54948	*MRPL16*	0.336	46.389	1.72E−07
302	54915	*YTHDF1*	−0.312	46.385	1.72E−07
303	30851	*TAX1BP3*	−0.316	46.365	1.74E−07
304	64968	*MRPS6*	0.314	46.361	1.74E−07
305	8682	*PEA15*	−0.314	46.238	1.86E−07
306	341	*APOC1*	0.316	46.228	1.87E−07
307	11168	*PSIP1*	0.326	46.225	1.87E−07
308	5420	*PODXL*	0.337	46.122	1.97E−07
309	54798	*DCHS2*	−0.303	46.110	1.98E−07
310	93974	*ATP5IF1*	0.456	46.053	2.04E−07
311	6289	*SAA2*	0.311	45.914	2.19E−07
312	58510	*PRODH2*	0.319	45.890	2.22E−07
313	2946	*GSTM2*	0.321	45.847	2.27E−07
314	6288	*SAA1*	0.317	45.841	2.27E−07

315	2629	*GBA*	−0.325	45.821	2.30E−07
316	2941	*GSTA4*	0.310	45.815	2.30E−07
317	4673	*NAP1L1*	−0.354	45.745	2.39E−07
318	56997	*COQ8A*	0.317	45.698	2.45E−07
319	9900	*SV2A*	0.329	45.679	2.47E−07
320	23012	*STK38L*	−0.344	45.645	2.51E−07
321	55540	*IL17RB*	0.309	45.625	2.54E−07
322	1E+08	*LOC100419583*	−0.321	45.614	2.55E−07
323	324	*APC*	−0.403	45.566	2.62E−07
324	57048	*PLSCR3*	−0.487	45.478	2.74E−07
325	57493	*HEG1*	−0.374	45.408	2.83E−07
326	64432	*MRPS25*	0.318	45.372	2.89E−07
327	2330	*FMO5*	0.341	45.347	2.93E−07
328	23245	*ASTN2*	−0.314	45.345	2.93E−07
329	760	*CA2*	0.306	45.327	2.96E−07
330	10886	*NPFFR2*	−0.312	45.255	3.07E−07
331	10580	*SORBS1*	0.313	45.247	3.08E−07
332	57561	*ARRDC3*	−0.342	45.172	3.20E−07
333	6890	*TAP1*	−0.310	45.170	3.20E−07
334	307	*ANXA4*	−0.310	45.125	3.28E−07
335	24145	*PANX1*	−0.401	45.124	3.28E−07
336	2869	*GRK5*	0.320	45.122	3.28E−07
337	966	*CD59*	−0.347	45.096	3.32E−07
338	120892	*LRRK2*	0.314	45.066	3.38E−07
339	7132	*TNFRSF1A*	−0.344	45.048	3.41E−07
340	92126	*DSEL*	−0.361	44.959	3.57E−07
341	7264	*TSTA3*	−0.374	44.935	3.61E−07
342	10276	*NET1*	0.337	44.833	3.80E−07
343	59352	*LGR6*	0.315	44.826	3.82E−07
344	57630	*SH3RF1*	−0.335	44.778	3.91E−07
345	57480	*PLEKHG1*	−0.326	44.763	3.94E−07
346	6372	*CXCL6*	−0.294	44.749	3.97E−07
347	10841	*FTCD*	0.354	44.740	3.99E−07
348	6943	*TCF21*	0.319	44.660	4.16E−07
349	90293	*KLHL13*	0.302	44.627	4.23E−07
350	121512	*FGD4*	−0.395	44.601	4.28E−07
351	7067	*THRA*	0.536	44.585	4.32E−07
352	9308	*CD83*	0.314	44.560	4.37E−07
353	93166	*PRDM6*	0.329	44.511	4.48E−07
354	6867	*TACC1*	−0.376	44.469	4.58E−07
355	3673	*ITGA2*	−0.392	44.427	4.68E−07
356	9989	*PPP4R1*	−0.353	44.399	4.75E−07
357	126282	*TNFAIP8L1*	0.305	44.388	4.77E−07
358	861	*RUNX1*	−0.301	44.359	4.85E−07
359	6886	*TAL1*	−0.314	44.270	5.07E−07
360	93	*ACVR2B*	0.314	44.257	5.10E−07
361	50619	*DEF6*	0.338	44.217	5.21E−07
362	7165	*TPD52L2*	−0.332	44.188	5.29E−07
363	10627	*MYL12A*	−0.314	44.185	5.30E−07
364	9547	*CXCL14*	0.336	44.141	5.42E−07
365	3485	*IGFBP2*	−0.422	44.123	5.47E−07
366	10446	*LRRN2*	−0.322	44.091	5.56E−07
367	598	*BCL2L1*	−0.323	44.022	5.75E−07

368	2782	*GNB1*	−0.340	44.010	5.79E−07
369	10537	*UBD*	−0.304	44.007	5.80E−07
370	9462	*RASAL2*	−0.326	44.004	5.81E−07
371	84129	*ACAD11*	0.329	44.003	5.81E−07
372	7342	*UBP1*	−0.390	43.973	5.90E−07
373	3312	*HSPA8*	−0.517	43.938	6.01E−07
374	146754	*DNAH2*	0.300	43.880	6.19E−07
375	80223	*RAB11FIP1*	−0.339	43.853	6.27E−07
376	2810	*SFN*	−0.317	43.838	6.32E−07
377	51085	*MLXIPL*	0.320	43.806	6.43E−07
378	11223	*MST1L*	0.301	43.789	6.48E−07
379	51363	*CHST15*	−0.310	43.778	6.52E−07
380	11099	*PTPN21*	−0.319	43.708	6.76E−07
381	57706	*DENND1A*	0.330	43.704	6.77E−07
382	11075	*STMN2*	−0.309	43.692	6.81E−07
383	83786	*FRMD8*	−0.396	43.681	6.85E−07
384	54872	*PIGG*	−0.322	43.657	6.94E−07
385	163259	*DENND2C*	0.341	43.638	7.00E−07
386	1265	*CNN2*	−0.388	43.631	7.03E−07
387	9646	*CTR9*	−0.467	43.627	7.04E−07
388	1946	*EFNA5*	−0.311	43.584	7.20E−07
389	64753	*CCDC136*	0.330	43.546	7.34E−07
390	3177	*SLC29A2*	0.348	43.448	7.72E−07
391	6996	*TDG*	−0.375	43.373	8.02E−07
392	257144	*GCSAM*	−0.310	43.277	8.42E−07
393	187	*APLNR*	0.331	43.212	8.70E−07
394	55858	*TMEM165*	−0.306	43.211	8.71E−07
395	2274	*FHL2*	−0.327	43.206	8.73E−07
396	367	*AR*	−0.316	43.193	8.79E−07
397	89866	*SEC16B*	0.296	43.146	9.01E−07
398	54543	*TOMM7*	0.300	43.144	9.01E−07
399	6709	*SPTAN1*	−0.300	43.097	9.23E−07
400	1187	*CLCNKA*	0.319	43.092	9.26E−07
401	10049	*DNAJB6*	−0.306	43.078	9.32E−07
402	9754	*STARD8*	0.296	43.078	9.32E−07
403	51076	*CUTC*	0.301	43.045	9.48E−07
404	1473	*CST5*	−0.318	43.035	9.53E−07
405	79016	*DDA1*	−0.408	42.934	1.00E−06
406	8448	*DOC2A*	0.371	42.906	1.02E−06
407	5314	*PKHD1*	−0.322	42.877	1.03E−06
408	79649	*MAP7D3*	−0.320	42.855	1.05E−06
409	651746	*ANKRD33B*	−0.300	42.833	1.06E−06
410	401463	*LOC401463*	−0.298	42.812	1.07E−06
411	166824	*RASSF6*	−0.356	42.811	1.07E−06
412	5792	*PTPRF*	−0.300	42.795	1.08E−06
413	2861	*GPR37*	−0.309	42.769	1.09E−06
414	10050	*SLC17A4*	0.294	42.750	1.10E−06
415	388165	*UBE2Q2P1*	−0.318	42.729	1.11E−06
416	4741	*NEFM*	−0.312	42.690	1.14E−06
417	9936	*CD302*	0.302	42.675	1.15E−06
418	57169	*ZNFX1*	−0.322	42.664	1.15E−06
419	283248	*RCOR2*	0.363	42.647	1.16E−06
420	55884	*WSB2*	−0.321	42.638	1.17E−06

421	2131	*EXT1*	−0.309	42.503	1.25E−06
422	387521	*TMEM189*	−0.479	42.485	1.26E−06
423	10864	*SLC22A7*	0.300	42.453	1.28E−06
424	5139	*PDE3A*	−0.356	42.413	1.31E−06
425	388015	*RTL1*	0.299	42.332	1.37E−06
426	23677	*SH3BP4*	−0.323	42.312	1.38E−06
427	55343	*SLC35C1*	−0.314	42.305	1.38E−06
428	415	*ARSL*	0.308	42.219	1.45E−06
429	26227	*PHGDH*	0.310	42.194	1.47E−06
430	9056	*SLC7A7*	−0.324	42.128	1.52E−06
431	116835	*HSPA12B*	0.308	42.085	1.55E−06
432	283375	*SLC39A5*	0.319	42.082	1.55E−06
433	6615	*SNAI1*	0.312	42.026	1.60E−06
434	25819	*NOCT*	−0.302	41.958	1.65E−06
435	482	*ATP1B2*	0.312	41.949	1.66E−06
436	64902	*AGXT2*	0.305	41.928	1.68E−06
437	64780	*MICAL1*	−0.350	41.903	1.70E−06
438	4013	*VWA5A*	−0.354	41.877	1.72E−06
439	3216	*HOXB6*	0.326	41.752	1.84E−06
440	132158	*GLYCTK*	0.319	41.683	1.90E−06
441	3486	*IGFBP3*	0.331	41.657	1.93E−06
442	10402	*ST3GAL6*	0.292	41.629	1.96E−06
443	153129	*SLC38A9*	−0.344	41.610	1.97E−06
444	1962	*EHHADH*	0.358	41.602	1.98E−06
445	91283	*MSANTD3*	−0.329	41.554	2.03E−06
446	284422	*SMIM24*	0.298	41.552	2.03E−06
447	6553	*SLC9A5*	0.369	41.522	2.07E−06
448	8994	*LIMD1*	−0.301	41.502	2.09E−06
449	5922	*RASA2*	−0.305	41.497	2.09E−06
450	55107	*ANO1*	0.296	41.483	2.11E−06
451	5018	*OXA1L*	0.365	41.467	2.12E−06
452	199731	*CADM4*	−0.352	41.450	2.14E−06
453	10093	*ARPC4*	−0.355	41.417	2.18E−06
454	50862	*RNF141*	−0.409	41.409	2.19E−06
455	54097	*FAM3B*	0.296	41.406	2.19E−06
456	221393	*ADGRF4*	−0.311	41.380	2.22E−06
457	1238	*ACKR2*	−0.311	41.348	2.26E−06
458	3692	*EIF6*	−0.329	41.240	2.39E−06
459	23176	*SEPTIN8*	−0.307	41.206	2.43E−06
460	23240	*TMEM131L*	−0.345	41.148	2.50E−06
461	9963	*SLC23A1*	0.299	41.143	2.51E−06
462	323	*APBB2*	−0.318	41.121	2.54E−06
463	25957	*PNISR*	0.317	41.121	2.54E−06
464	1000	*CDH2*	−0.346	41.101	2.56E−06
465	799	*CALCR*	−0.295	41.094	2.57E−06
466	4285	*MIPEP*	−0.353	41.072	2.60E−06
467	81563	*C1orf21*	0.303	41.054	2.62E−06
468	9180	*OSMR*	−0.372	41.039	2.65E−06
469	2752	*GLUL*	0.302	41.030	2.66E−06
470	91860	*CALML4*	0.335	40.917	2.82E−06
471	3373	*HYAL1*	0.293	40.894	2.85E−06
472	10962	*MLLT11*	−0.303	40.872	2.88E−06
473	9232	*PTTG1*	0.307	40.862	2.90E−06

474	57447	*NDRG2*	0.314	40.835	2.94E−06
475	118788	*PIK3AP1*	−0.325	40.833	2.94E−06
476	80381	*CD276*	−0.324	40.829	2.94E−06
477	23344	*ESYT1*	−0.315	40.811	2.97E−06
478	55761	*TTC17*	0.336	40.757	3.06E−06
479	9044	*BTAF1*	0.335	40.737	3.09E−06
480	27346	*TMEM97*	0.303	40.718	3.12E−06
481	11135	*CDC42EP1*	−0.362	40.716	3.12E−06
482	343637	*RSPO4*	0.315	40.673	3.19E−06
483	2919	*CXCL1*	−0.287	40.657	3.22E−06
484	7534	*YWHAZ*	−0.323	40.656	3.22E−06
485	9421	*HAND1*	0.327	40.635	3.25E−06
486	823	*CAPN1*	−0.333	40.600	3.31E−06
487	5948	*RBP2*	0.295	40.574	3.36E−06
488	64747	*MFSD1*	−0.381	40.561	3.38E−06
489	80176	*SPSB1*	−0.314	40.498	3.49E−06
490	8153	*RND2*	0.301	40.494	3.50E−06
491	256435	*ST6GALNAC3*	0.309	40.480	3.52E−06
492	4790	*NFKB1*	−0.363	40.388	3.69E−06
493	128338	*DRAM2*	0.292	40.360	3.74E−06
494	84898	*PLXDC2*	−0.314	40.334	3.79E−06
495	55332	*DRAM1*	0.297	40.231	4.00E−06
496	3750	*KCND1*	0.397	40.221	4.02E−06
497	404093	*CUEDC1*	−0.355	40.208	4.05E−06
498	4678	*NASP*	0.295	40.196	4.07E−06
499	84148	*KAT8*	0.391	40.180	4.11E−06
500	7048	*TGFBR2*	−0.384	40.179	4.11E−06
501	1072	*CFL1*	−0.362	40.177	4.11E−06
502	30011	*SH3KBP1*	−0.286	40.163	4.14E−06
503	25996	*REXO2*	−0.299	40.094	4.29E−06
504	420	*ART4*	0.420	40.062	4.36E−06
505	5819	*NECTIN2*	−0.364	40.059	4.37E−06
506	2316	*FLNA*	−0.301	40.037	4.42E−06
507	80271	*ITPKC*	−0.303	40.031	4.43E−06
508	65084	*TMEM135*	0.320	39.944	4.63E−06
509	57633	*LRRN1*	−0.322	39.918	4.69E−06
510	51114	*ZDHHC9*	−0.298	39.908	4.72E−06
511	1802	*DPH2*	−0.299	39.834	4.90E−06
512	5066	*PAM*	−0.355	39.831	4.91E−06
513	116984	*ARAP2*	−0.371	39.778	5.04E−06
514	5464	*PPA1*	−0.330	39.766	5.08E−06
515	25788	*RAD54B*	0.308	39.759	5.09E−06
516	3840	*KPNA4*	−0.397	39.695	5.26E−06
517	9369	*NRXN3*	−0.306	39.693	5.27E−06
518	5739	*PTGIR*	0.322	39.688	5.28E−06
519	284111	*SLC13A5*	0.283	39.670	5.33E−06
520	5330	*PLCB2*	0.310	39.595	5.54E−06
521	387640	*SKIDA1*	0.290	39.567	5.62E−06
522	25837	*RAB26*	0.335	39.566	5.62E−06
523	7732	*RNF112*	0.298	39.448	5.97E−06
524	3728	*JUP*	−0.341	39.395	6.14E−06
525	32	*ACACB*	0.287	39.384	6.17E−06
526	80111	*C3orf36*	−0.283	39.346	6.29E−06

527	51573	*GDE1*	−0.317	39.323	6.37E−06
528	7137	*TNNI3*	0.300	39.270	6.54E−06
529	11112	*HIBADH*	0.285	39.266	6.55E−06
530	130589	*GALM*	0.297	39.176	6.86E−06
531	4864	*NPC1*	−0.333	39.168	6.89E−06
532	133686	*NADK2*	0.308	39.158	6.93E−06
533	9557	*CHD1L*	0.307	39.123	7.05E−06
534	85364	*ZCCHC3*	0.340	39.123	7.05E−06
535	170690	*ADAMTS16*	−0.335	39.120	7.06E−06
536	678	*ZFP36L2*	0.314	39.109	7.10E−06
537	1.01E+08	*LINC00963*	−0.297	39.027	7.41E−06
538	9097	*USP14*	−0.359	39.009	7.48E−06
539	84978	*FRMD5*	−0.294	39.001	7.51E−06
540	114800	*CCDC85A*	−0.314	38.995	7.53E−06
541	79188	*TMEM43*	−0.290	38.985	7.57E−06
542	6324	*SCN1B*	−0.324	38.971	7.62E−06
543	81558	*FAM117A*	0.320	38.959	7.67E−06
544	395	*ARHGAP6*	0.285	38.942	7.74E−06
545	10863	*ADAM28*	−0.298	38.930	7.79E−06
546	25852	*ARMC8*	−0.375	38.926	7.80E−06
547	334	*APLP2*	−0.317	38.875	8.01E−06
548	55937	*APOM*	0.289	38.835	8.18E−06
549	55841	*WWC3*	−0.291	38.809	8.28E−06
550	51203	*NUSAP1*	0.292	38.801	8.32E−06
551	5216	*PFN1*	−0.423	38.800	8.32E−06
552	3837	*KPNB1*	−0.385	38.778	8.42E−06
553	5095	*PCCA*	0.321	38.773	8.44E−06
554	200010	*SLC5A9*	0.304	38.730	8.63E−06
555	3688	*ITGB1*	−0.402	38.624	9.11E−06
556	158326	*FREM1*	0.282	38.610	9.18E−06
557	494470	*RNF165*	0.364	38.551	9.45E−06
558	7468	*NSD2*	0.314	38.546	9.48E−06
559	5118	*PCOLCE*	0.312	38.545	9.48E−06
560	858	*CAV2*	−0.316	38.497	9.72E−06
561	55825	*PECR*	0.301	38.495	9.73E−06
562	79858	*NEK11*	−0.359	38.491	9.75E−06
563	55893	*ZNF395*	0.288	38.468	9.86E−06
564	55276	*PGM2*	−0.424	38.458	9.92E−06
565	55765	*INAVA*	−0.306	38.416	1.01E−05
566	229	*ALDOB*	0.288	38.407	1.02E−05
567	81788	*NUAK2*	−0.358	38.374	1.04E−05
568	9482	*STX8*	0.289	38.356	1.04E−05
569	364	*AQP7*	0.302	38.326	1.06E−05
570	8536	*CAMK1*	0.352	38.321	1.06E−05
571	8643	*PTCH2*	0.344	38.295	1.08E−05
572	11072	*DUSP14*	−0.292	38.294	1.08E−05
573	387758	*FIBIN*	0.297	38.287	1.08E−05
574	2139	*EYA2*	−0.286	38.286	1.08E−05
575	10584	*COLEC10*	0.300	38.261	1.10E−05
576	79170	*PRR15L*	0.281	38.260	1.10E−05
577	90861	*JPT2*	−0.335	38.217	1.12E−05
578	1129	*CHRM2*	0.272	38.215	1.12E−05
579	4837	*NNMT*	−0.290	38.167	1.15E−05

580	197257	*LDHD*	0.300	38.104	1.19E−05
581	5358	*PLS3*	−0.379	38.074	1.21E−05
582	7247	*TSN*	−0.353	38.073	1.21E−05
583	7138	*TNNT1*	0.302	38.025	1.24E−05
584	25844	*YIPF3*	−0.337	38.012	1.25E−05
585	51474	*LIMA1*	−0.372	38.009	1.25E−05
586	57464	*STRIP2*	−0.292	37.980	1.27E−05
587	9886	*RHOBTB1*	−0.370	37.977	1.27E−05
588	23705	*CADM1*	−0.307	37.973	1.27E−05
589	22808	*MRAS*	−0.279	37.956	1.28E−05
590	10082	*GPC6*	−0.383	37.892	1.33E−05
591	4547	*MTTP*	0.286	37.866	1.34E−05
592	3158	*HMGCS2*	0.287	37.861	1.35E−05
593	6581	*SLC22A3*	0.311	37.847	1.36E−05
594	115290	*FBXO17*	0.312	37.812	1.38E−05
595	79574	*EPS8L3*	0.298	37.811	1.38E−05
596	55211	*DPPA4*	0.286	37.773	1.41E−05
597	1.01E+08	*MIR210HG*	0.311	37.743	1.43E−05
598	388969	*C2orf68*	0.329	37.735	1.44E−05
599	51155	*JPT1*	−0.312	37.733	1.44E−05
600	112724	*RDH13*	−0.288	37.705	1.46E−05
601	148932	*MOB3C*	0.281	37.693	1.47E−05
602	79987	*SVEP1*	0.300	37.680	1.48E−05
603	51087	*YBX2*	0.335	37.671	1.48E−05
604	51090	*PLLP*	0.306	37.645	1.50E−05
605	54853	*WDR55*	−0.346	37.515	1.61E−05
606	10278	*EFS*	0.328	37.477	1.64E−05
607	8878	*SQSTM1*	−0.353	37.462	1.65E−05
608	8829	*NRP1*	−0.314	37.458	1.66E−05
609	203	*AK1*	0.313	37.455	1.66E−05
610	79849	*PDZD3*	0.290	37.411	1.70E−05
611	1644	*DDC*	0.304	37.407	1.70E−05
612	23225	*NUP210*	0.295	37.387	1.72E−05
613	729582	*DIRC3*	0.289	37.330	1.77E−05
614	254122	*SNX32*	0.297	37.326	1.77E−05
615	81603	*TRIM8*	−0.401	37.323	1.77E−05
616	2669	*GEM*	0.307	37.305	1.79E−05
617	80221	*ACSF2*	0.284	37.257	1.84E−05
618	217	*ALDH2*	0.316	37.218	1.87E−05
619	79960	*JADE1*	−0.290	37.193	1.90E−05
620	4507	*MTAP*	−0.323	37.142	1.95E−05
621	55286	*C4orf19*	−0.312	37.142	1.95E−05
622	113419	*TEX261*	−0.290	37.139	1.95E−05
623	8322	*FZD4*	0.279	37.124	1.97E−05
624	23492	*CBX7*	0.306	37.077	2.01E−05
625	7148	*TNXB*	0.293	37.070	2.02E−05
626	84251	*SGIP1*	0.285	37.047	2.04E−05
627	634	*CEACAM1*	−0.293	36.997	2.10E−05
628	54899	*PXK*	0.325	36.994	2.10E−05
629	3482	*IGF2R*	−0.425	36.973	2.12E−05
630	22800	*RRAS2*	−0.277	36.957	2.14E−05
631	57136	*APMAP*	−0.291	36.924	2.18E−05
632	7049	*TGFBR3*	0.292	36.913	2.19E−05

633	134429	*STARD4*	0.333	36.890	2.22E−05
634	27063	*ANKRD1*	−0.282	36.875	2.23E−05
635	6891	*TAP2*	−0.285	36.874	2.23E−05
636	167410	*LIX1*	0.288	36.867	2.24E−05
637	8869	*ST3GAL5*	0.312	36.843	2.27E−05
638	7094	*TLN1*	−0.278	36.814	2.30E−05
639	91614	*DEPDC7*	0.285	36.810	2.31E−05
640	2239	*GPC4*	−0.296	36.749	2.38E−05
641	6936	*GCFC2*	0.276	36.715	2.42E−05
642	10456	*HAX1*	0.282	36.685	2.46E−05
643	85021	*REPS1*	0.299	36.683	2.46E−05
644	27309	*ZNF330*	0.306	36.668	2.48E−05
645	11252	*PACSIN2*	−0.321	36.643	2.51E−05
646	26010	*SPATS2L*	−0.294	36.635	2.52E−05
647	9498	*SLC4A8*	0.288	36.606	2.56E−05
648	84960	*CCDC183*	0.285	36.561	2.62E−05
649	5225	*PGC*	0.279	36.520	2.68E−05
650	5878	*RAB5C*	−0.279	36.487	2.72E−05
651	6609	*SMPD1*	−0.288	36.478	2.74E−05
652	57820	*CCNB1IP1*	0.288	36.469	2.75E−05
653	57536	*KIAA1328*	0.275	36.435	2.80E−05
654	55102	*ATG2B*	0.327	36.391	2.86E−05
655	94234	*FOXQ1*	0.302	36.379	2.88E−05
656	375484	*SIMC1*	0.283	36.331	2.95E−05
657	9104	*RGN*	0.274	36.223	3.12E−05
658	54346	*UNC93A*	0.272	36.165	3.21E−05
659	1050	*CEBPA*	0.328	36.160	3.22E−05
660	1293	*COL6A3*	0.292	36.126	3.28E−05
661	26509	*MYOF*	−0.326	36.063	3.39E−05
662	7010	*TEK*	0.292	36.016	3.47E−05
663	256394	*SERPINA11*	0.279	35.998	3.50E−05
664	58	*ACTA1*	−0.293	35.991	3.51E−05
665	6718	*AKR1D1*	0.277	35.976	3.54E−05
666	286343	*LURAP1L*	−0.282	35.937	3.61E−05
667	79154	*DHRS11*	0.316	35.926	3.63E−05
668	283310	*OTOGL*	−0.281	35.915	3.65E−05
669	84265	*POLR3GL*	0.276	35.889	3.70E−05
670	59342	*SCPEP1*	0.277	35.863	3.75E−05
671	94015	*TTYH2*	0.286	35.844	3.79E−05
672	4772	*NFATC1*	0.310	35.828	3.82E−05
673	6328	*SCN3A*	−0.307	35.816	3.84E−05
674	3218	*HOXB8*	0.307	35.807	3.86E−05
675	728377	*ARHGEF34P*	−0.303	35.801	3.87E−05
676	51232	*CRIM1*	−0.332	35.780	3.92E−05
677	8602	*NOP14*	−0.352	35.780	3.92E−05
678	1649	*DDIT3*	0.304	35.772	3.93E−05
679	55974	*SLC50A1*	0.281	35.736	4.00E−05
680	3213	*HOXB3*	0.322	35.724	4.03E−05
681	56033	*BARX1*	−0.306	35.723	4.03E−05
682	79567	*RIPOR1*	−0.324	35.712	4.05E−05
683	8309	*ACOX2*	0.283	35.709	4.06E−05
684	51776	*MAP3K20*	−0.370	35.688	4.11E−05
685	84962	*AJUBA*	−0.284	35.669	4.15E−05

686	26115	*TANC2*	−0.314	35.634	4.22E−05
687	7260	*EIPR1*	−0.289	35.626	4.24E−05
688	55422	*ZNF331*	0.270	35.622	4.25E−05
689	79153	*GDPD3*	0.309	35.577	4.35E−05
690	2326	*FMO1*	0.281	35.548	4.41E−05
691	862	*RUNX1T1*	0.290	35.511	4.50E−05
692	340348	*TSPAN33*	0.309	35.506	4.51E−05
693	115557	*ARHGEF25*	0.324	35.503	4.51E−05
694	85463	*ZC3H12C*	−0.329	35.466	4.60E−05
695	9079	*LDB2*	0.289	35.453	4.63E−05
696	401399	*PRRT4*	0.318	35.441	4.66E−05
697	4248	*MGAT3*	0.307	35.441	4.66E−05
698	56300	*IL36G*	0.296	35.434	4.68E−05
699	84246	*MED10*	−0.298	35.421	4.71E−05
700	4817	*NIT1*	0.321	35.393	4.78E−05
701	11209	*MST1P2*	0.341	35.382	4.80E−05
702	57715	*SEMA4G*	0.301	35.355	4.87E−05
703	7171	*TPM4*	−0.287	35.351	4.88E−05
704	8218	*CLTCL1*	−0.274	35.339	4.91E−05
705	1635	*DCTD*	−0.279	35.305	5.00E−05
706	10488	*CREB3*	−0.293	35.293	5.03E−05
707	10247	*RIDA*	0.274	35.279	5.06E−05
708	2260	*FGFR1*	0.343	35.278	5.07E−05
709	11057	*ABHD2*	−0.416	35.238	5.17E−05
710	7042	*TGFB2*	−0.303	35.234	5.18E−05
711	6430	*SRSF5*	0.331	35.227	5.20E−05
712	6251	*RSU1*	−0.270	35.206	5.26E−05
713	29940	*DSE*	−0.273	35.190	5.30E−05
714	5664	*PSEN2*	−0.272	35.184	5.32E−05
715	6548	*SLC9A1*	−0.296	35.179	5.33E−05
716	9208	*LRRFIP1*	−0.317	35.166	5.37E−05
717	5158	*PDE6B*	0.284	35.144	5.43E−05
718	51430	*SUCO*	−0.344	35.128	5.47E−05
719	389136	*VGLL3*	−0.314	35.120	5.50E−05
720	8334	*H2AC6*	−0.268	35.103	5.54E−05
721	25890	*ABI3BP*	0.277	35.053	5.69E−05
722	55200	*PLEKHG6*	0.277	35.046	5.71E−05
723	64411	*ARAP3*	0.306	35.045	5.71E−05
724	401613	*SERTM2*	0.319	35.037	5.73E−05
725	11161	*ERG28*	0.315	35.036	5.74E−05
726	133584	*EGFLAM*	0.282	35.023	5.78E−05
727	84695	*LOXL3*	0.300	35.007	5.82E−05
728	1958	*EGR1*	0.275	34.996	5.85E−05
729	1357	*CPA1*	0.281	34.975	5.92E−05
730	2295	*FOXF2*	0.295	34.961	5.96E−05
731	84830	*ADTRP*	0.275	34.906	6.13E−05
732	64062	*RBM26*	0.422	34.904	6.14E−05
733	4921	*DDR2*	0.303	34.877	6.22E−05
734	171425	*CLYBL*	0.269	34.869	6.25E−05
735	4060	*LUM*	0.293	34.845	6.33E−05
736	81037	*CLPTM1L*	−0.273	34.828	6.39E−05
737	146850	*PIK3R6*	0.277	34.755	6.63E−05
738	23326	*USP22*	−0.277	34.724	6.73E−05

739	84284	*NTPCR*	0.304	34.697	6.83E−05
740	780	*DDR1*	−0.305	34.676	6.90E−05
741	51809	*GALNT7*	−0.314	34.671	6.92E−05
742	2159	*F10*	0.302	34.651	6.99E−05
743	1428	*CRYM*	0.271	34.629	7.07E−05
744	114034	*TOE1*	0.274	34.623	7.09E−05
745	643201	*LOC643201*	0.286	34.593	7.20E−05
746	4205	*MEF2A*	0.285	34.565	7.31E−05
747	171024	*SYNPO2*	0.283	34.509	7.52E−05
748	3685	*ITGAV*	−0.362	34.460	7.71E−05
749	284439	*SLC25A42*	0.313	34.456	7.73E−05
750	84649	*DGAT2*	0.288	34.449	7.75E−05
751	247	*ALOX15B*	0.303	34.402	7.94E−05
752	7984	*ARHGEF5*	−0.317	34.376	8.05E−05
753	4634	*MYL3*	0.304	34.368	8.09E−05
754	9842	*PLEKHM1*	−0.279	34.342	8.19E−05
755	6492	*SIM1*	−0.285	34.327	8.26E−05
756	23229	*ARHGEF9*	0.272	34.317	8.30E−05
757	5501	*PPP1CC*	0.330	34.288	8.43E−05
758	283659	*PRTG*	0.305	34.211	8.76E−05
759	10319	*LAMC3*	0.298	34.194	8.84E−05
760	122953	*JDP2*	0.346	34.177	8.92E−05
761	27075	*TSPAN13*	−0.288	34.167	8.96E−05
762	5796	*PTPRK*	−0.435	34.137	9.11E−05
763	5017	*OVOL1*	0.274	34.113	9.22E−05
764	26508	*HEYL*	0.285	34.095	9.30E−05
765	59344	*ALOXE3*	−0.272	34.053	9.51E−05
766	56950	*SMYD2*	−0.275	34.043	9.55E−05
767	255928	*SYT14*	−0.328	34.034	9.60E−05
768	56902	*PNO1*	−0.334	34.015	9.70E−05
769	25939	*SAMHD1*	0.275	33.998	9.78E−05
770	9464	*HAND2*	0.291	33.989	9.82E−05
771	253769	*WDR27*	0.311	33.970	9.92E−05
772	64168	*NECAB1*	0.270	33.927	1.01E−04
773	284325	*C19orf54*	0.381	33.892	1.03E−04
774	26094	*DCAF4*	−0.279	33.861	1.05E−04
775	2984	*GUCY2C*	0.276	33.859	1.05E−04
776	39	*ACAT2*	0.316	33.857	1.05E−04
777	51382	*ATP6V1D*	−0.307	33.835	1.06E−04
778	6988	*TCTA*	−0.283	33.790	1.09E−04
779	8609	*KLF7*	−0.305	33.742	1.12E−04
780	27072	*VPS41*	−0.333	33.735	1.12E−04
781	93436	*ARMC6*	−0.315	33.730	1.12E−04
782	516	*ATP5MC1*	0.319	33.711	1.13E−04
783	4664	*NAB1*	0.272	33.698	1.14E−04
784	666	*BOK*	−0.361	33.632	1.18E−04
785	58489	*ABHD17C*	−0.287	33.614	1.19E−04
786	80854	*SETD7*	−0.379	33.558	1.23E−04
787	9332	*CD163*	0.264	33.553	1.23E−04
788	2706	*GJB2*	0.285	33.553	1.23E−04
789	4239	*MFAP4*	0.288	33.538	1.24E−04
790	80228	*ORAI2*	−0.275	33.532	1.24E−04
791	401251	*SAPCD1*	0.309	33.524	1.25E−04

792	203328	*SUSD3*	0.287	33.522	1.25E−04
793	347735	*SERINC2*	−0.346	33.507	1.26E−04
794	8644	*AKR1C3*	0.264	33.491	1.27E−04
795	6124	*RPL4*	0.267	33.482	1.27E−04
796	23324	*MAN2B2*	−0.276	33.472	1.28E−04
797	51303	*FKBP11*	0.349	33.459	1.29E−04
798	10563	*CXCL13*	0.266	33.449	1.30E−04
799	81928	*CABLES2*	0.284	33.437	1.30E−04
800	5062	*PAK2*	−0.360	33.424	1.31E−04
801	23386	*NUDCD3*	−0.272	33.415	1.32E−04
802	55690	*PACS1*	−0.347	33.409	1.32E−04
803	8936	*WASF1*	0.285	33.407	1.32E−04
804	60676	*PAPPA2*	0.267	33.398	1.33E−04
805	8991	*SELENBP1*	0.291	33.382	1.34E−04
806	11137	*PWP1*	−0.342	33.365	1.35E−04
807	91010	*FMNL3*	0.290	33.359	1.36E−04
808	635	*BHMT*	0.268	33.350	1.36E−04
809	57647	*DHX37*	−0.283	33.329	1.38E−04
810	55179	*FAIM*	−0.292	33.324	1.38E−04
811	160851	*DGKH*	−0.300	33.289	1.41E−04
812	54888	*NSUN2*	−0.332	33.264	1.43E−04
813	1.01E+08	*LINC01356*	0.263	33.260	1.43E−04
814	23098	*SARM1*	−0.273	33.219	1.46E−04
815	112476	*PRRT2*	0.282	33.212	1.46E−04
816	256356	*GK5*	0.308	33.207	1.47E−04
817	103910	*MYL12B*	−0.277	33.197	1.48E−04
818	284656	*EPHA10*	−0.282	33.195	1.48E−04
819	6405	*SEMA3F*	0.293	33.179	1.49E−04
820	10892	*MALT1*	−0.268	33.159	1.51E−04
821	2203	*FBP1*	0.279	33.135	1.52E−04
822	9980	*DOP1B*	−0.284	33.117	1.54E−04
823	54521	*WDR44*	−0.401	33.113	1.54E−04
824	5660	*PSAP*	−0.320	33.112	1.54E−04
825	342	*APOC1P1*	0.274	33.109	1.54E−04
826	6676	*SPAG4*	0.278	33.062	1.58E−04
827	112611	*RWDD2A*	0.281	33.057	1.59E−04
828	28986	*MAGEH1*	0.266	33.042	1.60E−04
829	23589	*CARHSP1*	−0.295	33.017	1.62E−04
830	23582	*CCNDBP1*	−0.308	32.992	1.64E−04
831	8303	*SNN*	−0.271	32.991	1.64E−04
832	571	*BACH1*	−0.349	32.974	1.66E−04
833	9118	*INA*	−0.291	32.943	1.68E−04
834	84263	*HSDL2*	0.315	32.942	1.68E−04
835	2110	*ETFDH*	0.325	32.927	1.70E−04
836	344595	*DUBR*	−0.302	32.906	1.71E−04
837	27239	*GPR162*	0.303	32.863	1.75E−04
838	151306	*GPBAR1*	0.317	32.838	1.78E−04
839	29919	*RMC1*	−0.274	32.822	1.79E−04
840	7306	*TYRP1*	0.282	32.810	1.80E−04
841	29978	*UBQLN2*	−0.284	32.806	1.80E−04
842	4345	*CD200*	−0.270	32.794	1.82E−04
843	60626	*RIC8A*	−0.280	32.766	1.84E−04
844	56243	*KIAA1217*	0.277	32.737	1.87E−04

845	64922	*LRRC19*	0.262	32.711	1.90E−04
846	2304	*FOXE1*	−0.266	32.686	1.92E−04
847	338094	*FAM151A*	0.270	32.645	1.96E−04
848	3801	*KIFC3*	−0.339	32.632	1.97E−04
849	9110	*MTMR4*	0.275	32.610	2.00E−04
850	57333	*RCN3*	0.327	32.602	2.00E−04
851	2224	*FDPS*	0.291	32.587	2.02E−04
852	1612	*DAPK1*	−0.265	32.567	2.04E−04
853	84002	*B3GNT5*	−0.336	32.561	2.05E−04
854	387882	*C12orf75*	−0.285	32.546	2.06E−04
855	84274	*COQ5*	0.270	32.546	2.06E−04
856	4224	*MEP1A*	0.273	32.537	2.07E−04
857	409	*ARRB2*	0.282	32.525	2.09E−04
858	29954	*POMT2*	−0.289	32.508	2.10E−04
859	6817	*SULT1A1*	0.283	32.494	2.12E−04
860	3417	*IDH1*	0.301	32.491	2.12E−04
861	2901	*GRIK5*	0.302	32.479	2.14E−04
862	2735	*GLI1*	0.291	32.459	2.16E−04
863	7415	*VCP*	−0.275	32.446	2.17E−04
864	9094	*UNC119*	0.325	32.424	2.20E−04
865	977	*CD151*	−0.347	32.406	2.22E−04
866	7529	*YWHAB*	−0.386	32.405	2.22E−04
867	1982	*EIF4G2*	−0.353	32.401	2.22E−04
868	83666	*PARP9*	−0.337	32.399	2.22E−04
869	3702	*ITK*	−0.274	32.394	2.23E−04
870	9580	*SOX13*	0.305	32.360	2.27E−04
871	80146	*UXS1*	−0.299	32.358	2.27E−04
872	5550	*PREP*	−0.284	32.350	2.28E−04
873	65083	*NOL6*	−0.266	32.349	2.28E−04
874	7512	*XPNPEP2*	0.273	32.329	2.31E−04
875	23236	*PLCB1*	−0.321	32.294	2.35E−04
876	80736	*SLC44A4*	0.271	32.287	2.36E−04
877	9702	*CEP57*	0.309	32.275	2.37E−04
878	6993	*DYNLT1*	−0.268	32.202	2.46E−04
879	80332	*ADAM33*	0.295	32.197	2.47E−04
880	26220	*DGCR5*	−0.265	32.191	2.48E−04
881	744	*MPPED2*	0.285	32.157	2.52E−04
882	8565	*YARS1*	0.268	32.155	2.52E−04
883	129049	*SGSM1*	0.276	32.136	2.55E−04
884	28970	*C11orf54*	0.289	32.134	2.55E−04
885	399671	*HEATR4*	0.258	32.128	2.56E−04
886	57687	*VAT1L*	0.286	32.127	2.56E−04
887	9975	*NR1D2*	0.306	32.125	2.56E−04
888	63898	*SH2D4A*	−0.301	32.119	2.57E−04
889	80820	*EEPD1*	−0.267	32.100	2.60E−04
890	55273	*TMEM100*	0.274	32.063	2.65E−04
891	54780	*NSMCE4A*	0.271	32.051	2.66E−04
892	6860	*SYT4*	0.268	32.050	2.66E−04
893	115361	*GBP4*	0.270	32.020	2.70E−04
894	158405	*KIAA1958*	0.265	31.972	2.77E−04
895	4487	*MSX1*	0.305	31.961	2.79E−04
896	7347	*UCHL3*	−0.288	31.952	2.80E−04
897	1.05E+08	*LNCOG*	−0.285	31.947	2.81E−04

898	201161	*CENPV*	0.321	31.917	2.85E−04
899	51301	*GCNT4*	−0.277	31.903	2.87E−04
900	2294	*FOXF1*	0.292	31.888	2.89E−04
901	56937	*PMEPA1*	−0.304	31.888	2.90E−04
902	57639	*CCDC146*	0.264	31.877	2.91E−04
903	56674	*TMEM9B*	−0.288	31.875	2.91E−04
904	1639	*DCTN1*	−0.273	31.851	2.95E−04
905	6894	*TARBP1*	0.267	31.839	2.97E−04
906	285313	*IGSF10*	0.272	31.816	3.00E−04
907	2982	*GUCY1A1*	0.277	31.815	3.01E−04
908	486	*FXYD2*	0.272	31.798	3.03E−04
909	1E+08	*RRN3P3*	−0.258	31.789	3.05E−04
910	23189	*KANK1*	0.292	31.784	3.05E−04
911	5549	*PRELP*	0.266	31.784	3.05E−04
912	25874	*MPC2*	0.288	31.781	3.06E−04
913	3795	*KHK*	0.264	31.774	3.07E−04
914	57555	*NLGN2*	0.403	31.719	3.16E−04
915	51744	*CD244*	0.257	31.714	3.17E−04
916	11221	*DUSP10*	−0.274	31.686	3.21E−04
917	4046	*LSP1*	0.276	31.668	3.24E−04
918	93058	*COQ10A*	0.291	31.651	3.27E−04
919	10121	*ACTR1A*	−0.256	31.651	3.27E−04
920	51582	*AZIN1*	−0.345	31.641	3.29E−04
921	1628	*DBP*	0.362	31.634	3.30E−04
922	84870	*RSPO3*	0.286	31.623	3.32E−04
923	728464	*METTL24*	0.329	31.615	3.33E−04
924	81617	*CAB39L*	0.277	31.600	3.36E−04
925	169044	*COL22A1*	−0.259	31.587	3.38E−04
926	1191	*CLU*	0.267	31.569	3.41E−04
927	5160	*PDHA1*	0.260	31.550	3.44E−04
928	1027	*CDKN1B*	0.266	31.538	3.47E−04
929	3268	*AGFG2*	0.277	31.529	3.48E−04
930	1E+08	*FAR2P2*	0.263	31.525	3.49E−04
931	8091	*HMGA2*	0.281	31.518	3.50E−04
932	220002	*CYB561A3*	−0.286	31.510	3.52E−04
933	400221	*FLJ22447*	−0.261	31.480	3.57E−04
934	8935	*SKAP2*	−0.340	31.474	3.58E−04
935	56181	*MTFR1L*	0.280	31.438	3.65E−04
936	1E+08	*C8orf88*	0.267	31.424	3.68E−04
937	166929	*SGMS2*	−0.344	31.419	3.69E−04
938	726	*CAPN5*	0.263	31.413	3.70E−04
939	56271	*BEX4*	0.303	31.412	3.70E−04
940	55240	*STEAP3*	−0.261	31.400	3.72E−04
941	5608	*MAP2K6*	0.304	31.397	3.73E−04
942	664	*BNIP3*	0.276	31.395	3.73E−04
943	10420	*TESK2*	0.266	31.377	3.77E−04
944	25875	*LETMD1*	0.384	31.375	3.77E−04
945	4258	*MGST2*	0.266	31.364	3.79E−04
946	27173	*SLC39A1*	−0.256	31.321	3.88E−04
947	3242	*HPD*	0.296	31.304	3.91E−04
948	5451	*POU2F1*	0.275	31.288	3.94E−04
949	9586	*CREB5*	−0.270	31.256	4.01E−04
950	126661	*CCDC163*	0.264	31.251	4.02E−04

951	23596	*OPN3*	−0.285	31.241	4.04E−04
952	79183	*TTPAL*	−0.314	31.231	4.06E−04
953	10614	*HEXIM1*	0.305	31.224	4.08E−04
954	23484	*LEPROTL1*	−0.262	31.196	4.13E−04
955	201562	*HACD2*	−0.320	31.187	4.15E−04
956	220323	*OAF*	0.323	31.177	4.17E−04
957	4439	*MSH5*	0.301	31.175	4.18E−04
958	9780	*PIEZO1*	−0.307	31.149	4.24E−04
959	3646	*EIF3E*	0.334	31.088	4.37E−04
960	10682	*EBP*	0.272	31.057	4.44E−04
961	7846	*TUBA1A*	−0.279	31.053	4.45E−04
962	57486	*NLN*	−0.313	31.048	4.46E−04
963	5320	*PLA2G2A*	0.276	31.044	4.47E−04
964	2775	*GNAO1*	−0.261	31.042	4.48E−04
965	284361	*EMC10*	−0.401	31.020	4.53E−04
966	89876	*MAATS1*	0.283	31.019	4.53E−04
967	6565	*SLC15A2*	−0.283	30.930	4.74E−04
968	30850	*CDR2L*	−0.338	30.927	4.75E−04
969	7920	*ABHD16A*	−0.332	30.926	4.75E−04
970	6470	*SHMT1*	0.262	30.913	4.78E−04
971	1.01E+08	*MMP24OS*	−0.390	30.910	4.79E−04
972	3148	*HMGB2*	0.264	30.910	4.79E−04
973	8840	*CCN4*	0.289	30.879	4.87E−04
974	319089	*TTC6*	0.257	30.874	4.88E−04
975	55902	*ACSS2*	0.275	30.838	4.97E−04
976	3516	*RBPJ*	0.310	30.834	4.98E−04
977	23303	*KIF13B*	−0.269	30.831	4.99E−04
978	23199	*GSE1*	0.270	30.826	5.00E−04
979	29093	*MRPL22*	0.261	30.814	5.03E−04
980	3895	*KTN1*	−0.398	30.762	5.17E−04
981	11001	*SLC27A2*	0.256	30.757	5.18E−04
982	64769	*MEAF6*	−0.271	30.726	5.27E−04
983	55225	*RAVER2*	0.281	30.708	5.32E−04
984	4879	*NPPB*	−0.275	30.705	5.32E−04
985	284739	*C20orf204*	0.705	30.680	5.39E−04
986	6202	*RPS8*	0.254	30.660	5.45E−04
987	340419	*RSPO2*	0.272	30.631	5.53E−04
988	768211	*RELL1*	−0.272	30.614	5.58E−04
989	144363	*ETFRF1*	0.276	30.582	5.67E−04
990	83930	*STARD3NL*	−0.288	30.576	5.69E−04
991	8507	*ENC1*	−0.265	30.568	5.71E−04
992	56672	*AKIP1*	−0.255	30.567	5.72E−04
993	64856	*VWA1*	−0.343	30.561	5.73E−04
994	80222	*TARS2*	0.250	30.559	5.74E−04
995	90134	*KCNH7*	−0.280	30.543	5.79E−04
996	53349	*ZFYVE1*	−0.267	30.539	5.80E−04
997	3460	*IFNGR2*	−0.278	30.533	5.82E−04
998	727936	*GXYLT2*	0.301	30.526	5.84E−04
999	9747	*TCAF1*	−0.364	30.526	5.84E−04
1000	4603	*MYBL1*	−0.268	30.520	5.86E−04
1001	445328	*ARHGEF35*	−0.290	30.496	5.93E−04
1002	220441	*RNF152*	0.428	30.486	5.96E−04
1003	8694	*DGAT1*	0.337	30.472	6.00E−04

1004	54331	*GNG2*	0.279	30.432	6.13E−04
1005	114794	*ELFN2*	−0.271	30.426	6.15E−04
1006	1613	*DAPK3*	−0.420	30.408	6.21E−04
1007	162387	*MFSD6L*	0.261	30.406	6.21E−04
1008	138050	*HGSNAT*	−0.277	30.399	6.23E−04
1009	51310	*SLC22A17*	0.366	30.398	6.24E−04
1010	5972	*REN*	0.258	30.394	6.25E−04
1011	9788	*MTSS1*	0.258	30.384	6.28E−04
1012	1742	*DLG4*	0.338	30.352	6.39E−04
1013	54842	*MFSD6*	−0.284	30.327	6.47E−04
1014	11077	*HSF2BP*	−0.251	30.285	6.61E−04
1015	1.01E+08	*LOC100506178*	0.260	30.282	6.62E−04
1016	257629	*ANKS4B*	0.282	30.281	6.62E−04
1017	51011	*FAHD2A*	0.346	30.261	6.69E−04
1018	8835	*SOCS2*	−0.276	30.254	6.72E−04
1019	8544	*PIR*	0.268	30.254	6.72E−04
1020	55625	*ZDHHC7*	−0.293	30.247	6.74E−04
1021	23209	*MLC1*	0.262	30.238	6.77E−04
1022	9455	*HOMER2*	−0.268	30.228	6.81E−04
1023	3762	*KCNJ5*	0.303	30.220	6.84E−04
1024	1556	*CYP2B7P*	0.250	30.177	6.99E−04
1025	27348	*TOR1B*	−0.266	30.175	7.00E−04
1026	10615	*SPAG5*	0.263	30.170	7.02E−04
1027	55809	*TRERF1*	0.283	30.139	7.13E−04
1028	10156	*RASA4*	0.298	30.040	7.50E−04
1029	1E+08	*IPO5P1*	−0.269	30.034	7.53E−04
1030	9263	*STK17A*	−0.309	30.014	7.60E−04
1031	55856	*ACOT13*	0.264	30.010	7.62E−04
1032	200942	*KLHDC8B*	0.300	30.003	7.65E−04
1033	656	*BMP8B*	−0.268	29.982	7.73E−04
1034	377	*ARF3*	−0.257	29.961	7.81E−04
1035	2147	*F2*	0.277	29.932	7.93E−04
1036	79611	*ACSS3*	0.276	29.910	8.02E−04
1037	5293	*PIK3CD*	−0.268	29.908	8.03E−04
1038	684	*BST2*	0.258	29.906	8.04E−04
1039	65010	*SLC26A6*	0.286	29.877	8.16E−04
1040	55800	*SCN3B*	−0.258	29.853	8.26E−04
1041	517	*ATP5MC2*	0.273	29.830	8.36E−04
1042	29122	*PRSS50*	0.298	29.812	8.44E−04
1043	401233	*HTATSF1P2*	0.277	29.800	8.49E−04
1044	27020	*NPTN*	−0.298	29.784	8.56E−04
1045	4026	*LPP*	−0.377	29.773	8.61E−04
1046	3301	*DNAJA1*	−0.409	29.749	8.72E−04
1047	23370	*ARHGEF18*	−0.296	29.728	8.81E−04
1048	93594	*TBC1D31*	0.268	29.727	8.81E−04
1049	51088	*KLHL5*	−0.303	29.691	8.98E−04
1050	30811	*HUNK*	0.297	29.690	8.99E−04
1051	355	*FAS*	−0.268	29.671	9.07E−04
1052	84656	*GLYR1*	−0.261	29.624	9.30E−04
1053	8772	*FADD*	−0.338	29.617	9.33E−04
1054	79630	*C1orf54*	0.258	29.612	9.35E−04
1055	377047	*PRSS45P*	0.255	29.612	9.36E−04
1056	8573	*CASK*	−0.307	29.601	9.41E−04

1057	138151	*NACC2*	−0.278	29.599	9.42E−04
1058	1212	*CLTB*	−0.330	29.578	9.52E−04
1059	66000	*TMEM108*	−0.255	29.573	9.54E−04
1060	23179	*RGL1*	−0.301	29.569	9.56E−04
1061	7145	*TNS1*	0.260	29.564	9.59E−04
1062	2619	*GAS1*	0.281	29.557	9.63E−04
1063	64857	*PLEKHG2*	−0.319	29.550	9.66E−04
1064	1203	*CLN5*	−0.286	29.548	9.67E−04
1065	54014	*BRWD1*	0.278	29.546	9.68E−04
1066	3215	*HOXB5*	0.288	29.536	9.73E−04
1067	1562	*CYP2C18*	0.250	29.517	9.82E−04
1068	24140	*FTSJ1*	−0.250	29.517	9.82E−04
1069	85236	*H2BC12*	−0.258	29.511	9.85E−04
1070	78989	*COLEC11*	0.250	29.495	9.94E−04
