# Supplementary material for: Transcriptomic Architecture of Metabolic Dysfunction-Associated Steatotic Liver Disease (MASLD) Risk in Mexican Americans
Source: Cells. 2026 Sep 1;15(17):1592. doi: 10.3390/cells15171592 (PMC13565466; doi:10.3390/cells15171592)
Supplement: Supplementary file 1 [file cells-15-01592-s001.zip › Supplementary Table S2.pdf]

Supplementary Table S2: Functional annotation of key genes whose expression was directly correlated with baseline steatosis measures.

Gene Symbol	Functional Category	Gene Function
DGAT1, DGAT2	Lipid Metabolism and Lipogenesis	Catalyze the final, rate-limiting step in triglyceride synthesis.
FABP1, SLC27A2	Lipid Metabolism and Lipogenesis	Uptake and intracellular transporter of long-chain fatty acids in hepatocytes.
STARD4	Lipid Metabolism and Lipogenesis	Intracellular cholesterol transport.
MLXIPL (ChREBP)	Lipid Metabolism and Lipogenesis	Major transcription factor that activates glycolytic and lipogenic genes in response to high glucose.
ELOVL6	Lipid Metabolism and Lipogenesis	Elongates saturated and monounsaturated fatty acids, contributing to the lipogenic pool.
SOAT2 (ACAT2)	Lipid Metabolism and Lipogenesis	Packages cholesterol into cholesteryl esters for lipid droplet or VLDL assembly.
MTTP	Lipid Metabolism and Lipogenesis	Critical for assembling and secreting ApoB-containing lipoproteins.
ACACB	Mitochondrial Function and Beta-Oxidation	Involved in the regulation and breakdown of fatty acids via beta-oxidation.
HMGCS2	Mitochondrial Function and Beta-Oxidation	Serves as the rate-limiting enzyme for ketogenesis; involved in breaking down fatty acids.
HADH, EHHADH, ACAD11, ACOX2	Mitochondrial Function and Beta-Oxidation	Enzymes involved in mitochondrial and peroxisomal fatty acid beta-oxidation.
ALDOB	Pyruvate and Glucose Shunts	Involved in intense carbohydrate processing.
FBP1	Pyruvate and Glucose Shunts	Involved in gluconeogenesis and carbohydrate processing.
KHK	Pyruvate and Glucose Shunts	Primary enzyme in fructose metabolism.
PDK3	Pyruvate and Glucose Shunts	Inhibits the pyruvate dehydrogenase complex to turn off glucose oxidation and divert carbons to lipogenesis.
MPC2	Mitochondrial Carrier Proteins	Essential transport machinery component.
SLC25A42	Mitochondrial Carrier Proteins	Essential mitochondrial transport machinery component.
OXA1L	Mitochondrial Carrier Proteins	Structural element of the mitochondrial transport machinery.
CXCL14, CXCL13	Inflammatory Signaling and Chemokines	Recruit immune cells (B cells, macrophages, T cells) to steatotic liver tissue.
CD163	Immune Infiltration and Activation	Marker for M2-polarized macrophages and Kupffer cells, indicating immune infiltration.
CD83	Immune Infiltration and Activation	Marker indicating the activation of antigen-presenting cells.
PTCH1, PTCH2	Hedgehog Signaling	Receptors reactivated during liver injury and hepatocyte ballooning.
IHH	Hedgehog Signaling	Ligand that drives the reactivated Hedgehog pathway during injury.
GLI1	Hedgehog Signaling	Downstream transcription factor/effector of the reactivated Hedgehog signaling cascade.

FGFR1	Growth Factors and Receptors	Regulates downstream cascades involved in cell survival and differentiation.
IGFBP3, IG2BP2	Growth Factors and Receptors	Insulin-like growth factor binding proteins that modulate metabolic homeostasis and cell proliferation.
CEBPA	Transcriptional Regulators	Master transcription factor regulating adipogenesis and lipid homeostasis.
NR4A1	Transcriptional Regulators	Transcription factor indicating increased cellular stress response.
DDIT3 (CHOP)	Transcriptional Regulators	Marker for endoplasmic reticulum (ER) stress.

Note: Gene function annotation resources used: Gene Cards – the human gene database; NCBI gene webpage; KEGG gene and pathway databases; IPA knowledgebase; and PubMed literature search (accessed in July 2026).
