# Supplementary material for: Transcriptomic Architecture of Metabolic Dysfunction-Associated Steatotic Liver Disease (MASLD) Risk in Mexican Americans
Source: Cells. 2026 Sep 1;15(17):1592. doi: 10.3390/cells15171592 (PMC13565466; doi:10.3390/cells15171592)
Supplement: Supplementary file 1 [file cells-15-01592-s001.zip › Supplementary Table S3.pdf]

Supplementary Table S3: Functional annotation of key genes whose baseline expression was inversely correlated with baseline hepatic steatosis measures.

Gene Symbol	Functional Category	Gene Function
NPC1	Non-Vesicular Transport	Mediates LDL-derived cholesterol efflux out of lysosomes.
ESYT1	Non-Vesicular Transport	Tethers ER to the plasma membrane and Ca^{2+}-dependent transfer of phospholipids.
STARD3NL	Non-Vesicular Transport	Tethers late endosomes to the ER to facilitate cholesterol movement.
TTPAL	Non-Vesicular Transport	Chaperone involved in binding and transporting tocopherol (vitamin E).
ATP10A	Lipid Flippases, Scramblases, and Metabolism	Flippase that translocates aminophospholipids to maintain membrane asymmetry.
PLSCR3	Lipid Flippases, Scramblases, and Metabolism	Scramblase that disrupts mitochondrial membrane asymmetry for cardiolipin remodeling/apoptosis.
SEC14L1	Lipid Flippases, Scramblases, and Metabolism	Cytosolic phospholipid transfer protein regulating lipid pools during trafficking.
RAB5C	Vesicular Carrier Dynamics and Rab GTPases	Rab GTPase that regulates early endosome fusion.
RAB11FIP1	Vesicular Carrier Dynamics and Rab GTPases	Rab11 effector governing recycling of lipid-rich membranes to the cell surface.
EHD1	Vesicular Carrier Dynamics and Rab GTPases	ATP-driven membrane-remodeling protein that tubulates membranes for vesicle pinching.
EHD4	Vesicular Carrier Dynamics and Rab GTPases	ATP-driven membrane-remodeling protein that tubulates membranes for vesicle pinching.
ACTB, ACTG1, ACTA1	Actin Cytoskeleton (Structural)	Structural building block of microfilament tracks.
ACTR3, ARPC4	Actin Cytoskeleton (Nucleation and Branching)	Core component of the Arp2/3 complex; generates branched actin networks.
CFL1	Actin Cytoskeleton (Severing and Capping)	Sever actin filaments to maintain a dynamic actin pool.
PFN1	Actin Cytoskeleton (Severing and Capping)	Promotes actin polymerization.
FLNA, FLNB	Actin Cytoskeleton (Crosslinkers and Adaptors)	Filamin protein anchors actin networks to membranes.
TLN1, VASP	Actin Cytoskeleton (Crosslinkers and Adaptors)	Adaptor proteins that anchor actin networks to membranes.
SPTAN1	Actin Cytoskeleton (Crosslinkers and Adaptors)	Spectrin protein anchors actin networks to membranes.
TUBA1A, TUBB3, TUBB6	Microtubules (Structural)	Tubulins form rigid microtubule protofilaments.
KIF13B, KIF3C	Anterograde Motors	Kinesin motor proteins move lipid vesicles towards the microtubule plus end.
DYNLT1, DCTN1	Retrograde Motors	Dynein transports cargo toward microtubule minus-ends.

VIM	Intermediate Filaments	Vimentin provides mechanical stability and anchors cytoplasmic LDL-cholesterol droplets.
KRT6A, KRT6B, KRT6C, KRT7, KRT8, KRT18	Intermediate Filaments	Keratin maintains structural integrity and modulates lipid droplet distribution.
MYO5B, MYO1E	Vesicle Motility	Myosin motor proteins bind to recycling endosome lipids/proteins to move them along actin tracks.
KTN1	Vesicle Motility	Kinectin links the ER membrane to kinesin motors.
PACSIN2	Membrane Curvature and Invagination	Contains a BAR domain that senses membrane lipid curvature and binds to both the actin-regulating dynamin complex and the lipid membrane to coordinate endocytosis.
CAV2	Membrane Curvature and Invagination	Forms lipid rafts
FLNA, SPTAN1	Lipid Raft Scaffolding	Filamin and Spectrin tether cholesterol-rich domains to the underlying actin skeleton to stabilize membrane architecture.

Note: Gene function annotation resources used: Gene Cards – the human gene database; NCBI gene webpage; KEGG gene and pathway databases; IPA knowledgebase; and PubMed literature search (accessed in July 2026).
